# The Concise Guide to PHARMACOLOGY 2015/16: Transporters

**DOI:** 10.1111/bph.13355

**Published:** 2015-12-09

**Authors:** Stephen PH Alexander, Eamonn Kelly, Neil Marrion, John A Peters, Helen E Benson, Elena Faccenda, Adam J Pawson, Joanna L Sharman, Christopher Southan, Jamie A Davies, CGTP Collaborators

**Affiliations:** ^1^ School of Biomedical Sciences University of Nottingham Medical School Nottingham NG7 2UH UK; ^2^ School of Physiology and Pharmacology University of Bristol Bristol BS8 1TD UK; ^3^ Neuroscience Division Medical Education Institute, Ninewells Hospital and Medical School, University of Dundee Dundee DD1 9SY UK; ^4^ Centre for Integrative Physiology University of Edinburgh Edinburgh EH8 9XD UK

## Abstract

The Concise Guide to PHARMACOLOGY 2015/16 provides concise overviews of the key properties of over 1750 human drug targets with their pharmacology, plus links to an open access knowledgebase of drug targets and their ligands (www.guidetopharmacology.org), which provides more detailed views of target and ligand properties. The full contents can be found at http://onlinelibrary.wiley.com/doi/10.1111/bph.13355/full. G protein‐coupled receptors are one of the eight major pharmacological targets into which the Guide is divided, with the others being: G protein‐coupled receptors, ligand‐gated ion channels, voltage‐gated ion channels, other ion channels, nuclear hormone receptors, catalytic receptors and transporters. These are presented with nomenclature guidance and summary information on the best available pharmacological tools, alongside key references and suggestions for further reading. The Concise Guide is published in landscape format in order to facilitate comparison of related targets. It is a condensed version of material contemporary to late 2015, which is presented in greater detail and constantly updated on the website www.guidetopharmacology.org, superseding data presented in the previous Guides to Receptors & Channels and the Concise Guide to PHARMACOLOGY 2013/14. It is produced in conjunction with NC‐IUPHAR and provides the official IUPHAR classification and nomenclature for human drug targets, where appropriate. It consolidates information previously curated and displayed separately in IUPHAR‐DB and GRAC and provides a permanent, citable, point‐in‐time record that will survive database updates.

## Conflict of interest

The authors state that there are no conflicts of interest to declare.

## Overview

The majority of biological solutes are charged organic or inorganic molecules. Cellular membranes are hydrophobic and, therefore, effective barriers to separate them allowing the formation of gradients, which can be exploited, for example, in the generation of energy. Membrane transporters carry solutes across cell membranes, which would otherwise be impermeable to them. The energy required for active transport processes is obtained from ATP turnover or by exploiting ion gradients.

ATP‐driven transporters can be divided into three major classes: P‐type ATPases; F‐type or V‐type ATPases and ATP‐binding cassette transporters. The first of these, P‐type ATPases, are multimeric proteins, which transport (primarily) inorganic cations. The second, F‐type or V‐type ATPases, are proton‐coupled motors, which can function either as transporters or as motors. Last, are ATP‐binding cassette transporters, heavily involved in drug disposition as well as transporting endogenous solutes.

The second largest family of membraine proteins in the human genome, after the G protein‐coupled receptors, are the SLC solute carrier family. Within the solute carrier family, there are not only a great variety of solutes transported, from simple inorganic ions to amino acids and sugars to relatively complex organic molecules like haem. The solute carrier family includes 52 families of almost 400 members. Many of these overlap in terms of the solutes that they carry. For example, amino acids accumulation is mediated by members of the SLC1, SLC3/7, SLC6, SLC15, SLC16, SLC17, SLC32, SLC36, SLC38 and SLC43 families. Further members of the SLC superfamily regulate ion fluxes at the plasma membrane, or solute transport into and out of cellular organelles. Some SLC family members remain orpahn transporters, in as much as a physiological function has yet to be dtermined. Within the SLC superfamily, there is an abundance in diversity of structure. Two families (SLC3 and SLC7) only generate functional transporters as heteromeric partners, where one partner is a single TM domain protein. Membrane topology predictions for other families suggest 3,4,6,7,8,9,10,11,12,13 or 14 TM domains. The SLC transporters include members which function as antiports, where solute movement in one direction is balanced by a solute moving in the reverse direction. Symports allow concentration gradients of one solute to allow movement of a second solute across a membrane. A third, relatively small group are equilibrative transporters, which allow solutes to travel across membranes down their concentration gradients. A more complex family of transporters, the SLC27 fatty acid transporters also express enzymatic function. Many of the transporters also express electrogenic properties of ion channels.

## Family structure

This is a complete listing of transporter families included in the online IUPHAR/BPS Guide to PHARMACOLOGY database. Summary information is provided here for a subset of transporters where these are of significant pharmacological interest; further transporters are listed in the database

6113 ATP‐binding cassette transporter family

6113 ABCA subfamily

6115 ABCB subfamily

6116 ABCC subfamily

6117 ABCD subfamily of peroxisomal ABC transporters

6118 ABCG subfamily

6119 F‐type and V‐type ATPases

6119 F‐type ATPase

6120 V‐type ATPase

6120 P‐type ATPases

6121 Na^+^/K^+^‐ATPases

6121 Ca^2+^‐ATPases

6122 H^+^/K^+^‐ATPases

6122 Cu^+^‐ATPases

6122 Phospholipid‐transporting ATPases

6123 Major facilitator superfamily (MFS) of transporters

6123 SLC superfamily of solute carriers

6124 SLC1 family of amino acid transporters

6124 Glutamate transporter subfamily

6126 Alanine/serine/cysteine transporter subfamily

6127 SLC2 family of hexose and sugar alcohol

6127 Class I transporters

6128 Class II transporters

6129 Proton‐coupled inositol transporter

6129 SLC3 and SLC7 families of heteromeric amino acid transporters (HATs)

6130 SLC3 family

6130 SLC7 family

6131 SLC4 family of bicarbonate transporters

6132 Anion exchangers

6132 Sodium‐dependent HCO ${}_{\text{3}}^{\text{‐}}$ transporters

6133 SLC5 family of sodium‐dependent glucose transporters

6134 Hexose transporter family

6135 Choline transporter

6136 Sodium iodide symporter, sodium‐dependent multivitamin transporter and sodium‐coupled monocarboxylate transporters

6137 Sodium *myo*‐inositol cotransporter transporters

6138 SLC6 neurotransmitter transporter family

6138 Monoamine transporter subfamily

6139 GABA transporter subfamily

6141 Glycine transporter subfamily

6142 Neutral amino acid transporter subfamily

6144 SLC8 family of sodium/calcium exchangers

6145 SLC9 family of sodium/hydrogen exchangers

6145 SLC10 family of sodium‐bile acid co‐transporters

6147 SLC11 family of proton‐coupled metal ion transporters

6148 SLC12 family of cation‐coupled chloride transporters

6149 SLC13 family of sodium‐dependent sulphate/carboxylate transporters

6150 SLC14 family of facilitative urea transporters

6151 SLC15 family of peptide transporters

6152 SLC16 family of monocarboxylate transporters

6154 SLC17 phosphate and organic anion transporter

6154 family

6154 Type I sodium‐phosphate co‐transporters

6155 Sialic acid transporter

6155 Vesicular glutamate transporters (VGLUTs)

6156 Vesicular nucleotide transporter

6156 SLC18 family of vesicular amine transporters

6158 SLC19 family of vitamin transporters

6159 SLC20 family of sodium‐dependent phosphate transporters

6160 SLC22 family of organic cation and anion transporters

6160 Organic cation transporters (OCT)

6161 Organic zwitterions/cation transporters (OCTN)

6162 Organic anion transporters (OATs)

6163 Urate transporter

– Orphan or poorly characterized SLC22 family members

6163 SLC23 family of ascorbic acid transporters

6164 SLC24 family of sodium/potassium/calcium exchangers

6165 SLC25 family of mitochondrial transporters

6165 Mitochondrial di‐ and tri‐carboxylic acid transporter subfamily

6166 Mitochondrial amino acid transporter subfamily

6167 Mitochondrial phosphate transporters

6167 Mitochondrial nucleotide transporter subfamily

6168 Mitochondrial uncoupling proteins

6169 Miscellaneous SLC25 mitochondrial transporters

6170 SLC26 family of anion exchangers

6170 Selective sulphate transporters

6170 Chloride/bicarbonate exchangers

6171 Anion channels

6171 Other SLC26 anion exchangers

6172 SLC27 family of fatty acid transporters

6173 SLC28 and SLC29 families of nucleoside transporters

6173 SLC28 family

6174 SLC29 family

6176 SLC30 zinc transporter family

6176 SLC31 family of copper transporters

6177 SLC32 vesicular inhibitory amino acid transporter

6178 SLC33 acetylCoA transporter

6179 SLC34 family of sodium phosphate co‐transporters

6180 SLC35 family of nucleotide sugar transporters

6181 SLC36 family of proton‐coupled amino acid transporters

6182 SLC37 family of phosphosugar/phosphate exchangers

6182 SLC38 family of sodium‐dependent neutral amino

6182 acid transporters

6183 System A‐like transporters

6183 System N‐like transporters

6184 Orphan SLC38 transporters

6185 SLC39 family of metal ion transporters

6186 SLC40 iron transporter

6187 SLC41 family of divalent cation transporters

6187 SLC42 family of Rhesus glycoprotein ammonium transporters

6188 SLC43 family of large neutral amino acid transporters

6189 SLC44 choline transporter‐like family

6190 SLC45 family of putative sugar transporters

6191 SLC46 family of folate transporters

6192 SLC47 family of multidrug and toxin extrusion transporters

6192 SLC48 heme transporter

6193 SLC49 family of FLVCR‐related heme transporters

6194 SLC50 sugar transporter

6195 SLC51 family of steroid‐derived molecule transporters

6195 SLC52 family of riboflavin transporters

6196 SLCO family of organic anion transporting polypeptides

## ATP‐binding cassette transporter family

### Overview

ATP‐binding cassette transporters are ubiquitous membrane proteins characterized by active ATP‐dependent movement of a range of substrates, including ions, lipids, peptides, steroids. Individual subunits are typically made up of two groups of 6TM‐spanning domains, with two nucleotide‐binding domains (NBD). The majority of eukaryotic ABC transporters are ‘full’ transporters incorporating both TM and NBD entities. Some ABCs, notably the ABCD and ABCG families are half‐transporters with only a single membrane spanning domain and one NBD, and are only functional as homo‐ or heterodimers. Eukaryotic ABC transporters convey substrates from the cytoplasm, either out of the cell or into intracellular organelles. Their role in the efflux of exogenous compounds, notably chemotherapeutic agents, has led to considerable interest.

### Comments

A further group of ABC transporter‐like proteins have been identified to lack membrane spanning regions and are not believed to be functional transporters, but appear to have a role in protein translation [88, 380]: ABCE1(P61221, also known as OABP or 2'‐5' oligoadenylate‐binding protein); ABCF1(Q8NE71, also known as ABC50 or TNF‐*α*‐stimulated ABC protein); ABCF2(Q9UG63, also known as iron‐inhibited ABC transporter 2) and ABCF3(Q9NUQ8).

## ABCA subfamily

**Table:** 

Nomenclature	ABCA1	ABCA3	ABCA4	ABCA5
Common abreviation	ABC1, CERP	ABC3, ABCC	ABCR	–
HGNC, UniProt	ABCA1, O95477	ABCA3, Q99758	ABCA4, P78363	ABCA5, Q8WWZ7
Selective inhibitors	probucol [156, 520]	–	–	–
Comments	Loss‐of‐function mutations are associated with Tangier disease, in which plasma HDL cholesterol levels are greatly reduced. ABCA1 is a key player in cholesterol efflux from macrophages to lipid‐free apo‐A1 in a process known as reverse cholesterol transport, a role that is important in atherosclerosis. ABCA1 also controls apoE lipidation, and has a role in Alzheimer's disease, including an impact on amyloid *β* (APP, P05067) deposition and clearance. ABCA1 is transcriptionally regulated by Liver X Receptors (LXR) and Retinoic X Receptor (RXR), which are being explored as therapeutic targets for development of agonists for treatment of metabolic and neurodegenerative disorders [286].	Loss‐of‐function mutations are associated with pulmonary surfactant deficiency	Retinal‐specific transporter of N‐retinylPE; loss‐of‐function mutations are associated with childhood‐onset Stargardt disease, a juvenile onset macular degenerative disease. The earlier onset disease is often associated with the more severe and deleterious ABCA4 variants [173].	
ABCA4 facilitates the clearance of all‐trans‐retinal from photoreceptor disc membranes following photoexcitation. ABCA4 can also transport N‐11‐cis‐retinylidene‐phosphatidylethanolamine, the Schiff‐base adduct of 11‐cis‐retinal; loss of function mutation cause a buildup of lipofuscin, atrophy of the central retina, and severe progressive loss in vision [394].	ABCA5 is a lysosomal protein whose loss of function compromises integrity of lysosomes and leads to intra‐endolysosomal accumulation of cholesterol. It has recently been associated with Congenital Generalized Hypertrichosis Terminalis (CGHT), a hair overgrowth syndrome, in a patient with a mutation in ABCA5 that significantly decreased its expression [113].			

**Table:** 

Nomenclature	ABCA6	ABCA7	ABCA12
HGNC, UniProt	ABCA6, Q8N139	ABCA7, Q8IZY2	ABCA12, Q86UK0
Comments	A recent genome wide association study identified an ABCA6 variant associated with cholesterol levels [557].	Genome wide association studies identify ABCA7 variants as associated with Alzheimer's Disease [232].	Reported to play a role in skin ceramide formation [555]. A recent study shows that ABCA12 expression also impacts cholesterol efflux from macrophages. ABCA12 is postulated to associate with ABCA1 and LXR beta, and stabilize expression of ABCA1. ABCA12 deficiency causes decreased expression of Abca1, Abcg1 and Nr1h2 [171].

### Comments

A number of structural analogues are not found in man: Abca14 (ENSMUSG00000062017); Abca15 (ENSMUSG00000054746); Abca16 (ENSMUSG00000051900) and Abca17 (ENSMUSG00000035435).

## ABCB subfamily

**Table:** 

Nomenclature	ABCB1	ABCB2	ABCB3	ABCB4	ABCB5
Common abreviation	MDR1, PGP1	TAP1	TAP2	PGY3	–
HGNC, UniProt	ABCB1, P08183	TAP1, Q03518	TAP2, Q03519	ABCB4, P21439	ABCB5, Q2M3G0
Comments	Responsible for the cellular export of many therapeutic drugs. The mouse and rat have two Abcb1 genes (gene names; Abcb1a and Abcb1b) while the human has only the one gene, ABCB1.	Endoplasmic reticulum peptide transporter, possibly requires heterodimerdimerization with TAP2.	Endoplasmic reticulum peptide transporter, possibly as requires heterodimerization with TAP1.	Transports phosphatidylcholine from intracellular to extracellular face of the hepatocyte canalicular membrane [375]	Multidrug resistance protein in, and marker of, melanoma cells [433]

**Table:** 

Nomenclature	ABCB6	ABCB7	ABCB8	ABCB9	ABCB10	ABCB11
Common abreviation	MTABC3	ABC7	MABC1	TAPL	MTABC2	ABC16
HGNC, UniProt	ABCB6, Q9NP58	ABCB7, O75027	ABCB8, Q9NUT2	ABCB9, Q9NP78	ABCB10, Q9NRK6	ABCB11, O95342
Comments	Putative mitochondrial porphyrin transporter [290]; other subcellular localizations are possible, such as the plasma membrane, as a specific determinant of the Langereis blood group system [227].	Mitochondrial; reportedly essential for haematopoiesis [388]	Mitochondrial; suggested to play a role in chemoresistance of melanoma [142]	Reported to be lysosomal [260]	Mitochondrial location; the first human ABC transporter to have a crystal structure reported [448].	Loss‐of‐function mutations are associated with progressive familial intrahepatic cholestasis type 2 [456]

## ABCC subfamily

**Table:** 

Nomenclature	ABCC1	ABCC2	ABCC3	ABCC4	ABCC5	ABCC6
Common abreviation	MRP1	MRP2, cMOAT	MRP3	MRP4	MRP5	MRP6
HGNC, UniProt	ABCC1, P33527	ABCC2, Q92887	ABCC3, O15438	ABCC4, O15439	ABCC5, O15440	ABCC6, O95255
Comments	Exhibits a broad substrate specificity [26], including LTC_4_ (K_m_ 97 nM [309]) and estradiol‐17*β*‐glucuronide [460].	Loss‐of‐function mutations are associated with Dubin‐Johnson syndrome, in which plasma levels of conjugated bilirubin are elevated (OMIM: 237500).	Transports conjugates of glutathione, sulfate or glucuronide [51]	Although reported to facilitate cellular cyclic nucleotide export, this role has been questioned [51]; reported to export prostaglandins in a manner sensitive to NSAIDS [403]	Although reported to facilitate cellular cyclic nucleotide export, this role has been questioned [51]	Loss‐of‐function mutations in ABCC6 are associated with pseudoxanthoma elasticum (OMIM: 264800).

**Table:** 

Nomenclature	ATP‐binding cassette, sub‐family C(CFTR/MRP), member 8	ABCC9	ABCC11
Systematic nomenclature	ABCC8	–	–
Common abreviation	SUR1	SUR2	MRP8
HGNC, UniProt	ABCC8, Q09428	ABCC9, O60706	ABCC11, Q96J66
Selective inhibitors	repaglinide (pIC_50_ 7) [523]	–	–
Comments	The sulfonyurea drugs (acetohexamide, tolbutamide and glibenclamide) appear to bind sulfonylurea receptors and it has been shown experimentally that tritiated glibenclamide can be used to pull out a 140 kDa protein identified as SUR1 (now known as ABCC8) [402]. SUR2 (ABCC9) has also been identified [241]. However, this is not the full mechanism of action and the functional channel has been characterised as a hetero‐octamer formed by four SUR and four K_ir_6.2 subunits, with the K_ir_6.2 subunits forming the core ion pore and the SUR subunits providing the regulatory properties [345]. Co‐expression of K_ir_6.2 with SUR1, reconstitutes the ATP‐dependent K^+^ conductivity inhibited by the sulfonyureas [241].	Associated with familial atrial fibrillation, Cantu syndrome and familial isolated dilated cardiomyopathy.	Single nucleotide polymorphisms distinguish wet vs. dry earwax (OMIM: 117800); an association between earwax allele and breast cancer risk is reported in Japanese but not European populations.

### Comments

ABCC7 (also known as CFTR, a 12TM ABC transporter‐type protein, is a cAMP‐regulated epithelial cell membrane Cl^‐^ channel involved in normal fluid transport across various epithelia and can be viewed in the Chloride channels section of the Guide. ABCC8 (ENSG00000006071, also known as SUR1, sulfonylurea receptor 1) and ABCC9 (ENSG00000069431, also known as SUR2, sulfonylurea receptor 2) are unusual in that they lack transport capacity but regulate the activity of particular K^+^ channels (Kir6.1‐6.2), conferring nucleotide sensitivity to these channels to generate the canonical K_ATP_ channels. ABCC13 (ENSG00000155288) is a possible pseudogene.

## ABCD subfamily of peroxisomal ABC transporters

### Overview

This family of ‘half‐transporters’ act as homo‐ or heterodimers to accumulate fatty acid‐CoA esters into peroxisomes for oxidative metabolism [273].

**Table:** 

Nomenclature	ABCD1	ABCD2	ABCD3
Common abreviation	ALDP	ALDR	PMP70
HGNC, UniProt	ABCD1, P33897	ABCD2, Q9UBJ2	ABCD3, P28288
Comments	Transports coenzyme A esters of very long chain fatty acids [558, 559]; loss‐of‐function mutations in ABCD1 are associated with adrenoleukodystrophy (OMIM: 3001002).	Coenzyme A esters of very long chain unsaturated fatty acids [558]	–

### Comments

ABCD4 (ENSG00000119688, also known as PMP69, PXMP1‐L or P70R) appears to be located on the endoplasmic reticulum [271], with an unclear function. Loss‐of‐function mutations in the gene encoding ALDP underlie the metabolic storage disorder X‐linked adrenoleukodystrophy.

### Further Reading

Aye IL et al. (2009) Transport of lipids by ABC proteins: interactions and implications for cellular toxicity, viability and function. Chem. Biol. Interact. 180: 327‐39 [PMID:19426719]

Gutmann DA et al. (2010) Understanding polyspecificity of multidrug ABC transporters: closing in on the gaps in ABCB1. Trends Biochem. Sci. 35: 36‐42 [PMID:19819701]

Haenisch S et al. (2014) MicroRNAs and their relevance to ABC transporters. Br J Clin Pharmacol 77: 587‐96 [PMID:24645868]

Hinz A et al. (2012) ABC transporters and immunity: mechanism of self‐defense. Biochemistry 51: 4981‐9 [PMID:22668350]

Kemp S et al. (2011) Mammalian peroxisomal ABC transporters: from endogenous substrates to pathology and clinical significance. Br. J. Pharmacol. 164: 1753‐66 [PMID:21488864]

Kerr ID et al. (2011) The ABCG family of membrane‐associated transporters: you don't have to be big to be mighty. Br. J. Pharmacol. 164: 1767‐79 [PMID:21175590]

López‐Marqués RL et al. (2015) Structure and mechanism of ATP‐dependent phospholipid transporters. Biochim. Biophys. Acta 1850: 461‐475 [PMID:24746984]

Miller DS. (2010) Regulation of P‐glycoprotein and other ABC drug transporters at the blood‐brain barrier. Trends Pharmacol. Sci. 31: 246‐54 [PMID:20417575]

Nishi T et al. (2014) Molecular and physiological functions of sphingosine 1‐phosphate transporters. Biochim. Biophys. Acta 1841: 759‐65 [PMID:23921254]

Phillips MC. (2014) Molecular mechanisms of cellular cholesterol efflux. J. Biol. Chem. 289: 24020‐9 [PMID:25074931]

Procko E et al. (2009) The mechanism of ABC transporters: general lessons from structural and functional studies of an antigenic peptide transporter. FASEB J. 23: 1287‐302 [PMID:19174475]

Ravna AW et al. (2009) Molecular modeling studies of ABC transporters involved in multidrug resistance. Mini Rev Med Chem 9: 186‐93 [PMID:19200023]

Rees DC et al. (2009) ABC transporters: the power to change. Nat. Rev. Mol. Cell Biol. 10: 218‐27 [PMID:19234479]

Seeger MA et al. (2009) Molecular basis of multidrug transport by ABC transporters. Biochim. Biophys. Acta 1794: 725‐37 [PMID:19135557]

Stacy AE et al. (2013) Molecular pharmacology of ABCG2 and its role in chemoresistance. Mol. Pharmacol. 84: 655‐69 [PMID:24021215]

Tarling EJ et al. (2013) Role of ABC transporters in lipid transport and human disease. Trends Endocrinol. Metab. 24: 342‐50 [PMID:23415156]

Westerterp M et al. (2014) ATP‐binding cassette transporters, atherosclerosis, and inflammation. Circ. Res. 114: 157‐70 [PMID:24385509]

Wong K et al. (2014) Towards understanding promiscuity in multidrug efflux pumps. Trends Biochem. Sci. 39: 8‐16 [PMID:24316304]

## ABCG subfamily

### Overview

This family of ‘half‐transporters’ act as homo‐ or heterodimers; particularly ABCG5 and ABCG8 are thought to be obligate heterodimers. They are associated with cellular export of sterols and phospholipids, as well as exogenous drugs (ABCG2).

**Table:** 

Nomenclature	ABCG1	ABCG2	ABCG4	ABCG5	ABCG8
Common abreviation	ABC8	ABCP	–	–	–
HGNC, UniProt	ABCG1, P45844	ABCG2, Q9UNQ0	ABCG4, Q9H172	ABCG5, Q9H222	ABCG8, Q9H221
Comments	Transports sterols and choline phospholipids [275]	Exhibits a broad substrate specificity, including urate and haem, as well as multiple synthetic compounds [275]. The functional transporter is likely to be a homodimer, although higher oligomeric states have also been proposed.	Putative functional dependence on ABCG1	Transports phytosterols and cholesterol; forms an obligate heterodimer with ABCG8. Loss‐of‐function mutations in ABCG5 are associated with sitosterolemia (OMIM: 210250).	Transports phytosterols and cholesterol; forms an obligate heterodimer with ABCG5. Loss‐of‐function mutations in ABCG8 are associated with sitosterolemia (OMIM: 210250).

## F‐type and V‐type ATPases

### Overview

The F‐type (ATP synthase) and the V‐type (vacuolar or vesicular proton pump) ATPases, although having distinct subcellular locations and roles, exhibit marked similarities in subunit structure and mechanism. They are both composed of a ‘soluble’ complex (termed F_1_ or V_1_) and a membrane complex (F_o_ or V_o_). Within each ATPase complex, the two individual sectors appear to function as connected opposing rotary motors, coupling catalysis of ATP synthesis or hydrolysis to proton transport. Both the F‐type and V‐type ATPases have been assigned enzyme commission number E.C. 3.6.3.14

## F‐type ATPase

### Overview

The F‐type ATPase, also known as ATP synthase or ATP phosphohydrolase (H^+^‐transporting), is a mitochondrial membrane‐associated multimeric complex consisting of two domains, an F_0_ channel domain in the membrane and an F_1_ domain extending into the lumen. Proton transport across the inner mitochondrial membrane is used to drive the synthesis of ATP, although it is also possible for the enzyme to function as an ATPase. The ATP5O subunit (oligomycin sensitivity‐conferring protein, OSCP, (P48047)), acts as a connector between F_1_ and F_0_ motors.

The F_1_ motor, responsible for ATP turnover, has the subunit composition *α*3*β*3*γ* *δ* *ε*.

The F_0_ motor, responsible for ion translocation, is complex in mammals, with probably nine subunits centring on A, B, and C subunits in the membrane, together with D, E, F2, F6, G2 and 8 subunits. Multiple pseudogenes for the F_0_ motor proteins have been defined in the human genome.

## V‐type ATPase

### Overview

The V‐type ATPase is most prominently associated with lysosomes in mammals, but also appears to be expressed on the plasma membrane and neuronal synaptic vesicles.

The V_1_ motor, responsible for ATP turnover, has eight subunits with a composition of A‐H.

TheV_0_ motor, responsible for ion translocation, has six subunits (a‐e).

### Further Reading

El Far O et al. (2011) A role for V‐ATPase subunits in synaptic vesicle fusion? J. Neurochem. 117: 603‐12 [PMID:21375531]

Junge W et al. (2009) Torque generation and elastic power transmission in the rotary F(O)F(1)‐ATPase. Nature 459: 364‐70 [PMID:19458712]

Krah A. (2015) Linking structural features from mitochondrial and bacterial F‐type ATP synthases to their distinct mechanisms of ATPase inhibition. Prog. Biophys. Mol. Biol. [PMID:26140992]

Marshansky V et al. (2014) Eukaryotic V‐ATPase: novel structural findings and functional insights. Biochim. Biophys. Acta 1837: 857‐79 [PMID:24508215]

Martínez‐Reyes I et al. (2014) The H(+)‐ATP synthase: a gate to ROS‐mediated cell death or cell survival. Biochim. Biophys. Acta 1837: 1099‐112 [PMID:24685430]

Mindell JA. (2012) Lysosomal acidification mechanisms. Annu. Rev. Physiol. 74: 69‐86 [PMID:22335796]

Nakamoto RK et al. (2008) The rotary mechanism of the ATP synthase. Arch. Biochem. Biophys. 476: 43‐50 [PMID:18515057]

Nakanishi‐Matsui M et al. (2010) The mechanism of rotating proton pumping ATPases. Biochim. Biophys. Acta 1797: 1343‐52 [PMID:20170625]

Okuno D et al. (2011) Rotation and structure of FoF1‐ATP synthase. J. Biochem. 149: 655‐64 [PMID:21524994]

von Ballmoos C et al. (2008) Unique rotary ATP synthase and its biological diversity. Annu Rev Biophys 37: 43‐64 [PMID:18573072]

von Ballmoos C et al. (2009) Essentials for ATP synthesis by F1F0 ATP synthases. Annu. Rev. Biochem. 78: 649‐72 [PMID:19489730]

## P‐type ATPases

### Overview

Phosphorylation‐type ATPases (EC 3.6.3.‐) are associated with membranes and the transport of ions or phospholipids. Characteristics of the family are the transient phosphorylation of the transporters at an aspartate residue and the interconversion between E1 and E2 conformations in the activity cycle of the transporters, taken to represent ‘half‐channels’ facing the cytoplasm and extracellular/luminal side of the membrane, respectively.

Sequence analysis across multiple species allows the definition of five subfamilies, P1‐P5. The P1 subfamily includes heavy metal pumps, such as the copper ATPases. The P2 subfamily includes calcium, sodium/potassium and proton/potassium pumps. The P4 and P5 subfamilies include putative phospholipid flippases.

## Na^+^/K^+^‐ATPases

### Overview

The cell‐surface Na^+^/K^+^‐ATPase is an integral membrane protein which regulates the membrane potential of the cell by maintaining gradients of Na^+^ and K^+^ ions across the plasma membrane, also making a small, direct contribution to membrane potential, particularly in cardiac cells. For every molecule of ATP hydrolysed, the Na^+^/K^+^‐ATPase extrudes three Na^+^ ions and imports two K^+^ ions. The active transporter is a heteromultimer with incompletely defined stoichiometry, possibly as tetramers of heterodimers, each consisting of one of four large, ten TM domain catalytic *α* subunits and one of three smaller, single TM domain glycoprotein *β*‐subunits (see table). Additional protein partners known as FXYD proteins (e.g. FXYD2, P54710) appear to associate with and regulate the activity of the pump.

### Comments

Na^+^/K^+^‐ATPases are inhibited by ouabain and cardiac glycosides, such as digoxin, as well as potentially endogenous cardiotonic steroids [23].

## Ca^2+^‐ATPases

### Overview

The sarcoplasmic/endoplasmic reticulum Ca^2+^‐ATPase (SERCA) is an intracellular membrane‐associated pump for sequestering calcium from the cytosol into intracellular organelles, usually associated with the recovery phase following excitation of muscle and nerves.

The plasma membrane Ca^2+^‐ATPase (PMCA) is a cell‐surface pump for extruding calcium from the cytosol, usually associated with the recovery phase following excitation of cells. The active pump is a homodimer, each subunit of which is made up of ten TM segments, with cytosolic C‐ and N‐termini and two large intracellular loops.

Secretory pathway Ca^2+^‐ATPases (SPCA) allow accumulation of calcium and manganese in the Golgi apparatus.

### Comments

The fungal toxin ochratoxin A has been described to activate SERCA in kidney microsomes [91]. Cyclopiazonic acid [440], thapsigargin[324] and BHQ are widely employed to block SERCA. Thapsigargin has also been described to block the TRPV1 vanilloid receptor [485].

The stoichiometry of flux through the PMCA differs from SERCA, with the PMCA transporting 1 Ca^2+^ while SERCA transports 2 Ca^2+^.

Loss‐of‐function mutations in SPCA1 appear to underlie Hailey‐Hailey disease [234].

## H^+^/K^+^‐ATPases

### Overview

The H^+^/K^+^ ATPase is a heterodimeric protein, made up of *α* and *β* subunits. The *α* subunit has 10 TM domains and exhibits catalytic and pore functions, while the *β* subunit has a single TM domain, which appears to be required for intracellular trafficking and stabilising the *α* subunit. The ATP4A and ATP4B subunits are expressed together, while the ATP12A subunit is suggested to be expressed with the *β*1 (ATP1B1) subunit of the Na^+^/K^+^‐ATPase [383].

### Comments

The gastric H^+^/K^+^‐ATPase is inhibited by proton pump inhibitors used for treating excessive gastric acid secretion, including (R)‐lansoprazole and a metabolite of esomeprazole.

## Cu^+^‐ATPases

### Overview

Copper‐transporting ATPases convey copper ions across cell‐surface and intracellular membranes. They consist of eight TM domains and associate with multiple copper chaperone proteins (e.g. ATOX1, O00244).

## Phospholipid‐transporting ATPases

### Overview

These transporters are thought to translocate the aminophospholipids phosphatidylserine and phosphatidylethanolamine from one side of the phospholipid bilayer to the other to generate asymmetric membranes. They are also proposed to be involved in the generation of vesicles from intracellular and cell‐surface membranes.

### Comments

Loss‐of‐function mutations in ATP8B1 are associated with type I familial intrahepatic cholestasis.

A further series of structurally‐related proteins have been identified in the human genome, with as yet undefined function, including ATP13A1 (Q9HD20), ATP13A2 (Q9NQ11), ATP13A3 (Q9H7F0), ATP13A4 (Q4VNC1) and ATP13A5(Q4VNC0).

### Further Reading

Argüello JM et al. (2012) Metal transport across biomembranes: emerging models for a distinct chemistry. J. Biol. Chem. 287: 13510‐7 [PMID:22389499]

Benarroch EE. (2011) Na+, K+‐ATPase: functions in the nervous system and involvement in neurologic disease. Neurology 76: 287‐93 [PMID:21242497]

Brini M et al. (2009) Calcium pumps in health and disease. Physiol. Rev. 89: 1341‐78 [PMID:19789383]

Bublitz M et al. (2013) Ion pathways in the sarcoplasmic reticulum Ca2+‐ATPase. J. Biol. Chem. 288: 10759‐65 [PMID:23400778]

Bueno‐Orovio A et al. (2014) Na/K pump regulation of cardiac repolarization: insights from a systems biology approach. Pflugers Arch. 466: 183‐93 [PMID:23674099]

Cereijido M et al. (2012) The Na+‐K+‐ATPase as self‐adhesion molecule and hormone receptor. Am. J. Physiol., Cell Physiol. 302: C473‐81 [PMID:22049208]

Contreras‐Ferrat A et al. (2014) Calcium signaling in insulin action on striated muscle. Cell Calcium 56: 390‐6 [PMID:25224502]

Crambert G. (2014) H‐K‐ATPase type 2: relevance for renal physiology and beyond. Am. J. Physiol. Renal Physiol. 306: F693‐700 [PMID:24431203]

Galougahi KK et al. (2012) *β*‐Adrenergic regulation of the cardiac Na+‐K+ ATPase mediated by oxidative signaling. Trends Cardiovasc. Med. 22: 83‐7 [PMID:23040838]

Gupta SP. (2012) Quantitative structure‐activity relationship studies on Na+,K(+)‐ATPase inhibitors. Chem. Rev. 112: 3171‐92 [PMID:22360614]

Inesi G et al. (2014) Biochemical characterization of P‐type copper ATPases. Biochem. J. 463: 167‐76 [PMID:25242165]

Kaler SG. (2011) ATP7A‐related copper transport diseases‐emerging concepts and future trends. Nat Rev Neurol 7: 15‐29 [PMID:21221114]

Lopez‐Marques RL et al. (2014) P4‐ATPases: lipid flippases in cell membranes. Pflugers Arch. 466: 1227‐40 [PMID:24077738]

Lopreiato R et al. (2014) The plasma membrane calcium pump: new ways to look at an old enzyme. J. Biol. Chem. 289: 10261‐8 [PMID:24570005]

López‐Marqués RL et al. (2011) Pumping lipids with P4‐ATPases. Biol. Chem. 392: 67‐76 [PMID:21194369]

Mattle D et al. (2013) On allosteric modulation of P‐type Cu(+)‐ATPases. J. Mol. Biol. 425: 2299‐308 [PMID:23500486]

Michelangeli F et al. (2011) A diversity of SERCA Ca2+ pump inhibitors. Biochem. Soc. Trans. 39: 789‐97 [PMID:21599650]

Morth JP et al. (2011) A structural overview of the plasma membrane Na+,K+‐ATPase and H+‐ATPase ion pumps. Nat. Rev. Mol. Cell Biol. 12: 60‐70 [PMID:21179061]

Palmgren MG et al. (2011) P‐type ATPases. Annu Rev Biophys 40: 243‐66 [PMID:21351879]

Pittman JK. (2011) Vacuolar Ca(2+) uptake. Cell Calcium 50: 139‐46 [PMID:21310481]

Pizzo P et al. (2011) Ca(2+) signalling in the Golgi apparatus. Cell Calcium 50: 184‐92 [PMID:21316101]

Reinhard L et al. (2013) Na(+),K (+)‐ATPase as a docking station: protein‐protein complexes of the Na(+),K (+)‐ATPase. Cell. Mol. Life Sci. 70: 205‐22 [PMID:22695678]

Sebastian TT et al. (2012) Phospholipid flippases: building asymmetric membranes and transport vesicles. Biochim. Biophys. Acta 1821: 1068‐77 [PMID:22234261]

Sikkel MB et al. (2014) SERCA2a gene therapy in heart failure: an anti‐arrhythmic positive inotrope. Br. J. Pharmacol. 171: 38‐54 [PMID:24138023]

Strehler EE. (2015) Plasma membrane calcium ATPases: From generic Ca(2+) sump pumps to versatile systems for fine‐tuning cellular Ca(2.). Biochem. Biophys. Res. Commun. 460: 26‐33 [PMID:25998731]

## Major facilitator superfamily (MFS) of transporters

**Table:** 

Nomenclature	synaptic vesicle glycoprotein 2A
HGNC, UniProt	SV2A, Q7L0J3
Inhibitors	levetiracetam (p*K*_i_ 5.8) [363] – Rat

## SLC superfamily of solute carriers

### Overview

The SLC superfamily of solute carriers is the second largest family of membrane proteins after G protein‐coupled receptors, but with a great deal fewer therapeutic drugs that exploit them. As with the ABC transporters, however, they play a major role in drug disposition and so can be hugely influential in determining the clinical efficacy of particular drugs.

48 families are identified on the basis of sequence similarities, but many of them overlap in terms of the solutes that they carry. For example, amino acid accumulation is mediated by members of the SLC1, SLC3/7, SLC6, SLC15, SLC16, SLC17, SLC32, SLC36, SLC38 and SLC43. Further members of the SLC superfamily regulate ion fluxes at the plasma membrane, or solute transport into and out of cellular organelles.

Within the SLC superfamily, there is an abundance in diversity of structure. Two families (SLC3 and SLC7) only generate functional transporters as heteromeric partners, where one partner is a single TM domain protein. Membrane topology predictions for other families suggest 3, 4 6, 7, 8, 9, 10, 11, 12, 13, or 14 TM domains. Functionally, members may be divided into those dependent on gradients of ions (particularly sodium, chloride or protons), exchange of solutes or simple equilibrative gating. For many members, the stoichiometry of transport is not yet established. Furthermore, one family of transporters also possess enzymatic activity (SLC27), while many members function as ion channels (e.g. SLC1A7/EAAT5), which increases the complexity of function of the SLC superfamily.

## SLC1 family of amino acid transporters

### Overview

The SLC1 family of sodium dependent transporters includes the plasma membrane located glutamate transporters and the neutral amino acid transporters ASCT1 and ASCT2 [7, 32, 264, 265, 378].

## Glutamate transporter subfamily

### Overview

Glutamate transporters present the unusual structural motif of 8TM segments and 2 re‐entrant loops [208]. The crystal structure of a glutamate transporter homologue (GltPh) from Pyrococcus horikoshii supports this topology and indicates that the transporter assembles as a trimer, where each monomer is a functional unit capable of substrate permeation [53, 406, 533] reviewed by [254]). This structural data is in agreement with the proposed quaternary structure for EAAT2 [185] and several functional studies that propose the monomer is the functional unit [205, 284, 302, 418]. Recent evidence suggests that EAAT3 and EAAT4 may assemble as heterotrimers [362]. The activity of glutamate transporters located upon both neurones (predominantly EAAT3, 4 and 5) and glia (predominantly EAAT 1 and 2) serves, dependent upon their location, to regulate excitatory neurotransmission, maintain low ambient extracellular concentrations of glutamate (protecting against excitotoxicity) and provide glutamate for metabolism including the glutamate‐glutamine cycle. The Na^+^/K^+^‐ATPase that maintains the ion gradients that drive transport has been demonstrated to co‐assemble with EAAT1 and EAAT2 [412]. Recent evidence supports altered glutamate transport and novel roles in brain for splice variants of EAAT1 and EAAT2 [184, 303]. Three patients with dicarboxylic aminoaciduria (DA) were recently found to have loss‐of‐function mutations in EAAT3 [24]. DA is characterized by excessive excretion of the acidic amino acids glutamate and aspartate and EAAT3 is the predominant glutamate/aspartate transporter in the kidney. Enhanced expression of EAAT2 resulting from administration of *β*‐lactam antibiotics (e.g. ceftriaxone) is neuroprotective and occurs through NF‐*κ*B‐mediated EAAT2 promoter activation [181, 306, 414] reviewed by [277]). PPAR*γ* activation (e.g. by rosiglitazone) also leads to enhanced expression of EAAT though promoter activation [411]. In addition, several translational activators of EAAT2 have recently been described [98] along with treatments that increase the surface expression of EAAT2 (e.g. [301, 554]), or prevent its down‐regulation (e.g. [199]). A thermodynamically uncoupled Cl^‐^ flux, activated by Na^+^ and glutamate [207, 265, 327] (Na^+^ and aspartate in the case of GltPh [417]), is sufficiently large, in the instances of EAAT4 and EAAT5, to influence neuronal excitability [476, 498]. Indeed, it has recently been suggested that the primary function of EAAT5 is as a slow anion channel gated by glutamate, rather than a glutamate transporter [176].

**Table:** 

Nomenclature	Excitatory amino acid	Excitatory amino acid	Excitatory amino acid	Excitatory amino acid	Excitatory amino acid
	transporter 1	transporter 2	transporter 3	transporter 4	transporter 5
Systematic nomenclature	SLC1A3	SLC1A2	SLC1A1	SLC1A6	SLC1A7
Common abreviation	EAAT1	EAAT2	EAAT3	EAAT4	EAAT5
HGNC, UniProt	SLC1A3, P43003	SLC1A2, P43004	SLC1A1, P43005	SLC1A6, P48664	SLC1A7, O00341
Substrates	DL‐threo‐*β*‐hydroxyaspartate (*K*_i_5.8 × 10^−5^M) [444], D‐aspartic acid, L‐trans‐2, 4‐pyrolidine dicarboxylate	D‐aspartic acid, DL‐threo‐*β*‐hydroxyaspartate, L‐trans‐2, 4‐pyrolidine dicarboxylate [285]	L‐trans‐2, 4‐pyrolidine dicarboxylate, DL‐threo‐*β*‐hydroxyaspartate, D‐aspartic acid	D‐aspartic acid, DL‐threo‐*β*‐hydroxyaspartate, L‐trans‐2, 4‐pyrolidine dicarboxylate	D‐aspartic acid, L‐trans‐2, 4‐pyrolidine dicarboxylate, DL‐threo‐*β*‐hydroxyaspartate
Endogenous substrates	L‐aspartic acid, L‐glutamic acid	L‐glutamic acid, L‐aspartic acid	L‐aspartic acid, L‐cysteine [543], L‐glutamic acid	L‐glutamic acid, L‐aspartic acid	L‐aspartic acid, L‐glutamic acid
Stoichiometry	Probably 3 Na^+^: 1 H^+^ : 1 glutamate (in): 1 K^+^ (out)	3 Na^+^: 1 H^+^ : 1 glutamate (in): 1 K^+^ (out) [311]	3 Na^+^: 1 H^+^ : 1 glutamate (in): 1 K^+^ (out) [542]	Probably 3 Na^+^: 1 H^+^ : 1 glutamate (in): 1 K^+^(out)	Probably 3 Na^+^: 1 H^+^ : 1 glutamate (in): 1 K^+^ (out)
Inhibitors	UCPH‐101 (membrane potential assay) (pIC_50_ 6.9) [252], DL‐TBOA (p*K*_B_ 5) [444]	WAY‐213613 (pIC_50_ 7.1) [133], DL‐TBOA (p*K*_B_ 6.9) [444], SYM2081 (p*K*_B_ 5.5) [492], dihydrokainate (p*K*_B_ 5), threo‐3‐methylglutamate (p*K*_B_ 4.7) [492]	NBI‐59159 (pIC_50_ 7.1) [131], L‐*β*‐BA ([^3^H]D‐aspartate uptake assay) (p*K*_i_ 6.1) [150], DL‐TBOA (pIC_50_ 5.1) [446]	DL‐TBOA (p*K*_i_ 5.4) [443], threo‐3‐methylglutamate (p*K*_i_ 4.3) [141]	DL‐TBOA (p*K*_i_ 5.5) [443]
Labelled ligands	[^3^H]ETB‐TBOA (Binding) (p*K*_d_ 7.8) [445] – Rat, [^3^H](2S,4R)‐4‐methylglutamate, [^3^H]D‐aspartic acid, [^3^H]L‐aspartic acid	[^3^H]ETB‐TBOA (Binding) (p*K*_d_ 7.8) [445] – Rat, [^3^H](2S,4R)‐4‐methylglutamate, [^3^H]D‐aspartic acid, [^3^H]L‐aspartic acid	[^3^H]ETB‐TBOA (Binding) (p*K*_d_ 6.5) [445] – Rat, [^3^H]D‐aspartic acid, [^3^H]L‐aspartic acid	[^3^H]ETB‐TBOA (Binding) (p*K*_d_ 7.9) [445] – Rat, [^3^H]D‐aspartic acid, [^3^H]L‐aspartic acid	[^3^H]ETB‐TBOA (Binding) (p*K*_d_ 7.6) [445] – Rat, [^3^H]D‐aspartic acid, [^3^H]L‐aspartic acid

### Comments

The K_B_ (or K_i_) values reported, unless indicated otherwise, are derived from transporter currents mediated by EAATs expressed in voltage‐clamped Xenopus laevis oocytes [141, 443, 444, 492]. K_B_ (or K_i_) values derived in uptake assays are generally higher (e.g. [444]). In addition to acting as a poorly transportable inhibitor of EAAT2, (2S,4R)‐4‐methylglutamate, also known as SYM2081, is a competitive substrate for EAAT1 (K_M_= 54*μ*M; [235, 492]) and additionally is a potent kainate receptor agonist [548] which renders the compound unsuitable for autoradiographic localisation of EAATs [14]. Similarly, at concentrations that inhibit EAAT2, dihydrokainate binds to kainate receptors [444]. WAY‐855 and WAY‐213613 are both non‐substrate inhibitors with a preference for EAAT2 over EAAT3 and EAAT1 [132, 133]. NBI‐59159 is a non‐substrate inhibitor with modest selectivity for EAAT3 over EAAT1 (>10‐fold) and EAAT2 (5‐fold) [99, 130]. Analogously, L‐*β*‐threo‐benzyl‐aspartate (L‐*β*‐BA) is a competitive non‐substrate inhibitor that preferentially blocks EAAT3 versus EAAT1, or EAAT2 [150]. [^3^H](2S,4R)‐4‐methylglutamate demonstrates low affinity binding (K_D_≅ 6.0 *μ*M) to EAAT1 and EAAT2 in rat brain homogenates [15] and EAAT1 in murine astrocyte membranes [13], whereas [^3^H]ETB‐TBOA binds with high affinity to all EAATs other than EAAT3 [445]. The novel isoxazole derivative (‐)‐HIP‐A may interact at the same site as TBOA and preferentially inhibit reverse transport of glutamate [97]. Threo‐3‐methylglutamate induces substrate‐like currents at EAAT4, but does not elicit heteroexchange of [^3^H]‐aspartate in synaptosome preparations, inconsistent with the behaviour of a substrate inhibitor [141]. Parawixin 1, a compound isolated from the venom from the spider Parawixia bistriata is a selective enhancer of the glutamate uptake through EAAT2 but not through EAAT1 or EAAT3 [165, 166]. In addition to the agents listed in the table, DL‐threo‐*β*‐hydroxyaspartate and L‐trans‐2,4‐pyrolidine dicarboxylate act as non‐selective competitive substrate inhibitors of all EAATs. Zn^2+^ and arachidonic acid are putative endogenous modulators of EAATs with actions that differ across transporter subtypes (reviewed by [491]).

## Alanine/serine/cysteine transporter subfamily

### Overview

ASC transporters mediate Na^+^‐dependent exchange of small neutral amino acids such as Ala, Ser, Cys and Thr and their structure is predicted to be similar to that of the glutamate transporters [16, 489]. ASCT1 and ASCT2 also exhibit thermodynamically uncoupled chloride channel activity associated with substrate transport [63, 541]. Whereas EAATs counter‐transport K^+^ (see above) ASCTs do not and their function is independent of the intracellular concentration of K^+^[541].

**Table:** 

Nomenclature	Alanine/serine/cysteine transporter 1	Alanine/serine/cysteine transporter 2
Systematic nomenclature	SLC1A4	SLC1A5
Common abreviation	ASCT1	ASCT2
HGNC, UniProt	SLC1A4, P43007	SLC1A5, Q15758
Endogenous substrates	L‐cysteine>L‐alanine = L‐serine>L‐threonine	L‐alanine = L‐serine = L‐cysteine (low Vmax) = L‐threonine = L‐glutamine = L‐asparagine≫L‐methionine≅glycine≅L‐leucine>L‐valine>L‐glutamic acid (enhanced at low pH)
Stoichiometry	1 Na^+^: 1 amino acid (in): 1 Na^+^: 1 amino acid (out); (homo‐, or hetero‐exchange; [542])	1 Na^+^: 1 amino acid (in): 1 Na^+^: 1 amino acid (out); (homo‐, or hetero‐exchange; [61])
Inhibitors	–	p‐nitrophenyl glutamyl anilide (p*K*_i_ 4.3) [151] – Rat, benzylcysteine (p*K*_i_ 3.1) [206], benzylserine (p*K*_i_ 3) [206]

### Comments

The substrate specificity of ASCT1 may extend to L‐proline and trans‐4‐hydroxy‐proline[386]. At low pH ( 5.5) both ASCT1 and ASCT2 are able to exchange acidic amino acids such as L‐cysteate and glutamate [466, 489]. In addition to the inhibitors tabulated above, HgCl_2_, methylmercury and mersalyl, at low micromolar concentrations, non‐competitively inhibit ASCT2 by covalent modificiation of cysteine residues [372].

### Further Reading

Bröer S et al. (2011) The role of amino acid transporters in inherited and acquired diseases. Biochem. J. 436: 193‐211 [PMID:21568940]

Chao XD et al. (2010) The role of excitatory amino acid transporters in cerebral ischemia. Neurochem. Res. 35: 1224‐30 [PMID:20440555]

Grewer C et al. (2014) SLC1 glutamate transporters. Pflugers Arch. 466: 3‐24 [PMID:24240778]

Jiang J et al. (2011) New views of glutamate transporter structure and function: advances and challenges. Neuropharmacology 60: 172‐81 [PMID:20708631]

Kanai Y et al. (2013) The SLC1 high‐affinity glutamate and neutral amino acid transporter family. Mol. Aspects Med. 34: 108‐20 [PMID:23506861]

Kim K et al. (2011) Role of excitatory amino acid transporter‐2 (EAAT2) and glutamate in neurodegeneration: opportunities for developing novel therapeutics. J. Cell. Physiol. 226: 2484‐93 [PMID:21792905]

## SLC2 family of hexose and sugar alcohol transporters

### Overview

The SLC2 family transports D‐glucose, D‐fructose, inositol (e.g. myo‐inositol) and related hexoses. Three classes of glucose transporter can be identified, separating GLUT1‐4 and 14, GLUT6, 8, 10 and 12; and GLUT5, 7, 9 and 11. Modelling suggests a 12 TM membrane topology, with intracellular termini, with functional transporters acting as homodimers or homotetramers.

## Class I transporters

### Overview

Class I transporters are able to transport D‐glucose, but not D‐fructose, in the direction of the concentration gradient and may be inhibited non‐selectively by phloretin and cytochalasin B. GLUT1 is the major glucose transporter in brain, placenta and erythrocytes, GLUT2 is found in the pancreas, liver and kidneys, GLUT3 is neuronal and placental, while GLUT4 is the insulin‐responsive transporter found in skeletal muscle, heart and adipose tissue. GLUT14 appears to result from gene duplication of GLUT3 and is expressed in the testes [521].

**Table:** 

Nomenclature	Glucose transporter 1	Glucose transporter 2	Glucose transporter 3	Glucose transporter 4	Glucose transporter 14
Systematic nomenclature	SLC2A1	SLC2A2	SLC2A3	SLC2A4	SLC2A14
Common abreviation	GLUT1	GLUT2	GLUT3	GLUT4	GLUT14
HGNC, UniProt	SLC2A1, P11166	SLC2A2, P11168	SLC2A3, P11169	SLC2A4, P14672	SLC2A14, Q8TDB8
Substrates	D‐glucosamine (D‐glucose = D‐glucosamine) [487], dehydroascorbic acid [39], D‐glucose (D‐glucose = D‐glucosamine) [487]	D‐glucosamine (D‐glucosamine > D‐glucose) [487], D‐glucose (D‐glucosamine > D‐glucose) [487]	D‐glucose	D‐glucosamine (D‐glucosamine ≥ D‐glucose) [487], D‐glucose (D‐glucosamine ≥ D‐glucose) [487]	–
Labelled ligands	[^3^H]2‐deoxyglucose	[^3^H]2‐deoxyglucose	[^3^H]2‐deoxyglucose	[^3^H]2‐deoxyglucose	–

## Class II transporters

### Overview

Class II transporters transport D‐fructose and appear to be insensitive to cytochalasin B. Class II transporters appear to be predominantly intracellularly located.

**Table:** 

Nomenclature	Glucose transporter 5	Glucose transporter 7	Glucose transporter 9	Glucose transporter 11
Systematic nomenclature	SLC2A5	SLC2A7	SLC2A9	SLC2A11
Common abreviation	GLUT5	GLUT7	GLUT9	GLUT11
HGNC, UniProt	SLC2A5, P22732	SLC2A7, Q6PXP3	SLC2A9, Q9NRM0	SLC2A11, Q9BYW1
Substrates	D‐fructose (D‐fructose > D‐glucose) [67], D‐glucose (D‐fructose > D‐glucose) [67]	D‐fructose [81], D‐glucose [81]	D‐fructose [75], uric acid [75]	D‐fructose [332], D‐glucose [122]

**Table:** 

Nomenclature	Glucose transporter 6	Glucose transporter 8	Glucose transporter 10	Glucose transporter 12
Systematic nomenclature	SLC2A6	SLC2A8	SLC2A10	SLC2A12
Common abreviation	GLUT6	GLUT8	GLUT10	GLUT12
HGNC, UniProt	SLC2A6, Q9UGQ3	SLC2A8, Q9NY64	SLC2A10, O95528	SLC2A12, Q8TD20
Substrates	–	D‐glucose [238]	dehydroascorbic acid [308], D‐glucose [308]	D‐glucose [409]

## Proton‐coupled inositol transporter

### Overview

Proton‐coupled inositol transporters are expressed predominantly in the brain and can be inhibited by phloretin and cytochalasin B[487].

**Table:** 

Nomenclature	Proton myo‐inositol cotransporter
Systematic nomenclature	SLC2A13
Common abreviation	HMIT
HGNC, UniProt	SLC2A13, Q96QE2
Substrates	D‐chiro‐inositol [487], myo‐inositol [487], scyllo‐inositol [487], muco‐inositol [487]
Stoichiometry	1 H^+^ : 1 inositol (in) [118]

### Further Reading

Augustin R. (2010) The protein family of glucose transport facilitators: It's not only about glucose after all. IUBMB Life 62: 315‐33 [PMID:20209635]

Klip A et al. (2014) Signal transduction meets vesicle traffic: the software and hardware of GLUT4 translocation. Am. J. Physiol., Cell Physiol. 306: C879‐86 [PMID:24598362]

Leney SE et al. (2009) The molecular basis of insulin‐stimulated glucose uptake: signalling, trafficking and potential drug targets. J. Endocrinol. 203: 1‐18 [PMID:19389739]

Mueckler M et al. (2013) The SLC2 (GLUT) family of membrane transporters. Mol. Aspects Med. 34: 121‐38 [PMID:23506862]

Uldry M et al. (2004) The SLC2 family of facilitated hexose and polyol transporters. Pflugers Arch. 447: 480‐9 [PMID:12750891]

Wolking S et al. (2014) Focal epilepsy in glucose transporter type 1 (Glut1) defects: case reports and a review of literature. J. Neurol. 261: 1881‐6 [PMID:25022942]

## SLC3 and SLC7 families of heteromeric amino acid transporters (HATs)

### Overview

The SLC3 and SLC7 families combine to generate functional transporters, where the subunit composition is a disulphide‐linked combination of a heavy chain (SLC3 family) with a light chain (SLC7 family).

## SLC3 family

### Overview

SLC3 family members are single TM proteins with extensive glycosylation of the exterior C‐terminus, which heterodimerize with SLC7 family members in the endoplasmic reticulum and assist in the plasma membrane localization of the transporter.

## SLC7 family

### Overview

SLC7 family members may be divided into two major groups: cationic amino acid transporters (CATs) and glycoprotein‐associated amino acid transporters (gpaATs).

Cationic amino acid transporters are 14 TM proteins, which mediate pH‐ and sodium‐independent transport of cationic amino acids (system y^+^), apparently as an exchange mechanism. These transporters are sensitive to inhibition by N‐ethylmaleimide.

**Table:** 

Nomenclature	High affinity cationic amino acid	Low affinity cationic amino acid	Cationic amino acid	L‐type amino acid transporter 1
	transporter 1	transporter 2	transporter 3	
Systematic nomenclature	SLC7A1	SLC7A2	SLC7A3	SLC7A5
Common abreviation	CAT1	CAT2	CAT3	LAT1
HGNC, UniProt	SLC7A1, P30825	SLC7A2, P52569	SLC7A3, Q8WY07	SLC7A5, Q01650
Substrates	L‐ornithine, L‐arginine, L‐lysine, L‐histidine	L‐ornithine, L‐arginine, L‐lysine, L‐histidine	L‐ornithine, L‐arginine, L‐lysine	–

**Table:** 

Nomenclature	L‐type amino acid transporter 2	y+L amino acid transporter 1	y+L amino acid transporter 2	b^0,+^‐type amino acid transporter 1
Systematic nomenclature	SLC7A8	SLC7A7	SLC7A6	SLC7A9
Common abreviation	LAT2	y+LAT1	y+LAT2	b^0,+^AT
HGNC, UniProt	SLC7A8, Q9UHI5	SLC7A7, Q9UM01	SLC7A6, Q92536	SLC7A9, P82251

**Table:** 

Nomenclature	Asc‐type amino acid transporter 1	Cystine/glutamate transporter	AGT1
Systematic nomenclature	SLC7A10	SLC7A11	SLC7A13
Common abreviation	Asc‐1	xCT	–
HGNC, UniProt	SLC7A10, Q9NS82	SLC7A11, Q9UPY5	SLC7A13, Q8TCU3

### Comments

CAT4 appears to be non‐functional in heterologous expression [516], while SLC7A14 has yet to be characterized.

Glycoprotein‐associated amino acid transporters are 12 TM proteins, which heterodimerize with members of the SLC3 family to act as cell‐surface amino acid exchangers.

Heterodimers between 4F2hc and LAT1 or LAT2 generate sodium‐independent system L transporters. LAT1 transports large neutral amino acids including branched‐chain and aromatic amino acids as well as miglustat, whereas LAT2 transports most of the neutral amino acids.

Heterodimers between 4F2hc and y^+^LAT1 or y^+^LAT2 generate transporters similar to the system y^+^L , which transport cationic (L‐arginine, L‐lysine, L‐ornithine) amino acids independent of sodium and neutral (L‐leucine, L‐isoleucine, L‐methionine, L‐glutamine) amino acids in a partially sodium‐dependent manner. These transporters are N‐ethylmaleimide‐insensitive. Heterodimers between rBAT and b^0,+^AT appear to mediate sodium‐independent system b^0,+^ transport of most of the neutral amino acids and cationic amino acids (L‐arginine, L‐lysine and L‐ornithine).

Asc‐1 appears to heterodimerize with 4F2hc to allow the transport of small neutral amino acids (such as L‐alanine, L‐serine, L‐threonine, L‐glutamine and glycine), as well as D‐serine, in a sodium‐independent manner.

xCT generates a heterodimer with 4F2hc for a system x^‐^_e‐c_ transporter that mediates the sodium‐independent exchange of L‐cystine and L‐glutamic acid.

AGT has been conjugated with SLC3 members as fusion proteins to generate functional transporters, but the identity of a native heterodimer has yet to be ascertained.

### Further Reading

Bhutia YD et al. (2015) Amino Acid transporters in cancer and their relevance to ~glutamine addiction~: novel targets for the design of a new class of anticancer drugs. Cancer Res. 75: 1782‐8 [PMID:25855379]

Closs EI et al. (2006) Structure and function of cationic amino acid transporters (CATs). J. Membr. Biol. 213: 67‐77 [PMID:17417706]

Fotiadis D et al. (2013) The SLC3 and SLC7 families of amino acid transporters. Mol. Aspects Med. 34: 139‐58 [PMID:23506863]

Palacín M et al. (2004) The ancillary proteins of HATs: SLC3 family of amino acid transporters. Pflugers Arch. 447: 490‐4 [PMID:14770309]

Palacín M et al. (2005) The genetics of heteromeric amino acid transporters. Physiology (Bethesda) 20: 112‐24 [PMID:15772300]

Verrey F et al. (2004) CATs and HATs: the SLC7 family of amino acid transporters. Pflugers Arch. 447: 532‐42 [PMID:14770310]

## SLC4 family of bicarbonate transporters

### Overview

Together with the SLC26 family, the SLC4 family of transporters subserve anion exchange, principally of chloride and bicarbonate (HCO_3_^‐^), but also carbonate and hydrogen sulphate (HSO_4_^‐^). SLC4 family members regulate bicarbonate fluxes as part of carbon dioxide movement, chyme neutralization and reabsorption in the kidney.

Within the family, subgroups of transporters are identifiable: the electroneutral sodium‐independent Cl^‐^/HCO_3_^‐^ transporters (AE1, AE2 and AE3), the electrogenic sodium‐dependent HCO_3_^‐^ transporters (NBCe1 and NBCe2) and the electroneutral HCO_3_^‐^ transporters (NBCn1 and NBCn2). Topographical information derives mainly from study of AE1, abundant in erythrocytes, which suggests a dimeric or tetrameric arrangement, with subunits made up of 13 TM domains and re‐entrant loops at TM9/10 and TM11/12. The N terminus exhibits sites for interaction with multiple proteins, including glycolytic enzymes, haemoglobin and cytoskeletal elements.

## Anion exchangers

**Table:** 

Nomenclature	Anion exchange protein 1	Anion exchange protein 2	Anion exchange protein 3	Anion exchange protein 4
Systematic nomenclature	SLC4A1	SLC4A2	SLC4A3	SLC4A9
Common abreviation	AE1	AE2	AE3	AE4
HGNC, UniProt	SLC4A1, P02730	SLC4A2, P04920	SLC4A3, P48751	SLC4A9, Q96Q91
Endogenous substrates	HCO_3_^‐^, Cl^‐^	Cl^‐^, HCO_3_^‐^	Cl^‐^, HCO_3_^‐^	–
Stoichiometry	1 Cl^‐^ (in) : 1 HCO_3_^‐^ (out)	1 Cl^‐^ (in) : 1 HCO_3_^‐^ (out)	1 Cl^‐^ (in) : 1 HCO_3_^‐^ (out)	–

## Sodium‐dependent HCO ${}_{\text{3}}^{\text{‐}}$ transporters

**Table:** 

Nomenclature	Electrogenic sodium bicarbonate	Electrogenic sodium bicarbonate	Electroneutral sodium bicarbonate
	cotransporter 1	cotransporter 4	cotransporter 1
Systematic nomenclature	SLC4A4	SLC4A5	SLC4A7
Common abreviation	NBCe1	NBCe2	NBCn1
HGNC, UniProt	SLC4A4, Q9Y6R1	SLC4A5, Q9BY07	SLC4A7, Q9Y6M7
Endogenous substrates	NaHCO_3_^‐^	NaHCO_3_^‐^	NaHCO_3_^‐^
Stoichiometry	1 Na^+^ : 2/3 HCO_3_^‐^ (out) or 1 Na^+^ : CO_3_^2*^	1 Na^+^ : 2/3 HCO_3_^‐^ (out) or 1 Na^+^ : CO_3_^2*^	1 Na^+^ : 1 HCO_3_^‐^ (out) or 1 Na^+^ : CO_3_^2*^

**Table:** 

Nomenclature	Electroneutral sodium bicarbonate	NBCBE	NaBC1
	cotransporter 2		
Systematic nomenclature	SLC4A10	SLC4A8	SLC4A11
Common abreviation	NBCn2	NDCBE	BTR1
HGNC, UniProt	SLC4A10, Q6U841	SLC4A8, Q2Y0W8	SLC4A11, Q8NBS3
Endogenous substrates	NaHCO_3_^‐^	NaHCO_3_^‐^, Cl^‐^	Cl^‐^, NaHCO_3_^‐^
Stoichiometry	1 Na^+^ : 1 HCO_3_^‐^ (out) or 1 Na : CO_3_^2*^	1 Na^+^ : 2HCO_3_^‐^ (in) : 1 Cl^‐^ (out)	–

### Further Reading

Alper SL. (2009) Molecular physiology and genetics of Na+‐independent SLC4 anion exchangers. J. Exp. Biol. 212: 1672‐83 [PMID:19448077]

Boron WF et al. (2009) Modular structure of sodium‐coupled bicarbonate transporters. J. Exp. Biol. 212: 1697‐706 [PMID:19448079]

Christensen HL et al. (2013) Na(+) dependent acid‐base transporters in the choroid plexus; insights from slc4 and slc9 gene deletion studies. Front Physiol 4: 304 [PMID:24155723]

Kurtz I. (2014) NBCe1 as a model carrier for understanding the structure‐function properties of Na^+^‐coupled SLC4 transporters in health and disease. Pflugers Arch. 466: 1501‐16 [PMID:24515290]

Liu Y et al. (2012) The physiology of bicarbonate transporters in mammalian reproduction. Biol. Reprod. 86: 99 [PMID:22262691]

Majumdar D et al. (2010) Na‐coupled bicarbonate transporters of the solute carrier 4 family in the nervous system: function, localization, and relevance to neurologic function. Neuroscience 171: 951‐72 [PMID:20884330]

Parker MD et al. (2013) The divergence, actions, roles, and relatives of sodium‐coupled bicarbonate transporters. Physiol. Rev. 93: 803‐959 [PMID:23589833]

Romero MF et al. (2013) The SLC4 family of bicarbonate (HCO_3^−^) transporters. Mol. Aspects Med. 34: 159‐82 [PMID:23506864]

Ruffin VA et al. (2014) Intracellular pH regulation by acid‐base transporters in mammalian neurons. Front Physiol 5: 43 [PMID:24592239]

Thornell IM et al. (2015) Regulators of Slc4 bicarbonate transporter activity. Front Physiol 6: 166 [PMID:26124722]

## SLC5 family of sodium‐dependent glucose transporters

### Overview

The SLC5 family of sodium‐dependent glucose transporters includes, in mammals, the Na^+^/substrate co‐transporters for glucose (e.g. choline), D‐glucose, monocarboxylates, myo‐inositol and I^‐^[159, 179, 518, 519]. Members of the SLC5 and SLC6 families, along with other unrelated Na^+^ cotransporters (i.e. Mhp1 and BetP), share a common structural core that contains an inverted repeat of 5TM *α*‐helical domains [2].

## Hexose transporter family

### Overview

Detailed characterisation of members of the hexose transporter family is limited to SGLT1, 2 and 3, which are all inhibited in a competitive manner by phlorizin, a natural dihydrocholine glucoside, that exhibits modest selectivity towards SGLT2 (see [518] for an extensive review). SGLT1 is predominantly expressed in the small intestine, mediating the absorption of glucose (e.g. D‐glucose), but also occurs in the brain, heart and in the late proximal straight tubule of the kidney. The expression of SGLT2 is almost exclusively restricted to the early proximal convoluted tubule of the kidney, where it is largely responsible for the renal reabsorption of glucose. SGLT3 is not a transporter but instead acts as a glucosensor generating an inwardly directed flux of Na^+^ that causes membrane depolarization [120].

**Table:** 

Nomenclature	Sodium/glucose	Sodium/glucose	Low affinity sodium‐	Sodium/glucose	Sodium/glucose
	cotransporter 1	cotransporter 2	glucose cotransporter	cotransporter 4	cotransporter 5
Systematic nomenclature	SLC5A1	SLC5A2	SLC5A4	SLC5A9	SLC5A10
Common abreviation	SGLT1	SGLT2	SGLT3	SGLT4	SGLT5
HGNC, UniProt	SLC5A1, P13866	SLC5A2, P31639	SLC5A4, Q9NY91	SLC5A9, Q2M3M2	SLC5A10, A0PJK1
Substrates	D‐galactose [501], *α*‐MDG [501], D‐glucose [501]	*α*‐MDG, D‐glucose	D‐glucose [501], 1‐deoxynojirimycin‐1‐sulfonic acid [501], N‐ethyl‐1‐deoxynojirimycin [501], miglustat [501], miglitol [501], 1‐deoxynojirimycin [501]	D‐glucose, D‐mannose, *α*‐MDG	D‐galactose, D‐glucose
Stoichiometry	2 Na^+^ : 1 glucose [266]	1 Na^+^ : 1 glucose [236]	–	–	–
Inhibitors	sotagliflozin (pIC_50_ 7.4) [538], dapagliflozin (pIC_50_ 6.4) [313], canagliflozin (pIC_50_ 6.2) [316], remogliflozin (p*K*_i_ 5.3) [172], empagliflozin (pIC_50_ 5.1) [204], sergliflozin (p*K*_i_ 5.1) [272]	empagliflozin (pIC_50_ 8.5) [204], canagliflozin (pIC_50_ 8.4) [316], remogliflozin (p*K*_i_ 7.9) [172], sergliflozin (p*K*_i_ 6.8) [272]	–	–	–
Selective inhibitors	–	dapagliflozin (pIC_50_ 9.3) [269]	–	–	–
Comments	–	–	'sodium/glucose cotransporter 3' is a misnomer since SGLT3 is a glucosensor.	–	–

### Comments

Recognition and transport of substrate by SGLTs requires that the sugar is a pyranose. De‐oxyglucose derivatives have reduced affinity for SGLT1, but the replacement of the sugar equatorial hydroxyl group by fluorine at some positions, excepting C2 and C3, is tolerated (see [518] for a detailed quantification). Although SGLT1 and SGLT2 have been described as high‐ and low‐affinity sodium glucose co‐transporters, respectively, recent work suggests that they have a similar affinity for glucose under physiological conditions [236]. Selective blockers of SGLT2, and thus blocking  50% of renal glucose reabsorption, are in development for the treatment of diabetes (e.g. [80]).

## Choline transporter

### Overview

The high affinity, hemicholinium‐3‐sensitive, choline transporter (CHT) is expressed mainly in cholinergic neurones on nerve cell terminals and synaptic vesicles (keratinocytes being an additional location). In autonomic neurones, expression of CHT requires an activity‐dependent retrograde signal from postsynaptic neurones [291]. Through recapture of choline generated by the hydrolysis of ACh by acetylcholinesterase, CHT serves to maintain acetylcholine synthesis within the presynaptic terminal [159]. Homozygous mice engineered to lack CHT die within one hour of birth as a result of hypoxia arising from failure of transmission at the neuromuscular junction of the skeletal muscles that support respiration [158]. A low affinity choline uptake mechanism that remains to be identified at the molecular level may involve multiple transporters. In addition, a family of choline transporter‐like (CTL) proteins, (which are members of the SLC44 family) with weak Na^+^ dependence have been described [477].

**Table:** 

Nomenclature	CHT
Systematic nomenclature	SLC5A7
HGNC, UniProt	SLC5A7, Q9GZV3
Substrates	triethylcholine
Endogenous substrates	choline
Stoichiometry	Na^+^ : choline (variable stoichimetry); modulated by extracellular Cl^‐^ [251]
Selective inhibitors	hemicholinium‐3 (p*K*_i_ 7–8) [370]
Labelled ligands	[^3^H]hemicholinium‐3 (p*K*_d_ 8.2–8.4)

### Comments

K_i_ and K_D_ values for hemicholinium‐3 listed in the table are for human CHT expressed in Xenopus laevis oocytes [371], or COS‐7 cells [12]. Hemicholinium mustard is a substrate for CHT that causes covalent modification and irreversible inactivation of the transporter. Several exogenous substances (e.g. triethylcholine) that are substrates for CHT act as precursors to cholinergic false transmitters.

## Sodium iodide symporter, sodium‐dependent multivitamin transporter and sodium‐coupled monocarboxylate transporters

### Overview

The sodium‐iodide symporter (NIS) is an iodide transporter found principally in the thyroid gland where it mediates the accumulation of I^‐^ within thyrocytes. Transport of I^‐^ by NIS from the blood across the basolateral membrane followed by apical efflux into the colloidal lumen, mediated at least in part by pendrin (SLC22A4), and most likely not SMCT1 (SLC5A8) as once thought, provides the I^‐^ required for the synthesis of the thyroid hormones triiodothyronine (triiodothyronine) and thyroxine (T_4_) [42]. NIS is also expressed in the salivary glands, gastric mucosa, intestinal enterocytes and lactating breast. NIS mediates I^‐^ absorption in the intestine and I^‐^ secretion into the milk. SMVT is expressed on the apical membrane of intestinal enterocytes and colonocytes and is the main system responsible for biotin(vitamin H) and pantothenic acid (vitamin B_5_) uptake in humans [422]. SMVT located in kidney proximal tubule epithelial cells mediates the reabsorption of biotin and pantothenic acid. SMCT1 (SLC5A8), which transports a wide range of monocarboxylates, is expressed in the apical membrane of epithelia of the small intestine, colon, kidney, brain neurones and the retinal pigment epithelium [179]. SMCT2 (SLC5A12) also localises to the apical membrane of kidney, intestine, and colon, but in the brain and retina is restricted to astrocytes and Müller cells, respectively [179]. SMCT1 is a high‐affinity transporter whereas SMCT2 is a low‐affinity transporter. The physiological substrates for SMCT1 and SMCT2 are lactate (L‐lactic acid and D‐lactic acid), pyruvic acid, propanoic acid, and nicotinic acid in non‐colonic tissues such as the kidney. SMCT1 is also likely to be the principal transporter for the absorption of nicotinic acid (vitamin B_3_) in the intestine and kidney [197]. In the small intestine and colon, the physiological substrates for these transporters are nicotinic acid and the short‐chain fatty acids acetic acid, propanoic acid, and butyric acid that are produced by bacterial fermentation of dietary fiber [350]. In the kidney, SMCT2 is responsible for the bulk absorption of lactate because of its low‐affinity/high‐capacity nature. Absence of both transporters in the kidney leads to massive excretion of lactate in urine and consequently drastic decrease in the circulating levels of lactate in blood [470]. SMCT1 also functions as a tumour suppressor in the colon as well as in various other non‐colonic tissues [180]. The tumour‐suppressive function of SMCT1 is based on its ability to transport pyruvic acid, an inhibitor of histone deacetylases, into cells in non‐colonic tissues [471]; in the colon, the ability of SMCT1 to transport butyric acid and propanoic acid, also inhibitors of histone deacetylases, underlies the tumour‐suppressive function of this transporter [179, 180, 213]. The ability of SMCT1 to promote histone acetylase inhibition through accumulation of butyric acid and propanoic acid in immune cells is also responsible for suppression of dendritic cell development in the colon [451].

**Table:** 

Nomenclature	NIS	SMVT	SMCT1	SMCT2
Systematic nomenclature	SLC5A5	SLC5A6	SLC5A8	SLC5A12
HGNC, UniProt	SLC5A5, Q92911	SLC5A6, Q9Y289	SLC5A8, Q8N695	SLC5A12, Q1EHB4
Substrates	ClO_4_^‐^, SCN^‐^, I^‐^, NO_3_^‐^, pertechnetate	lipoic acid [556], pantothenic acid [556], I^‐^ [556], biotin [556]	propanoic acid, 3‐bromopyruvate, pyroglutamic acid, nicotinic acid, D‐lactic acid, *β*‐D‐hydroxybutyric acid, L‐lactic acid, salicylic acid, dichloroacetate, butyric acid, *α*‐ketoisocaproate, pyruvic acid, acetoacetic acid, benzoate, *γ*‐hydroxybutyric acid, 2‐oxothiazolidine‐4‐carboxylate, acetic acid, *β*‐L‐hydroxybutyric acid, 5‐aminosalicylate	pyruvic acid, L‐lactic acid, nicotinic acid
Stoichiometry	2Na^+^ : 1 I^‐^ [149]; 1Na^+^ : 1 ClO_4_^‐^ [123]	2Na^+^ : 1 biotin (or pantothenic acid) [390]	2Na^+^ : 1 monocarboxylate [94]	–
Inhibitors	–	–	fenoprofen (pIC_50_ 4.6) [248], ibuprofen (pIC_50_ 4.2) [248], ketoprofen (pIC_50_ 3.9) [248]	–

### Comments

I^‐^, ClO_4_^‐^, thiocyanate and NO_3_^‐^ are competitive substrate inhibitors of NIS [123]. Lipoic acid appears to act as a competitive substrate inhibitor of SMVT [505] and the anticonvulsant drugs primidone and carbamazepine competitively block the transport of biotin by brush border vesicles prepared from human intestine [423].

## Sodium myo‐inositol cotransporter transporters

### Overview

Three different mammalian myo‐inositol cotransporters are currently known; two are the Na^+^‐coupled SMIT1 and SMIT2 tabulated below and the third is proton‐coupled HMIT (SLC2A13). SMIT1 and SMIT2 have a widespread and overlapping tissue location but in polarized cells, such as the Madin‐Darby canine kidney cell line, they segregate to the basolateral and apical membranes, respectively [41]. In the nephron, SMIT1 mediates myo‐inositol uptake as a ‘compatible osmolyte’ when inner medullary tubules are exposed to increases in extracellular osmolality, whilst SMIT2 mediates the reabsorption of myo‐inositol from the filtrate. In some species (e.g. rat, but not rabbit) apically located SMIT2 is responsible for the uptake of myo‐inositol from the intestinal lumen [11].

**Table:** 

Nomenclature	SMIT	SGLT6
Systematic nomenclature	SLC5A3	SLC5A11
Common abreviation	SMIT1	SMIT2
HGNC, UniProt	SLC5A3, P53794	SLC5A11, Q8WWX8
Substrates	myo‐inositol, scyllo‐inositol>L‐fucose>L‐xylose>L‐glucose, D‐glucose, *α*‐MDG>D‐galactose, D‐fucose>D‐xylose [214]	myo‐inositol = D‐chiro‐inositol>D‐glucose>D‐xylose>L‐xylose [95]
Stoichiometry	2 Na^+^ :1 myo‐inositol [214]	2 Na^+^ :1 myo‐inositol [55]
Inhibitors	phlorizin [95]	phlorizin (p*K*_i_ 4.1) [95]

### Comments

The data tabulated are those for dog SMIT1 and rabbit SMIT2. SMIT2 transports D‐chiro‐inositol, but SMIT1 does not. In addition, whereas SMIT1 transports both D‐xylose and L‐xylose and D‐fucose and L‐fucose, SMIT2 transports only the D‐isomers of these sugars [95, 214]. Thus the substrate specificities of SMIT1 (for L‐fucose) and SMIT2 (for D‐chiro‐inositol) allow discrimination between the two SMITs. Human SMIT2 appears not to transport glucose [318].

### Further Reading

Bailey CJ. (2011) Renal glucose reabsorption inhibitors to treat diabetes. Trends Pharmacol. Sci. 32: 63‐71 [PMID:21211857]

Chao EC et al. (2010) SGLT2 inhibition–a novel strategy for diabetes treatment. Nat Rev Drug Discov 9: 551‐9 [PMID:20508640]

Darrouzet E et al. (2014) The sodium/iodide symporter: state of the art of its molecular characterization. Biochim. Biophys. Acta 1838: 244‐53 [PMID:23988430]

Haga T. (2014) Molecular properties of the high‐affinity choline transporter CHT1. J. Biochem. 156: 181‐94 [PMID:25073461]

Kinne RK et al. (2011) SGLT inhibitors as new therapeutic tools in the treatment of diabetes. Handb Exp Pharmacol 105‐26 [PMID:21484569]

Whalen K et al. (2015) The Role of Sodium‐Glucose Co‐Transporter 2 Inhibitors in the Treatment of Type 2 Diabetes. Clin Ther 37: 1150‐66 [PMID:25891804]

Wilding JP. (2014) The role of the kidneys in glucose homeostasis in type 2 diabetes: clinical implications and therapeutic significance through sodium glucose co‐transporter 2 inhibitors. Metab. Clin. Exp. 63: 1228‐37 [PMID:25104103]

Wright EM. (2013) Glucose transport families SLC5 and SLC50. Mol. Aspects Med. 34: 183‐96 [PMID:23506865]

Wright EM et al. (2011) Biology of human sodium glucose transporters. Physiol. Rev. 91: 733‐94 [PMID:21527736]

## SLC6 neurotransmitter transporter family

### Overview

Members of the solute carrier family 6 (SLC6) of sodium‐ and (sometimes chloride‐) dependent neurotransmitter transporters [64, 83, 292] are primarily plasma membrane located and may be divided into four subfamilies that transport monoamines, GABA, glycine and neutral amino acids, plus the related bacterial NSS transporters [424]. The members of this superfamily share a structural motif of 10 TM segments that has been observed in crystal structures of the NSS bacterial homolog LeuT_Aa_, a Na^+^‐dependent amino acid transporter from Aquiflex aeolicus [527] and in several other transporter families structurally related to LeuT [167].

## Monoamine transporter subfamily

### Overview

Monoamine neurotransmission is limited by perisynaptic transporters. Presynaptic monoamine transporters allow recycling of synaptically released noradrenaline, dopamine and 5‐hydroxytryptamine.

**Table:** 

Nomenclature	NET	DAT	SERT
Systematic nomenclature	SLC6A2	SLC6A3	SLC6A4
HGNC, UniProt	SLC6A2, P23975	SLC6A3, Q01959	SLC6A4, P31645
Substrates	MPP^+^, methamphetamine, amphetamine	MPP^+^, methamphetamine, amphetamine	MDMA, p‐chloroamphetamine
Endogenous substrates	dopamine, (‐)‐adrenaline, (‐)‐noradrenaline	dopamine, (‐)‐adrenaline, (‐)‐noradrenaline	5‐hydroxytryptamine
Stoichiometry	1 noradrenaline: 1 Na^+^:1 Cl^‐^ [211]	1 dopamine: 1‐2 Na^+^: 1 Cl^‐^ [210]	1 5‐HT:1 Na^+^:1 Cl^‐^ (in), + 1 K^+^ (out) [465]
Inhibitors	milnacipran (pIC_50_ 9.1) [490], atomoxetine (p*K*_d_ 8.7) [72], desipramine (p*K*_i_ 8.7) [368], lofepramine (p*K*_i_ 8.3) [468], duloxetine (p*K*_i_ 8.2) [382], nortriptyline (p*K*_i_ 8.2) [192], amoxapine (p*K*_i_ 7.9) [21], imipramine (p*K*_i_ 7.8), doxepin (p*K*_i_ 7.5) [21], clomipramine (p*K*_d_ 7.4) [468], levomilnacipran (pIC_50_ 7.4) [499], dosulepin (p*K*_i_ 7.3) [468], dexamfetamine (p*K*_i_ 7) [17], amitriptyline (p*K*_i_ 6.5) [17], nefazodone (p*K*_d_ 6.4) [72], bupropion (p*K*_i_ 6.4) [296], trimipramine (p*K*_i_ 5.6) [468], tapentadol (p*K*_i_ 5.1) [484]	cocaine (pIC_50_ 7.1) [74] – Rat, dexamfetamine (p*K*_i_ 7) [17], bupropion (pIC_50_ 6.3) [73], atomoxetine (p*K*_d_ 6) [72], trimipramine (p*K*_i_ 5.4) [468], nomifensine	vilazodone (pIC_50_ 8.8–9.3) [108, 226], vortioxetine (p*K*_i_ 8.8) [30], duloxetine (p*K*_i_ 8.3) [163], nortriptyline (p*K*_i_ 8.2) [21], dosulepin (p*K*_i_ 8.1) [468], atomoxetine (p*K*_d_ 8.1) [72], desvenlafaxine (p*K*_i_ 7.8) [128], amoxapine (p*K*_i_ 7.7) [21], imipramine (p*K*_i_ 7.7) [462], milnacipran (pIC_50_ 7.3) [490], lofepramine (p*K*_i_ 7.2) [468], trimipramine (p*K*_i_ 6.8) [468], desipramine (p*K*_i_ 6.8) [377], nefazodone (p*K*_d_ 6.7) [72], levomilnacipran (pIC_50_ 6.5) [499]
(Sub)family‐selective inhibitors	desvenlafaxine (p*K*_i_<6.2) [128], sibutramine (p*K*_i_ 5.2) [21]	sibutramine (p*K*_i_ 6.3) [21]	sibutramine (p*K*_i_ 6) [21]
Selective inhibitors	mazindol (p*K*_i_ 8.9), protriptyline (pIC_50_ 8.8) [352], nisoxetine (p*K*_i_ 8.4), protriptyline (p*K*_i_ 8.2) [320], nomifensine (p*K*_i_ 8.1), reboxetine (p*K*_i_ 8) [517], maprotiline (p*K*_i_ 7.9) [225], methylphenidate (pIC_50_ 7.2)	mazindol (p*K*_i_ 8), WIN35428 (p*K*_i_ 7.9) [404], GBR12935 (p*K*_i_ 7.6), dexmethylphenidate (p*K*_i_ 7.6) [297], methylphenidate (pIC_50_ 7.1) [170]	clomipramine (p*K*_i_ 9.7) [468], paroxetine (p*K*_i_ 9.6) [468], clomipramine (p*K*_d_ 9.6) [468], sertraline (p*K*_i_ 9.1), escitalopram (pIC_50_ 9) [413], dapoxetine (pIC_50_ 8.9) [186], fluvoxamine (p*K*_d_ 8.7) [468], fluoxetine (p*K*_i_ 8.5) [468], citalopram (p*K*_i_ 8.4) [34], protriptyline (p*K*_d_ 7.7) [468], venlafaxine (pIC_50_ 7.6) [419], amitriptyline (p*K*_i_ 6.8) [17]
Labelled ligands	[^3^H]mazindol (Inhibitor) (p*K*_d_ 9.3) [396] – Rat, [^3^H]nisoxetine (Inhibitor) (p*K*_d_ 8.4)	[^3^H]GBR12935 (Inhibitor) (p*K*_d_ 8.5) [391], [^3^H]WIN35428 (Inhibitor) (p*K*_d_ 8) [391]	[^3^H]paroxetine (Inhibitor) (p*K*_d_ 9.7), [^3^H]citalopram (Inhibitor) (p*K*_d_ 8.3)

### Comments

[^125^I]RTI55 labels all three monoamine transporters (NET, DAT and SERT) with affinities between 0.5 and 5 nM. Cocaine is an inhibitor of all three transporters with pK_i_ values between 6.5 and 7.2. Potential alternative splicing sites in non‐coding regions of SERT and NET have been identified. A bacterial homologue of SERT shows allosteric modulation by selected anti‐depressants [452].

## GABA transporter subfamily

### Overview

The activity of GABA‐transporters located predominantly upon neurones (GAT‐1), glia (GAT‐3) or both (GAT‐2, BGT‐1) serves to terminate phasic GABA‐ergic transmission, maintain low ambient extracellular concentrations of GABA, and recycle GABA for reuse by neurones. Nonetheless, ambient concentrations of GABA are sufficient to sustain tonic inhibition mediated by high affinity GABA_A_ receptors in certain neuronal populations [442]. GAT1 is the predominant GABA transporter in the brain and occurs primarily upon the terminals of presynaptic neurones and to a much lesser extent upon distal astocytic processes that are in proximity to axons terminals. GAT3 resides predominantly on distal astrocytic terminals that are close to the GABAergic synapse. By contrast, BGT1 occupies an extrasynaptic location possibly along with GAT2 which has limited expression in the brain [329]. TauT is a high affinity taurine transporter involved in osmotic balance that occurs in the brain and non‐neuronal tissues, such as the kidney, brush border membrane of the intestine and blood brain barrier [83, 219]. CT1, which transports creatine, has a ubiquitous expression pattern, often co‐localizing with creatine kinase [83].

**Table:** 

Nomenclature	GAT1	GAT2	GAT3
Systematic nomenclature	SLC6A1	SLC6A13	SLC6A11
HGNC, UniProt	SLC6A1, P30531	SLC6A13, Q9NSD5	SLC6A11, P48066
Substrates	nipecotic acid, guvacine	nipecotic acid, guvacine	guvacine, nipecotic acid
Endogenous substrates	GABA	*β*‐alanine, GABA	*β*‐alanine, GABA
Stoichiometry	2Na^+^: 1Cl^‐^: 1GABA	2Na^+^: 1Cl^‐^:1GABA	≥ 2Na^+^: 2 Cl^‐^: 1GABA
Selective inhibitors	NNC‐711 (pIC_50_ 7.4) [50], tiagabine (pIC_50_ 7.2) [50], SKF89976A (pIC_50_ 6.9) [117], CI‐966 (pIC_50_ 6.6) [50], (R/S) EF‐1500 (pIC_50_ 4.9–5.7), (R)‐EF‐1520 (pIC_50_ 5.1–5.4), LU32‐176B (pIC_50_ 5.4) [512] – Mouse, (S)‐EF‐1520 (pIC_50_ 3.6–3.9)	SNAP‐5114 (pIC_50_ 4.7) [49] – Rat	–
Labelled ligands	[^3^H]tiagabine (Inhibitor)	–	–

**Table:** 

Nomenclature	BGT1	TauT	CT1
Systematic nomenclature	SLC6A12	SLC6A6	SLC6A8
HGNC, UniProt	SLC6A12, P48065	SLC6A6, P31641	SLC6A8, P48029
Endogenous substrates	GABA, betaine	*β*‐alanine, taurine, GABA [9]	creatine
Stoichiometry	3Na^+^: 1 (or 2) Cl^‐^: 1GABA	2Na^+^: 1Cl^‐^: 1 taurine	Probably 2Na^+^: 1Cl^‐^: 1 creatine
Selective inhibitors	NNC052090 (p*K*_i_ 5.9) [473] – Mouse, (R/S) EF‐1500 (pIC_50_ 4.9), (R)‐EF‐1520 (pIC_50_ 3.7–4.7), (S)‐EF‐1520 (pIC_50_ 3.6–4.5), LU32‐176B (pIC_50_ 4) [512] – Mouse	–	–

### Comments

The IC_50_ values for GAT1‐4 reported in the table reflect the range reported in the literature from studies of both human and mouse transporters. There is a tendency towards lower IC_50_ values for the human orthologue [295]. SNAP‐5114 is only weakly selective for GAT 2 and GAT3, with IC_50_ values in the range 22 to >30 *μ*M at GAT1 and BGT1, whereas NNC052090 has at least an order of magnitude selectivity for BGT1 [see [93, 438] for reviews]. Compound (R)‐4d [PMID: 16766089] is a recently described compound that displays 20‐fold selectivity for GAT3 over GAT1 [174]. In addition to the inhibitors listed, deramciclane is a moderately potent, though non‐selective, inhibitor of all cloned GABA transporters (IC_50_ = 26‐46 *μ*M; [116]). Diaryloxime and diarylvinyl ether derivatives of nipecotic acid and guvacine that potently inhibit the uptake of [^3^H]GABA into rat synaptosomes have been described [282]. Several derivatives of exo‐THPO(e.g. N‐methyl‐exo‐THPO and N‐acetyloxyethyl‐exo‐THPO) demonstrate selectivity as blockers of astroglial, versus neuronal, uptake of GABA[see [93, 437] for reviews]. GAT3 is inhibited by physiologically relevant concentrations of Zn^2+^[96]. Taut transports GABA, but with low affinity, but CT1 does not, although it can be engineered to do so by mutagenesis guided by LeuT as a structural template [121]. Although inhibitors of creatine transport by CT1 (e.g. *β*‐guanidinopropionic acid, cyclocreatine, guanidinoethane sulfonic acid) are known (e.g. [103]) they insufficiently characterized to be included in the table.

## Glycine transporter subfamily

### Overview

Two gene products, GlyT1 and GlyT2, are known that give rise to transporters that are predominantly located on glia and neurones, respectively. Five variants of GlyT1 (a,b,c,d & e) differing in their N‐ and C‐termini are generated by alternative promoter usage and splicing, and three splice variants of GlyT2 (a,b & c) have also been identified (see [36, 152, 194, 459] for reviews). GlyT1 transporter isoforms expressed in glia surrounding glutamatergic synapses regulate synaptic glycine concentrations influencing NMDA receptor‐mediated neurotransmission [35, 175], but also are important, in early neonatal life, for regulating glycine concentrations at inhibitory glycinergic synapses [195]. Homozygous mice engineered to totally lack GlyT1 exhibit severe respiratory and motor deficiencies due to hyperactive glycinergic signalling and die within the first postnatal day [195, 479]. Disruption of GlyT1 restricted to forebrain neurones is associated with enhancement of EPSCs mediated by NMDA receptors and behaviours that are suggestive of a promnesic action [532]. GlyT2 transporters localised on the axons and boutons of glycinergic neurones appear crucial for efficient transmitter loading of synaptic vesicles but may not be essential for the termination of inhibitory neurotransmission [196, 415]. Mice in which GlyT2 has been deleted develop a fatal hyperekplexia phenotype during the second postnatal week [196] and mutations in the human gene encoding GlyT2 (SLC6A5) have been identified in patients with hyperekplexia (reviewed by [221]). ATB^0+^(SLCA14) is a transporter for numerous dipolar and cationic amino acids and thus has a much broader substrate specificity than the glycine transporters alongside which it is grouped on the basis of structural similarity [83]. ATB^0+^ is expressed in various peripheral tissues [83]. By contrast PROT (SLC6A7), which is expressed only in brain in association with a subset of excitatory nerve terminals, shows specificity for the transport of L‐proline.

**Table:** 

Nomenclature	GlyT1	GlyT2	ATB^0,+^	PROT
Systematic nomenclature	SLC6A9	SLC6A5	SLC6A14	SLC6A7
HGNC, UniProt	SLC6A9, P48067	SLC6A5, Q9Y345	SLC6A14, Q9UN76	SLC6A7, Q99884
Endogenous substrates	–	–	L‐isoleucine>L‐leucine, L‐methionine>L‐phenylalanine>L‐tryptophan>L‐valine>L‐serine [453]	–
Substrates	–	–	BCH, zwitterionic or cationic NOS inhibitors [224], 1‐methyltryptophan [270], valganciclovir [488]	–
Endogenous substrates	sarcosine, glycine	glycine	*β*‐alanine [8, 9]	L‐proline
Stoichiometry	2 Na^+^: 1 Cl^‐^: 1 glycine	3 Na^+^: 1 Cl^‐^: 1 glycine	2‐3 Na^+^: 1 Cl^‐^: 1 amino acid [453]	Probably 2 Na^+^: 1 Cl^‐^: 1 L‐proline
Inhibitors	–	bitopertin (pEC_50_<4.5) [385]	–	–
Selective inhibitors	(R)‐NFPS (pIC_50_ 8.5–9.1), SSR‐103800 (pIC_50_ 8.7) [54], N‐methyl‐SSR504734 (pIC_50_ 8.6), LY2365109 (pIC_50_ 7.8), GSK931145 (pIC_50_ 7.6), bitopertin (pEC_50_ 7.5) [385]	Org 25543 (pIC_50_ 7.8) [76], ALX 1393, ALX 1405	*α*‐methyl‐D,L‐tryptophan (pIC_50_ 3.6) [270]	compound 58 [PMID: 25037917] (pIC_50_ 7.7) [553], LP‐403812 (pIC_50_ 7) [535]
Labelled ligands	[^3^H](R)‐NPTS (Binding) (p*K*_d_ 9) [323], [^3^H]GSK931145 (Binding) (p*K*_d_ 8.8) [229], [^35^S]ACPPB (Binding) (p*K*_d_ 8.7) [540], [^3^H]SB‐733993 (Binding) (p*K*_d_ 8.7) [229], [^3^H]N‐methyl‐SSR504734 (p*K*_d_ 8.1–8.5), [^3^H]NFPS (p*K*_d_ 7.7–8.2)	–	–	–
Comments	–	N‐Oleoyl‐L‐carnitine (0.3*μ*M, [71]) and and N‐arachidonoylglycine (IC_50_ 5‐8 *μ*M, [513]) have been described as potential endogenous selective GlyT2 inhibitors	–	–

### Comments

sarcosine is a selective transportable inhibitor of GlyT1 and also a weak agonist at the glycine binding site of the NMDA receptor [544], but has no effect on GlyT2. This difference has been attributed to a single glycine residue in TM6 (serine residue in GlyT2) [493]. Inhibition of GLYT1 by the sarcosine derivatives NFPS, NPTS and Org 24598 is non‐competitive [331, 341]. IC_50_ values for Org 24598 reported in the literature vary, most likely due to differences in assay conditions [58, 331]. The tricyclic antidepressant amoxapine weakly inhibits GlyT2 (IC_50_ 92 *μ*M) with approximately 10‐fold selectivity over GlyT1 [366]. The endogenous lipids arachidonic acid and anandamide exert opposing effects upon GlyT1a, inhibiting (IC_50_  2 *μ*M) and potentiating (EC_50_  13 *μ*M) transport currents, respectively [381]. N‐arachidonyl‐glycine, N‐arachidonyl‐*γ*‐aminobutyric acid and N‐arachidonyl‐D‐alanine have been described as endogenous non‐competitive inhibitors of GlyT2a, but not GlyT1b [136, 253, 513]. Protons [20] and Zn^2+^[257] act as non‐competitive inhibitors of GlyT1b, with IC_50_ values of  100 nM and  10 *μ*M respectively, but neither ion affects GlyT2 (reviewed by [491]). Glycine transport by GLYT1 is inhibited by Li^+^, whereas GLYT2 transport is stimulated (both in the presence of Na^+^) [392].

## Neutral amino acid transporter subfamily

### Overview

Certain members of neutral amino acid transport family are expressed upon the apical surface of epithelial cells and are important for the absorption of amino acids from the duodenum, jejunum and ileum and their reabsorption within the proximal tubule of the nephron (i.e. B^0^AT1 (SLC6A19), SLC6A17, SLC6A18, SLC6A20). Others may function as transporters for neurotransmitters or their precursors (i.e. B^0^AT2, SLC6A17) [65].

**Table:** 

Nomenclature	B^0^AT1	B^0^AT2	B^0^AT3
Systematic nomenclature	SLC6A19	SLC6A15	SLC6A18
HGNC, UniProt	SLC6A19, Q695T7	SLC6A15, Q9H2J7	SLC6A18, Q96N87
Endogenous substrates	L‐leucine, L‐methionine, L‐isoleucine, L‐valine>L‐asparagine, L‐phenylalanine, L‐alanine, L‐serine>L‐threonine, glycine, L‐proline [64]	L‐proline>L‐alanine, L‐valine, L‐methionine, L‐leucine>L‐isoleucine, L‐threonine, L‐asparagine, L‐serine, L‐phenylalanine>glycine [64]	L‐alanine, glycine>L‐methionine, L‐phenylalanine, L‐leucine, L‐histidine, L‐glutamine [494]
Stoichiometry	1 Na^+^: 1 amino acid [70]	1 Na^+^: 1 amino acid [62]	Na^+^‐ and Cl^‐^ ‐dependent transport [450]
Inhibitors	nimesulide (pIC_50_ 4.6) [387] – Rat	–	–
Selective inhibitors	–	loratadine (pIC_50_ 5.4) [102]	–
Comments	Mutations in B^0^AT1 are associated with Hartnup disorder	–	–

**Table:** 

Nomenclature	NTT5	NTT4	SIT1
Systematic nomenclature	SLC6A16	SLC6A17	SLC6A20
HGNC, UniProt	SLC6A16, Q9GZN6	SLC6A17, Q9H1V8	SLC6A20, Q9NP91
Endogenous substrates	–	L‐leucine, L‐methionine, L‐proline>L‐cysteine, L‐alanine, L‐glutamine, L‐serine>L‐histidine, glycine [537]	–
Endogenous substrates	–	–	L‐proline
Stoichiometry	–	Na^+^‐dependent, Cl^‐^‐independent transport [537]	2 Na^+^: 1 Cl^‐^: 1 imino acid [60]

### Further Reading

Bröer S et al. (2012) The solute carrier 6 family of transporters. Br. J. Pharmacol. 167: 256‐78 [PMID:22519513]

Chiba P et al. (2014) Defining the blanks–pharmacochaperoning of SLC6 transporters and ABC transporters. Pharmacol. Res. 83: 63‐73 [PMID:24316454]

Harvey RJ et al. (2013) Glycine transporters as novel therapeutic targets in schizophrenia, alcohol dependence and pain. Nat Rev Drug Discov 12: 866‐85 [PMID:24172334]

Kempson SA et al. (2014) The betaine/GABA transporter and betaine: roles in brain, kidney, and liver. Front Physiol 5: 159 [PMID:24795654]

Penmatsa A et al. (2013) X‐ray structure of dopamine transporter elucidates antidepressant mechanism. Nature 503: 85‐90 [PMID:24037379]

Pramod AB et al. (2013) SLC6 transporters: structure, function, regulation, disease association and therapeutics. Mol. Aspects Med. 34: 197‐219 [PMID:23506866]

Ramamoorthy S et al. (2011) Regulation of monoamine transporters: Role of transporter phosphorylation. Pharmacol. Ther. 129: 220‐38 [PMID:20951731]

Reynolds GP et al. (2014) Pharmacogenomics in psychiatry: the relevance of receptor and transporter polymorphisms. Br J Clin Pharmacol 77: 654‐72 [PMID:24354796]

Rudnick G. (2011) Cytoplasmic permeation pathway of neurotransmitter transporters. Biochemistry 50: 7462‐75 [PMID:21774491]

Rudnick G et al. (2014) The SLC6 transporters: perspectives on structure, functions, regulation, and models for transporter dysfunction. Pflugers Arch. 466: 25‐42 [PMID:24337881]

Vandenberg RJ et al. (2014) Glycine transport inhibitors for the treatment of pain. Trends Pharmacol. Sci. 35: 423‐30 [PMID:24962068]

Zhong H et al. (2012) Consideration of allosterism and interacting proteins in the physiological functions of the serotonin transporter. Biochem. Pharmacol. 83: 435‐42 [PMID:21983034]

## SLC8 family of sodium/calcium exchangers

### Overview

The sodium/calcium exchangers (NCX) use the extracellular sodium concentration to facilitate the extrusion of calcium out of the cell. Alongside the plasma membrane Ca^2+^‐ATPase (PMCA) and sarcoplasmic/endoplasmic reticulum Ca^2+^‐ATPase (SERCA), as well as the sodium/potassium/calcium exchangers (NKCX, SLC24 family), NCX allow recovery of intracellular calcium back to basal levels after cellular stimulation. When intracellular sodium ion levels rise, for example, following depolarisation, these transporters can operate in the reverse direction to allow calcium influx and sodium efflux, as an electrogenic mechanism. Structural modelling suggests the presence of 9 TM segments, with a large intracellular loop between the fifth and sixth TM segments.

**Table:** 

Nomenclature	Sodium/calcium exchanger 1	Sodium/calcium exchanger 2	Sodium/calcium exchanger 3
Systematic nomenclature	SLC8A1	SLC8A2	SLC8A3
Common abreviation	NCX1	NCX2	NCX3
HGNC, UniProt	SLC8A1, P32418	SLC8A2, Q9UPR5	SLC8A3, P57103
Stoichiometry	3 Na^+^ (in) : 1 Ca^2+^ (out) or 4 Na^+^ (in) : 1 Ca^2+^ (out) [124]; Reverse mode 1 Ca^2+^ (in): 1 Na^+^ (out)	–	–

### Comments

Although subtype‐selective inhibitors of NCX function are not widely available, 3,4‐dichlorobenzamil and CBDMB act as non‐selective NCX inhibitors, while SEA0400, KB‐R7943, SN6, and ORM‐10103[256] act to inhibit NCX function with varying degrees of selectivity. BED is a selective NCX3 inhibitor [439].

### Further Reading

Annunziato L et al. (2004) Pharmacology of brain Na+/Ca2+ exchanger: from molecular biology to therapeutic perspectives. Pharmacol. Rev. 56: 633‐54 [PMID:15602012]

Khananshvili D. (2014) Sodium‐calcium exchangers (NCX): molecular hallmarks underlying the tissue‐specific and systemic functions. Pflugers Arch. 466: 43‐60 [PMID:24281864]

Lytton J. (2007) Na+/Ca2+ exchangers: three mammalian gene families control Ca2+ transport. Biochem. J. 406: 365‐82 [PMID:17716241]

Quednau BD et al. (2004) The sodium/calcium exchanger family‐SLC8. Pflugers Arch. 447: 543‐8 [PMID:12734757]

Watanabe Y et al. (2006) Topics on the Na+/Ca2+ exchanger: pharmacological characterization of Na+/Ca2+ exchanger inhibitors. J. Pharmacol. Sci. 102: 7‐16 [PMID:16990699]

## SLC9 family of sodium/hydrogen exchangers

### Overview

Sodium/hydrogen exchangers or sodium/proton antiports are a family of transporters that maintain cellular pH by utilising the sodium gradient across the plasma membrane to extrude protons produced by metabolism, in a stoichiometry of 1 Na^+^ (in) : 1 H^+^(out). Several isoforms, NHE6, NHE7, NHE8 and NHE9 appear to locate on intracellular membranes [351, 357, 365]. Li^+^ and NH_4_^+^, but not K^+^, ions may also be transported by some isoforms. Modelling of the topology of these transporters indicates 12 TM regions with an extended intracellular C‐terminus containing multiple regulatory sites.

NHE1 is considered to be a ubiquitously‐expressed ‘housekeeping’ transporter. NHE3 is highly expressed in the intestine and kidneys and regulate sodium movements in those tissues. NHE10 is present in sperm [504] and osteoclasts [307]; gene disruption results in infertile male mice [504].

### Comments

Analogues of the non‐selective cation transport inhibitor amiloride appear to inhibit NHE function through competitive inhibition of the extracellular Na^+^ binding site. The more selective amiloride analogues MPA and ethylisopropylamiloride exhibit a rank order of affinity of inhibition of NHE1 > NHE2 > NHE3 [100, 480, 481].

### Further Reading

Bobulescu IA et al. (2009) Luminal Na(+)/H (+) exchange in the proximal tubule. Pflugers Arch. 458: 5‐21 [PMID:18853182]

Casey JR et al. (2010) Sensors and regulators of intracellular pH. Nat. Rev. Mol. Cell Biol. 11: 50‐61 [PMID:19997129]

Christensen HL et al. (2013) Na(+) dependent acid‐base transporters in the choroid plexus; insights from slc4 and slc9 gene deletion studies. Front Physiol 4: 304 [PMID:24155723]

Donowitz M et al. (2013) SLC9/NHE gene family, a plasma membrane and organellar family of Na^+^/H^+^ exchangers. Mol. Aspects Med. 34: 236‐51 [PMID:23506868]

Kato A et al. (2011) Regulation of electroneutral NaCl absorption by the small intestine. Annu. Rev. Physiol. 73: 261‐81 [PMID:21054167]

Kemp G et al. (2008) Structure and function of the human Na+/H+ exchanger isoform 1. Channels (Austin) 2: 329‐36 [PMID:19001864]

Ohgaki R et al. (2011) Organellar Na+/H+ exchangers: novel players in organelle pH regulation and their emerging functions. Biochemistry 50: 443‐50 [PMID:21171650]

Parker MD et al. (2015) Na+‐H+ exchanger‐1 (NHE1) regulation in kidney proximal tubule. Cell. Mol. Life Sci. 72: 2061‐74 [PMID:25680790]

Ruffin VA et al. (2014) Intracellular pH regulation by acid‐base transporters in mammalian neurons. Front Physiol 5: 43 [PMID:24592239]

## SLC10 family of sodium‐bile acid co‐transporters

### Overview

The SLC10 family transport bile acids, sulphated solutes, and other xenobiotics in a sodium‐dependent manner. The founding members, SLC10A1 (NTCP) and SLC10A2 (ASBT) function, along with members of the ABC transporter family (MDR1/ABCB1, BSEP/ABCB11 and MRP2/ABCC2) and the organic solute transporter obligate heterodimer OST*α*:OST*β*(SLC51), to maintain the enterohepatic circulation of bile acids [110, 281]. SLC10A6 (SOAT) functions as a sodium‐dependent transporter of sulphated solutes included sulfphated steroids and bile acids [187, 189]. Transport function has not yet been demonstrated for the 4 remaining members of the SLC10 family, SLC10A3 (P3), SLC10A4 (P4), SLC10A5 (P5), and SLC10A7 (P7), and the identity of their endogenous substrates remain unknown [160, 189, 193, 500]. Members of the SLC10 family are predicted to have seven transmembrane domains with an extracellular N‐terminus and cytoplasmic C‐terminus [29, 215].

**Table:** 

Nomenclature	Sodium/bile acid and sulphated solute	Sodium/bile acid and sulphated solute	Sodium/bile acid and sulphated solute
	cotransporter 1	cotransporter 2	cotransporter 6
Systematic nomenclature	SLC10A1	SLC10A2	SLC10A6
Common abreviation	NTCP	ASBT	SOAT
HGNC, UniProt	SLC10A1, Q14973	SLC10A2, Q12908	SLC10A6, Q3KNW5
Substrates	tauroursodeoxycholic acid, taurocholic acid, taurochenodeoxycholic acid>glycocholic acid>cholic acid [336]	glycodeoxycholic acid>glycoursodeoxycholic acid, glycochenodeoxycholic acid>taurocholic acid>cholic acid [101]	–
Substrates	–	–	pregnenolone sulphate [187], estrone‐3‐sulphate, dehydroepiandrosterone sulphate [189], taurolithocholic acid‐3‐sulphate
Endogenous substrates	triiodothyronine, dehydroepiandrosterone sulphate [101, 160, 336], estrone‐3‐sulphate, iodothyronine sulphates	–	–
Stoichiometry	2 Na^+^: 1 bile acid [29, 187]	>1 Na^+^: 1 bile acid [101, 509]	–
Inhibitors	(‐)‐propranolol (pIC_50_ 8.2) [279], cyclosporin A (pIC_50_ 6) [279], (+)‐propranolol (pIC_50_ 5.3) [279], cyclosporin A (p*K*_i_ 5.1) [125], irbesartan (p*K*_i_ 4.9) [125]	SC‐435 (pIC_50_ 8.8) [38], 264W94 (pIC_50_ 7.3) [475, 522]	–
Labelled ligands	–	[^3^H]taurocholic acid [101]	–
Comments	chenodeoxycholyl‐N^*ε*^‐nitrobenzoxadiazol‐lysine is a fluorescent bile acid analogue used as a probe [188, 509].	–	–

### Comments

Heterologously expressed SLC10A4 [188] or SLC10A7 [193] failed to exhibit significant transport of taurocholic acid, pregnenolone sulphate, dehydroepiandrosterone sulphate or choline. SLC10A4 has recently been suggested to associate with neuronal vesicles [68].

### Further Reading

Anwer MS et al. (2014) Sodium‐dependent bile salt transporters of the SLC10A transporter family: more than solute transporters. Pflugers Arch. 466: 77‐89 [PMID:24196564]

Borges K. (2013) Slc10A4 ‐ what do we know about the function of this ~secret ligand carrier~ protein? Exp. Neurol. 248C: 258‐261 [PMID:23810836]

Claro da Silva T et al. (2013) The solute carrier family 10 (SLC10): beyond bile acid transport. Mol. Aspects Med. 34: 252‐69 [PMID:23506869]

Dawson PA et al. (2009) Bile acid transporters. J. Lipid Res. 50: 2340‐57 [PMID:19498215]

Döring B et al. (2012) The SLC10 carrier family: transport functions and molecular structure. Curr Top Membr 70: 105‐68 [PMID:23177985]

Zwicker BL et al. (2013) Transport and biological activities of bile acids. Int. J. Biochem. Cell Biol. 45: 1389‐98 [PMID:23603607]

## SLC11 family of proton‐coupled metal ion transporters

### Overview

The family of proton‐coupled metal ion transporters are responsible for movements of divalent cations, particularly ferrous and manganese ions, across the cell membrane (SLC11A2/DMT1) and across endosomal (SLC11A2/DMT1) or lysosomal/phagosomal membranes (SLC11A1/NRAMP1), dependent on proton transport. Both proteins appear to have 12 TM regions and cytoplasmic N‐ and C‐ termini. NRAMP1 is involved in antimicrobial action in macrophages, although its precise mechanism is undefined. Facilitated diffusion of divalent cations into phagosomes may increase intravesicular free radicals to damage the pathogen. Alternatively, export of divalent cations from the phagosome may deprive the pathogen of essential enzyme cofactors. SLC11A1/DMT1 is more widely expressed and appears to assist in divalent cation assimilation from the diet, as well as in phagocytotic cells.

**Table:** 

Nomenclature	NRAMP1	DMT1
Systematic nomenclature	SLC11A1	SLC11A2
HGNC, UniProt	SLC11A1, P49279	SLC11A2, P49281
Endogenous substrates	Fe^2+^, Mn^2+^	Cu^2+^, Co^2+^, Cd^2+^, Fe^2+^, Mn^2+^
Stoichiometry	1 H^+^ : 1 Fe^2+^ (out) or 1 Fe^2+^ (in) : 1 H^+^ (out)	1 H^+^ : 1 Fe^2+^ (out) [212]

### Comments

Loss‐of‐function mutations in NRAMP1 are associated with increased susceptibility to microbial infection (OMIM: 607948). Loss‐of‐function mutations in DMT1 are associated with microcytic anemia (OMIM: 206100).

### Further Reading

Codazzi F et al. (2015) Iron entry in neurons and astrocytes: a link with synaptic activity. Front Mol Neurosci 8: 18 [PMID:26089776]

Li X et al. (2011) SLC11A1 (NRAMP1) polymorphisms and tuberculosis susceptibility: updated systematic review and meta‐analysis. PLoS ONE 6: e15831 [PMID:21283567]

Mackenzie B et al. (2004) SLC11 family of H+‐coupled metal‐ion transporters NRAMP1 and DMT1. Pflugers Arch. 447: 571‐9 [PMID:14530973]

Montalbetti N et al. (2013) Mammalian iron transporters: families SLC11 and SLC40. Mol. Aspects Med. 34: 270‐87 [PMID:23506870]

Nevo Y et al. (2006) The NRAMP family of metal‐ion transporters. Biochim. Biophys. Acta 1763: 609‐20 [PMID:16908340]

Skjørringe T et al. (2015) Divalent metal transporter 1 (DMT1) in the brain: implications for a role in iron transport at the blood‐brain barrier, and neuronal and glial pathology. Front Mol Neurosci 8: 19 [PMID:26106291]

Wessling‐Resnick M. (2015) Nramp1 and Other Transporters Involved in Metal Withholding during Infection. J. Biol. Chem. 290: 18984‐90 [PMID:26055722]

Zheng W et al. (2012) Regulation of brain iron and copper homeostasis by brain barrier systems: implication in neurodegenerative diseases. Pharmacol. Ther. 133: 177‐88 [PMID:22115751]

## SLC12 family of cation‐coupled chloride transporters

### Overview

The SLC12 family of chloride transporters contribute to ion fluxes across a variety of tissues, particularly in the kidney and choroid plexus of the brain. Within this family, further subfamilies are identifiable: NKCC1, NKCC2 and NCC constitute a group of therapeutically‐relevant transporters, targets for loop and thiazide diuretics. These 12 TM proteins exhibit cytoplasmic termini and an extended extracellular loop at TM7/8 and are kidney‐specific (NKCC2 and NCC) or show a more widespread distribution (NKCC1). A second family, the K‐Cl co‐transporters are also 12 TM domain proteins with cytoplasmic termini, but with an extended extracellular loop at TM 5/6. CCC6 exhibits structural similarities with the K‐Cl co‐transporters, while CCC9 is divergent, with 11 TM domains and a cytoplasmic N‐terminus and extracellular C‐terminus.

**Table:** 

Nomenclature	Kidney‐specific Na‐K‐Cl symporter	Basolateral Na‐K‐Cl symporter	Na‐Cl symporter	K‐Cl cotransporter 1
Systematic nomenclature	SLC12A1	SLC12A2	SLC12A3	SLC12A4
Common abreviation	NKCC2	NKCC1	NCC	KCC1
HGNC, UniProt	SLC12A1, Q13621	SLC12A2, P55011	SLC12A3, P55017	SLC12A4, Q9UP95
Stoichiometry	1 Na^+^ : 1 K^+^ : 2 Cl^‐^ (in)	1 Na^+^ : 1 K^+^ : 2 Cl^‐^ (in)	1 Na^+^ : 1 Cl^‐^ (in)	1 K^+^ : 1 Cl^‐^ (out)
Inhibitors	bumetanide (pIC_50_ 6.5) [220], piretanide (pIC_50_ 6) [220], furosemide (pIC_50_ 5.2) [220]	piretanide (pIC_50_ 5.6) [220], bumetanide (pIC_50_ 5.6) [220], furosemide (pIC_50_ 5.1) [220]	chlorothiazide, cyclothiazide, hydrochlorothiazide, metolazone	DIOA

**Table:** 

Nomenclature	K‐Cl cotransporter 2	K‐Cl cotransporter 3	K‐Cl cotransporter 4	Cation‐chloride cotransporter 9
Systematic nomenclature	SLC12A5	SLC12A6	SLC12A7	SLC12A8
Common abreviation	KCC2	KCC3	KCC4	CCC9
HGNC, UniProt	SLC12A5, Q9H2X9	SLC12A6, Q9UHW9	SLC12A7, Q9Y666	SLC12A8, A0AV02
Substrates	–	–	–	L‐glutamic acid, spermine, L‐aspartic acid, spermidine
Stoichiometry	1 K^+^ : 1 Cl^‐^ (out)	1 K^+^ : 1 Cl^‐^ (out)	1 K^+^ : 1 Cl^‐^ (out)	Unknown
Inhibitors	VU0240551 (pIC_50_ 6.2) [114], DIOA	DIOA	DIOA	–

### Comments

DIOA is able to differentiate KCC isoforms from NKCC and NCC transporters, but also inhibits CFTR [250].

### Further Reading

Arroyo JP et al. (2013) The SLC12 family of electroneutral cation‐coupled chloride cotransporters. Mol. Aspects Med. 34: 288‐98 [PMID:23506871]

Castrop H et al. (2014) Physiology and pathophysiology of the renal Na‐K‐2Cl cotransporter (NKCC2). Am. J. Physiol. Renal Physiol. 307: F991‐F1002 [PMID:25186299]

Gagnon KB et al. (2013) Physiology of SLC12 transporters: lessons from inherited human genetic mutations and genetically engineered mouse knockouts. Am. J. Physiol., Cell Physiol. 304: C693‐714 [PMID:23325410]

Gamba G et al. (2009) Thick ascending limb: the Na(+):K (+):2Cl (‐) co‐transporter, NKCC2, and the calcium‐sensing receptor, CaSR. Pflugers Arch. 458: 61‐76 [PMID:18982348]

Hebert SC et al. (2004) Molecular physiology of cation‐coupled Cl‐ cotransport: the SLC12 family. Pflugers Arch. 447: 580‐93 [PMID:12739168]

Kahle KT et al. (2015) K‐Cl cotransporters, cell volume homeostasis, and neurological disease. Trends Mol Med [PMID:26142773]

Kahle KT et al. (2010) Phosphoregulation of the Na‐K‐2Cl and K‐Cl cotransporters by the WNK kinases. Biochim. Biophys. Acta 1802: 1150‐8 [PMID:20637866]

Lang F et al. (2007) Functional significance of channels and transporters expressed in the inner ear and kidney. Am. J. Physiol., Cell Physiol. 293: C1187‐208 [PMID:17670895]

Löscher W et al. (2013) Cation‐chloride cotransporters NKCC1 and KCC2 as potential targets for novel antiepileptic and antiepileptogenic treatments. Neuropharmacology 69: 62‐74 [PMID:22705273]

Markadieu N et al. (2014) Physiology and pathophysiology of SLC12A1/2 transporters. Pflugers Arch. 466: 91‐105 [PMID:24097229]

Moes AD et al. (2014) The sodium chloride cotransporter SLC12A3: new roles in sodium, potassium, and blood pressure regulation. Pflugers Arch. 466: 107‐18 [PMID:24310820]

## SLC13 family of sodium‐dependent sulphate/carboxylate transporters

### Overview

Within the SLC13 family, two groups of transporters may be differentiated on the basis of the substrates transported: NaS1 and NaS2 convey sulphate, while NaC1‐3 transport carboxylates. NaS1 and NaS2 transporters are made up of 13 TM domains, with an intracellular N terminus and are electrogenic with physiological roles in the intestine, kidney and placenta. NaC1, NaC2 and NaC3 are made up of 11 TM domains with an intracellular N terminus and are electrogenic, with physiological roles in the kidney and liver.

**Table:** 

Nomenclature	Na^+^/sulfate	Na^+^/dicarboxylate	Na^+^/dicarboxylate	Na^+^/sulfate	Na^+^/citrate
	cotransporter	cotransporter 1	cotransporter 3	cotransporter	cotransporter
Systematic nomenclature	SLC13A1	SLC13A2	SLC13A3	SLC13A4	SLC13A5
Common abreviation	NaS1	NaC1	NaC3	NaS2	NaC2
HGNC, UniProt	SLC13A1, Q9BZW2	SLC13A2, Q13183	SLC13A3, Q8WWT9	SLC13A4, Q9UKG4	SLC13A5, Q86YT5
Endogenous substrates	SeO_4_^2‐^, SO_4_^2‐^, S_2_O_3_^2‐^	citric acid, succinic acid	citric acid, succinic acid	SO_4_^2‐^	citric acid, pyruvic acid
Stoichiometry	3 Na^+^ : 1 SO_4_^2‐^ (in)	3 Na^+^ : 1 dicarboxylate^2‐^ (in)	Unknown	3 Na^+^ : SO_4_^2‐^ (in)	Unknown

### Further Reading

Markovich D. (2014) Na+‐sulfate cotransporter SLC13A1. Pflugers Arch. 466: 131‐7 [PMID:24193406]

Pajor AM. (2014) Sodium‐coupled dicarboxylate and citrate transporters from the SLC13 family. Pflugers Arch. 466: 119‐30 [PMID:24114175]

## SLC14 family of facilitative urea transporters

### Overview

As a product of protein catabolism, urea is moved around the body and through the kidneys for excretion. Although there is experimental evidence for concentrative urea transporters, these have not been defined at the molecular level. The SLC14 family are facilitative transporters, allowing urea movement down its concentration gradient. Multiple splice variants of these transporters have been identified; for UT‐A transporters, in particular, there is evidence for cell‐specific expression of these variants with functional impact [455]. Topographical modelling suggests that the majority of the variants of SLC14 transporters have 10 TM domains, with a glycosylated extracellular loop at TM5/6, and intracellular C‐ and N‐termini. The UT‐A1 splice variant, exceptionally, has 20 TM domains, equivalent to a combination of the UT‐A2 and UT‐A3 splice variants.

**Table:** 

Nomenclature	Erythrocyte urea transporter	Kidney urea transporter
Systematic nomenclature	SLC14A1	SLC14A2
Common abreviation	UT‐B	UT‐A
HGNC, UniProt	SLC14A1, Q13336	SLC14A2, Q15849
Substrates	acetamide [546], acrylamide [546], methylurea [546]	–
Endogenous substrates	ammonium carbonate [546], urea [546], formamide [546]	urea [328]
Stoichiometry	Equilibrative	Equilibrative

### Further Reading

Esteva‐Font C et al. (2015) Urea transporter proteins as targets for small‐molecule diuretics. Nat Rev Nephrol 11: 113‐23 [PMID:25488859]

LeMoine CM et al. (2015) Evolution of urea transporters in vertebrates: adaptation to urea's multiple roles and metabolic sources. J. Exp. Biol. 218: 1936‐1945 [PMID:26085670]

Pannabecker TL. (2013) Comparative physiology and architecture associated with the mammalian urine concentrating mechanism: role of inner medullary water and urea transport pathways in the rodent medulla. Am. J. Physiol. Regul. Integr. Comp. Physiol. 304: R488‐503 [PMID:23364530]

Shayakul C et al. (2013) The urea transporter family (SLC14): physiological, pathological and structural aspects. Mol. Aspects Med. 34: 313‐22 [PMID:23506873]

Shayakul C et al. (2004) The SLC14 gene family of urea transporters. Pflugers Arch. 447: 603‐9 [PMID:12856182]

Smith CP. (2009) Mammalian urea transporters. Exp. Physiol. 94: 180‐5 [PMID:19028811]

Stewart G. (2011) The emerging physiological roles of the SLC14A family of urea transporters. Br. J. Pharmacol. 164: 1780‐92 [PMID:21449978]

## SLC15 family of peptide transporters

### Overview

The SLC15 family of peptide transporters may be divided on the basis of structural and functional differences into two subfamilies: SLC15A1 (PepT1) and SLC15A2 (PepT2) transport di‐ and tripeptides, but not amino acids, whereas SLC15A3 (PHT2) and SLC15A4 (PHT1) transport L‐histidine and some di‐ and tripeptides [105]. The transporters are 12 TM proteins with intracellular termini and an extended extracellular loop at TM 9/10. The crystal structure of PepTSo (a prokaryote homologue of PepT1 and PepT2 from Shewanella oneidensis) confirms many of the predicted structural features of mammalian PepT1 and PepT2 [360].

PHT1 has been suggested to be intracellular [410], while PHT2 protein is located on lysosomes in transfected cells [52, 230, 426]. PHT1 is hypothesised to mediate efflux of bacterial‐derived peptides into the cytosol perhaps in the colon where SLC15A4 mRNA expression is increased in inflammatory bowel disease [305]. Transport via PHT1 may be important in immune responses as both Toll‐like receptor‐ and NOD1‐mediated responses are reduced in PHT1 knockout mice or mouse strains expressing mutations in PHT1 [45, 430].

**Table:** 

Nomenclature	Peptide transporter 1	Peptide transporter 2	Peptide transporter 3	Peptide transporter 4
Systematic nomenclature	SLC15A1	SLC15A2	SLC15A3	SLC15A4
Common abreviation	PepT1	PepT2	PHT2	PHT1
HGNC, UniProt	SLC15A1, P46059	SLC15A2, Q16348	SLC15A3, Q8IY34	SLC15A4, Q8N697
Substrates	fMet‐Leu‐Phe [337], cefadroxil [177], valacyclovir [178], cyclacillin [177], muramyl dipeptide [496]	cefadroxil [177], cyclacillin [177]	–	valacyclovir [37]
Endogenous substrates	dipeptides [135], 5‐aminolevulinic acid [135], tripeptides [135]	dipeptides, 5‐aminolevulinic acid, tripeptides	L‐histidine, carnosine, dipeptides, tripeptides	carnosine, L‐histidine, dipeptides, tripeptides
Stoichiometry	2 H^+^ : 1 zwitterionic peptide (in)	2 H^+^ : 1 zwitterionic peptide (in)	Unknown	Unknown
Inhibitors	Lys[Z(NO_2_)]‐Pro (p*K*_i_ 5) [283], 4‐AMBA [107]	Lys[Z(NO_2_)]‐Lys[Z(NO_2_)] [40, 472], Lys[Z(NO_2_)]‐Pro	–	–
Labelled ligands	[^11^C]GlySar, [^14^C]GlySar, [^3^H]GlySar	[^11^C]GlySar, [^14^C]GlySar, [^3^H]GlySar	[^14^C]histidine, [^3^H]histidine	[^14^C]histidine (Binding) [528], [^3^H]histidine

### Comments

The PepT1 and PepT2 transporters are particularly promiscuous in the transport of dipeptides and tripeptides from the endogenous amino acids, as well as some D‐amino acid containing peptides. PepT1 has also been exploited to allow delivery of therapeutic pro‐drugs, such as those for zidovudine (zidovudine) [218], sulpiride[508] and cytarabine[457].

D‐Ala‐Lys‐AMCA has been used as a fluorescent probe to identify transport via both PepT1 and PepT2 [416] .

### Further Reading

Anderson CM et al. (2010) Hijacking solute carriers for proton‐coupled drug transport. Physiology (Bethesda) 25: 364‐77 [PMID:21186281]

Biegel A et al. (2006) The renal type H+/peptide symporter PEPT2: structure‐affinity relationships. Amino Acids 31: 137‐56 [PMID:16868651]

Brandsch M. (2009) Transport of drugs by proton‐coupled peptide transporters: pearls and pitfalls. Expert Opin Drug Metab Toxicol 5: 887‐905 [PMID:19519280]

Ingersoll SA et al. (2012) The role and pathophysiological relevance of membrane transporter PepT1 in intestinal inflammation and inflammatory bowel disease. Am. J. Physiol. Gastrointest. Liver Physiol. 302: G484‐92 [PMID:22194420]

Newstead S. (2015) Molecular insights into proton coupled peptide transport in the PTR family of oligopeptide transporters. Biochim. Biophys. Acta 1850: 488‐499 [PMID:24859687]

Smith DE et al. (2013) Proton‐coupled oligopeptide transporter family SLC15: physiological, pharmacological and pathological implications. Mol. Aspects Med. 34: 323‐36 [PMID:23506874]

Thwaites DT et al. (2007) H+‐coupled nutrient, micronutrient and drug transporters in the mammalian small intestine. Exp. Physiol. 92: 603‐19 [PMID:17468205]

## SLC16 family of monocarboxylate transporters

### Overview

Members of the SLC16 family may be divided into subfamilies on the basis of substrate selectivities, particularly lactate (e.g. L‐lactic acid), pyruvic acid and ketone bodies, as well as aromatic amino acids. Topology modelling suggests 12 TM domains, with intracellular termini and an extended loop at TM 6/7.

The proton‐coupled monocarboxylate transporters (monocarboxylate transporters 1, 4, 2 and 3) allow transport of the products of cellular metabolism, principally lactate (e.g. L‐lactic acid) and pyruvic acid.

**Table:** 

Nomenclature	Monocarboxylate transporter 1	Monocarboxylate transporter 2	Monocarboxylate transporter 3	Monocarboxylate transporter 4
Systematic nomenclature	SLC16A1	SLC16A7	SLC16A8	SLC16A3
Common abreviation	MCT1	MCT2	MCT3	MCT4
HGNC, UniProt	SLC16A1, P53985	SLC16A7, O60669	SLC16A8, O95907	SLC16A3, O15427
Substrates	*γ*‐hydroxybutyric acid [506]	–	–	–
Endogenous substrates	pyruvic acid, L‐lactic acid, *β*‐D‐hydroxybutyric acid	pyruvic acid, L‐lactic acid	L‐lactic acid	pyruvic acid, L‐lactic acid
Stoichiometry	1 H^+^ : 1 monocarboxylate^‐^ (out)	1 H^+^ : 1 monocarboxylate^‐^ (out)	1 H^+^ : 1 monocarboxylate^‐^ (out)	1 H^+^ : 1 monocarboxylate^‐^ (out)

**Table:** 

Nomenclature	Monocarboxylate transporter 6	Monocarboxylate transporter 8	Monocarboxylate transporter 10
Systematic nomenclature	SLC16A5	SLC16A2	SLC16A10
Common abreviation	MCT6	MCT8	TAT1
HGNC, UniProt	SLC16A5, O15375	SLC16A2, P36021	SLC16A10, Q8TF71
Endogenous substrates	–	triiodothyronine [169], T_4_ [169]	L‐tryptophan, L‐phenylalanine, levodopa, L‐tyrosine
Stoichiometry	Unknown	Unknown	Unknown
Comments	MCT6 has been reported to transport bumetanide, but not short chain fatty acids [353].	–	–

### Comments

MCT1 and MCT2, but not MCT3 and MCT4, are inhibited by CHC, which also inhibits members of the mitochondrial transporter family, SLC25.

MCT5‐MCT7, MCT9 and MCT11‐14 are regarded as orphan transporters.

### Further Reading

Bernal J et al. (2015) Thyroid hormone transporters‐functions and clinical implications. Nat Rev Endocrinol 11: 406‐417 [PMID:25942657]

## SLC17 phosphate and organic anion transporter family

### Overview

The SLC17 family are sometimes referred to as Type I sodium‐phosphate co‐transporters, alongside Type II (SLC34 family) and Type III (SLC20 family) transporters. Within the SLC17 family, however, further subgroups of organic anion transporters may be defined, allowing the accumulation of sialic acid in the endoplasmic reticulum and glutamate (e.g. L‐glutamic acid) or nucleotides in synaptic and secretory vesicles. Topology modelling suggests 12 TM domains.

## Type I sodium‐phosphate co‐transporters

### Overview

Type I sodium‐phosphate co‐transporters are expressed in the kidney and intestine.

**Table:** 

Nomenclature	Sodium/phosphate	Sodium/phosphate	Sodium/phosphate	Sodium/phosphate
	cotransporter 1	cotransporter 3	cotransporter 4	cotransporter homolog
Systematic nomenclature	SLC17A1	SLC17A2	SLC17A3	SLC17A4
Common abreviation	NPT1	NPT3	NPT4	–
HGNC, UniProt	SLC17A1, Q14916	SLC17A2, O00624	SLC17A3, O00476	SLC17A4, Q9Y2C5
Substrates	probenecid [69], penicillin G [69], Cl^‐^ [240], organic acids [240], uric acid [240], phosphate [240]	–	–	–
Stoichiometry	Unknown	Unknown	Unknown	Unknown

## Sialic acid transporter

### Overview

The sialic acid transporter is expressed on both lysosomes and synaptic vesicles, where it appears to allow export of sialic acid and accumulation of acidic amino acids, respectively [349], driven by proton gradients. In lysosomes, degradation of glycoproteins generates amino acids and sugar residues, which are metabolized further following export from the lysosome.

**Table:** 

Nomenclature	Sialin
Systematic nomenclature	SLC17A5
Common abreviation	AST
HGNC, UniProt	SLC17A5, Q9NRA2
Endogenous substrates	L‐lactic acid, gluconate (out), L‐glutamic acid (in) [349], glucuronic acid, L‐aspartic acid [349], sialic acid
Stoichiometry	1 H^+^ : 1 sialic acid (out)

### Comments

Loss‐of‐function mutations in sialin are associated with Salla disease (OMIM: 604369), an autosomal recessive neurodegenerative disorder associated with sialic acid storage disease [497].

## Vesicular glutamate transporters (VGLUTs)

### Overview

Vesicular glutamate transporters (VGLUTs) allow accumulation of glutamate into synaptic vesicles, as well as secretory vesicles in endocrine tissues. The roles of VGLUTs in kidney and liver are unclear. These transporters appear to utilize the proton gradient and also express a chloride conductance [33].

**Table:** 

Nomenclature	Vesicular glutamate transporter 1	Vesicular glutamate transporter 2	Vesicular glutamate transporter 3
Systematic nomenclature	SLC17A7	SLC17A6	SLC17A8
Common abreviation	VGLUT1	VGLUT2	VGLUT3
HGNC, UniProt	SLC17A7, Q9P2U7	SLC17A6, Q9P2U8	SLC17A8, Q8NDX2
Endogenous substrates	L‐glutamic acid>D‐glutamic acid	L‐glutamic acid>D‐glutamic acid	L‐glutamic acid>D‐glutamic acid
Stoichiometry	Unknown	Unknown	Unknown

### Comments

Endogenous ketoacids produced during fasting have been proposed to regulate VGLUT function through blocking chloride ion‐mediated allosteric enhancement of transporter function [258].

## Vesicular nucleotide transporter

### Overview

The vesicular nucleotide transporter is the most recent member of the SLC17 family to have an assigned function. Uptake of ATP was independent of pH, but dependent on chloride ions and membrane potential [431].

**Table:** 

Nomenclature	Vesicular nucleotide transporter
Systematic nomenclature	SLC17A9
Common abreviation	VNUT
HGNC, UniProt	SLC17A9, Q9BYT1
Endogenous substrates	guanosine 5'‐diphosphate [431], guanosine‐5'‐triphosphate [431], ATP [431]
Stoichiometry	Unknown

### Comments

VGLUTs and VNUT can be inhibited by DIDS and evans blue dye.

### Further Reading

Biber J et al. (2013) Phosphate transporters and their function. Annu. Rev. Physiol. 75: 535‐50 [PMID:23398154]

El Mestikawy S et al. (2011) From glutamate co‐release to vesicular synergy: vesicular glutamate transporters. Nat. Rev. Neurosci. 12: 204‐16 [PMID:21415847]

Marks J et al. (2010) Phosphate homeostasis and the renal‐gastrointestinal axis. Am. J. Physiol. Renal Physiol. 299: F285‐96 [PMID:20534868]

Miyamoto K et al. (2011) Sodium‐dependent phosphate cotransporters: lessons from gene knockout and mutation studies. J Pharm Sci 100: 3719‐30 [PMID:21567407]

Omote H et al. (2011) Vesicular neurotransmitter transporter: bioenergetics and regulation of glutamate transport. Biochemistry 50: 5558‐65 [PMID:21612282]

Reimer RJ. (2013) SLC17: a functionally diverse family of organic anion transporters. Mol. Aspects Med. 34: 350‐9 [PMID:23506876]

Shobeiri N et al. (2013) Phosphate: an old bone molecule but new cardiovascular risk factor. Br J Clin Pharmacol [PMID:23506202]

## SLC18 family of vesicular amine transporters

### Overview

The vesicular amine transporters (VATs) are putative 12 TM domain proteins that function to transport singly positively charged amine neurotransmitters and hormones from the cytoplasm and concentrate them within secretory vesicles. They function as amine/proton antiporters driven by secondary active transport utilizing the proton gradient established by a multi‐subunit vacuolar ATPase that acidifies secretory vesicles (reviewed by [139]). The vesicular acetylcholine transporter (VAChT; [148]) localizes to cholinergic neurons, but non‐neuronal expression has also been claimed [434]. Vesicular monoamine transporter 1 (VMAT1, [146]) is mainly expressed in peripheral neuroendocrine cells, but most likely not in the CNS, whereas VMAT2 [147] distributes between both central and peripheral sympathetic monoaminergic neurones [140].

**Table:** 

Nomenclature	Vesicular monoamine	Vesicular monoamine	Vesicular acetylcholine	solute carrier family 18,
	transporter 1	transporter 2	transporter	subfamily B, member 1
Systematic nomenclature	SLC18A1	SLC18A2	SLC18A3	SLC18B1
Common abreviation	VMAT1	VMAT2	VAChT	–
HGNC, UniProt	SLC18A1, P54219	SLC18A2, Q05940	SLC18A3, Q16572	Q6NT16
Substrates	dexamfetamine (*K*_i_4.7 × 10^−5^M) [147], *β*‐phenylethylamine (*K*_i_3.4 × 10^−5^M) [147], fenfluramine (*K*_i_3.1 × 10^−6^M) [147], MPP^+^ (*K*_i_6.9 × 10^−5^M) [147], MDMA (*K*_i_1.9 × 10^−5^M) [147]	*β*‐phenylethylamine (*K*_i_3.7 × 10^−6^M) [147], dexamfetamine (*K*_i_2.1 × 10^−6^M) [147], fenfluramine (*K*_i_5.1 × 10^−6^M) [147], MPP^+^ (*K*_i_8.9 × 10^−6^M) [147], MDMA (*K*_i_6.9 × 10^−6^M) [147]	TPP^+^ [56], ethidium [56], N‐methyl‐pyridinium‐2‐aldoxime [56], N‐(4'‐pentanonyl)‐4‐(4”‐ dimethylamino‐styryl)pyridinium [56]	–
Endogenous substrates	histamine (*K*_i_4.6 × 10^−3^M) [147], 5‐hydroxytryptamine (*K*_i_1.4 × 10^−6^M) [147], dopamine (*K*_i_3.8 × 10^−6^M) [147], (‐)‐noradrenaline (*K*_i_1.3 × 10^−5^M) [147], (‐)‐adrenaline (*K*_i_5.5 × 10^−6^M) [147]	histamine (*K*_i_1.4 × 10^−4^M) [147], dopamine (*K*_i_1.4 × 10^−6^M) [147], 5‐hydroxytryptamine (*K*_i_9 × 10^−7^M) [147], (‐)‐noradrenaline (*K*_i_3.4 × 10^−6^M) [147], (‐)‐adrenaline (*K*_i_1.9 × 10^−6^M) [147]	acetylcholine (*K*_i_7.9 × 10^−4^M) [57, 276], choline (*K*_i_5 × 10^−4^M) [57, 276]	–
Stoichiometry	1 amine (in): 2H^+^ (out)	1 amine (in): 2H^+^ (out)	1 amine (in): 2H^+^ (out)	–
Inhibitors	reserpine (p*K*_i_ 7.5) [147], ketanserin (p*K*_i_ 5.8) [147], tetrabenazine (p*K*_i_ 4.7) [147]	reserpine (p*K*_i_ 7.9) [147], tetrabenazine (p*K*_i_ 7) [147], ketanserin (p*K*_i_ 6.3) [147]	aminobenzovesamicol (p*K*_i_ 10.9) [138], vesamicol (p*K*_i_ 8.7) [138]	–
Labelled ligands	–	[^3^H]TBZOH (Inhibitor) (p*K*_d_ 8.2) [495], [^125^I]iodovinyl‐TBZ (Inhibitor) (p*K*_d_ 8.1) [293], [^11^C]DTBZ (Inhibitor), [^125^I]7‐azido‐8‐iodoketanserine (Inhibitor) [449]	[^3^H]vesamicol (p*K*_d_ 8.4) [495], [^123^I]iodobenzovesamicol	–

### Comments

pK_i_ values for endogenous and synthetic substrate inhibitors of human VMAT1 and VMAT2 are for inhibition of [^3^H]5‐HT uptake in transfected and permeabilised CV‐1 cells as detailed by [147]. In addition to the monoamines listed in the table, the trace amines tyramine and *β*‐phenylethylamine are probable substrates for VMAT2 [140]. Probes listed in the table are those currently employed; additional agents have been synthesized (e.g. [551]).

### Further Reading

Anne C et al. (2014) Vesicular neurotransmitter transporters: mechanistic aspects. Curr Top Membr 73: 149‐74 [PMID:24745982]

Chaudhry FA et al. (2008) Vesicular neurotransmitter transporters as targets for endogenous and exogenous toxic substances. Annu. Rev. Pharmacol. Toxicol. 48: 277‐301 [PMID:17883368]

Eiden LE et al. (2011) VMAT2: a dynamic regulator of brain monoaminergic neuronal function interacting with drugs of abuse. Ann. N. Y. Acad. Sci. 1216: 86‐98 [PMID:21272013]

Giboureau N et al. (2010) PET radioligands for the vesicular acetylcholine transporter (VAChT). Curr Top Med Chem 10: 1569‐83 [PMID:20583990]

Khare P et al. (2010) Equilibrium binding and transport by vesicular acetylcholine transporter. Methods Mol. Biol. 637: 181‐219 [PMID:20419436]

Lawal HO et al. (2013) SLC18: Vesicular neurotransmitter transporters for monoamines and acetylcholine. Mol. Aspects Med. 34: 360‐72 [PMID:23506877]

Prado VF et al. (2013) Regulation of cholinergic activity by the vesicular acetylcholine transporter. Biochem. J. 450: 265‐74 [PMID:23410039]

Ramamoorthy S et al. (2011) Regulation of monoamine transporters: Role of transporter phosphorylation. Pharmacol. Ther. 129: 220‐38 [PMID:20951731]

Wimalasena K. (2011) Vesicular monoamine transporters: structure‐function, pharmacology, and medicinal chemistry. Med Res Rev 31: 483‐519 [PMID:20135628]

## SLC19 family of vitamin transporters

### Overview

The B vitamins folic acid and thiamine are transported across the cell membrane, particularly in the intestine, kidneys and placenta, using pH differences as driving forces. Topological modelling suggests the transporters have 12 TM domains.

**Table:** 

Nomenclature	Reduced folate transporter 1	Thiamine transporter 1	Thiamine transporter 2
Systematic nomenclature	SLC19A1	SLC19A2	SLC19A3
Common abreviation	FOLT	ThTr1	ThTr2
HGNC, UniProt	SLC19A1, P41440	SLC19A2, O60779	SLC19A3, Q9BZV2
Substrates	N^5^‐formyltetrahydrofolate, folinic acid, methotrexate, folic acid [389]	–	–
Endogenous substrates	Other tetrahydrofolate‐cofactors, Organic phosphates; in particular, adenine nucleotides, tetrahydrofolic acid [389], N^5^‐methylfolate [389], thiamine monophosphate [547]	thiamine	thiamine
Stoichiometry	Folate (in) : organic phosphate (out), precise stoichiometry unknown	A facilitative carrier not known to be coupled to an inorganic or organic ion gradient	A facilitative carrier not known to be coupled to an inorganic or organic ion gradient
Labelled ligands	[^3^H]folic acid [19], [^3^H]methotrexate [19]	[^3^H]thiamine [134]	[^3^H]thiamine [399]

### Comments

Loss‐of‐function mutations in ThTr1 underlie thiamine‐responsive megaloblastic anemia syndrome [119].

### Further Reading

Ganapathy V et al. (2004) SLC19: the folate/thiamine transporter family. Pflugers Arch. 447: 641‐6 [PMID:14770311]

Goldman ID et al. (2010) The antifolates: evolution, new agents in the clinic, and how targeting delivery via specific membrane transporters is driving the development of a next generation of folate analogs. Curr Opin Investig Drugs 11: 1409‐23 [PMID:21154123]

Hou Z et al. (2014) Biology of the major facilitative folate transporters SLC19A1 and SLC46A1. Curr Top Membr 73: 175‐204 [PMID:24745983]

Matherly LH et al. (2008) Structure and function of the reduced folate carrier a paradigm of a major facilitator superfamily mammalian nutrient transporter. Vitam. Horm. 79: 145‐84 [PMID:18804694]

Matherly LH et al. (2014) The major facilitative folate transporters solute carrier 19A1 and solute carrier 46A1: biology and role in antifolate chemotherapy of cancer. Drug Metab. Dispos. 42: 632‐49 [PMID:24396145]

Yuasa H et al. (2009) Molecular and functional characteristics of proton‐coupled folate transporter. J Pharm Sci 98: 1608‐16 [PMID:18823045]

Zhao R et al. (2011) Mechanisms of membrane transport of folates into cells and across epithelia. Annu. Rev. Nutr. 31: 177‐201 [PMID:21568705]

Zhao R et al. (2013) Folate and thiamine transporters mediated by facilitative carriers (SLC19A1‐3 and SLC46A1) and folate receptors. Mol. Aspects Med. 34: 373‐85 [PMID:23506878]

## SLC20 family of sodium‐dependent phosphate transporters

### Overview

The SLC20 family is looked upon not only as ion transporters, but also as retroviral receptors. As ion transporters, they are sometimes referred to as Type III sodium‐phosphate co‐transporters, alongside Type I (SLC17 family) and Type II (SLC34 family). PiTs are cell‐surface transporters, composed of ten TM domains with extracellular C‐ and N‐termini. PiT1 is a focus for dietary phosphate and vitamin D regulation of parathyroid hormone secretion from the parathyroid gland. PiT2 appears to be involved in intestinal absorption of dietary phosphate.

**Table:** 

Nomenclature	Sodium‐dependent phosphate transporter 1	Sodium‐dependent phosphate transporter 2
Systematic nomenclature	SLC20A1	SLC20A2
Common abreviation	PiT1	PiT2
HGNC, UniProt	SLC20A1, Q8WUM9	SLC20A2, Q08357
Substrates	AsO_4_^3‐^ [400], phosphate [400]	phosphate [400]
Stoichiometry	>1 Na^+^ : 1 HPO_4_^2‐^ (in)	>1 Na^+^ : 1 HPO_4_^2‐^ (in)

### Further Reading

Biber J et al. (2013) Phosphate transporters and their function. Annu. Rev. Physiol. 75: 535‐50 [PMID:23398154]

Forster IC et al. (2013) Phosphate transporters of the SLC20 and SLC34 families. Mol. Aspects Med. 34: 386‐95 [PMID:23506879]

Marks J et al. (2010) Phosphate homeostasis and the renal‐gastrointestinal axis. Am. J. Physiol. Renal Physiol. 299: F285‐96 [PMID:20534868]

Miyamoto K et al. (2011) Sodium‐dependent phosphate cotransporters: lessons from gene knockout and mutation studies. J Pharm Sci 100: 3719‐30 [PMID:21567407]

Shobeiri N et al. (2013) Phosphate: an old bone molecule but new cardiovascular risk factor. Br J Clin Pharmacol [PMID:23506202]

## SLC22 family of organic cation and anion transporters

### Overview

The SLC22 family of transporters is mostly composed of non‐selective transporters, which are expressed highly in liver, kidney and intestine, playing a major role in drug disposition. The family may be divided into three subfamilies based on the nature of the substrate transported: organic cations (OCTs), organic anions (OATs) and organic zwiterrion/cations (OCTN). Membrane topology is predicted to contain 12 TM domains with intracellular termini, and an extended extracellular loop at TM 1/2.

### Further Reading

Burckhardt G. (2012) Drug transport by Organic Anion Transporters (OATs). Pharmacol. Ther. 136: 106‐30 [PMID:22841915]

Koepsell H. (2013) The SLC22 family with transporters of organic cations, anions and zwitterions. Mol. Aspects Med. 34: 413‐35 [PMID:23506881]

König J et al. (2013) Transporters and drug‐drug interactions: important determinants of drug disposition and effects. Pharmacol. Rev. 65: 944‐66 [PMID:23686349]

Motohashi H et al. (2013) Organic cation transporter OCTs (SLC22) and MATEs (SLC47) in the human kidney. AAPS J 15: 581‐8 [PMID:23435786]

## Organic cation transporters (OCT)

### Overview

Organic cation transporters (OCT) are electrogenic, Na^+^‐independent and reversible.

**Table:** 

Nomenclature	Organic cation transporter 1	Organic cation transporter 2	Organic cation transporter 3
Systematic nomenclature	SLC22A1	SLC22A2	SLC22A3
Common abreviation	OCT1	OCT2	OCT3
HGNC, UniProt	SLC22A1, O15245	SLC22A2, O15244	SLC22A3, O75751
Substrates	MPP^+^, tetraethylammonium, desipramine, metformin, aciclovir	MPP^+^ [198], pancuronium [198], tetraethylammonium [198], tubocurarine [198]	MPP^+^, tetraethylammonium, quinidine
Endogenous substrates	PGF_2*α*_, choline, PGE_2_, 5‐hydroxytryptamine	PGE_2_ [280], dopamine [209], histamine [209]	(‐)‐noradrenaline [550], dopamine [550], 5‐hydroxytryptamine [550]
Stoichiometry	Unknown	Unknown	Unknown

### Comments

corticosterone and quinine are able to inhibit all three organic cation transporters.

### Further Reading

A‐González N et al. (2011) Liver X receptors as regulators of macrophage inflammatory and metabolic pathways. Biochim. Biophys. Acta 1812: 982‐94 [PMID:21193033]

Lozano E et al. (2013) Role of the plasma membrane transporter of organic cations OCT1 and its genetic variants in modern liver pharmacology. Biomed Res Int 2013: 692071 [PMID:23984399]

Pelis RM et al. (2014) SLC22, SLC44, and SLC47 transporters–organic anion and cation transporters: molecular and cellular properties. Curr Top Membr 73: 233‐61 [PMID:24745985]

## Organic zwitterions/cation transporters (OCTN)

### Overview

Organic zwitterions/cation transporters (OCTN) function as organic cation uniporters, organic cation/proton exchangers or sodium/L‐carnitine co‐transporters.

**Table:** 

Nomenclature	Organic cation/carnitine transporter 1	Organic cation/carnitine transporter 2	Carnitine transporter 2
Systematic nomenclature	SLC22A4	SLC22A5	SLC22A16
Common abreviation	OCTN1	OCTN2	CT2
HGNC, UniProt	SLC22A4, Q9H015	SLC22A5, O76082	SLC22A16, Q86VW1
Substrates	verapamil, pyrilamine, tetraethylammonium, MPP^+^	verapamil, tetraethylammonium, MPP^+^, pyrilamine	–
Endogenous substrates	L‐carnitine	L‐carnitine, acetyl‐L‐carnitine	L‐carnitine
Stoichiometry	Unknown	Unknown	Unknown

### Further Reading

Pochini L et al. (2013) OCTN cation transporters in health and disease: role as drug targets and assay development. J Biomol Screen 18: 851‐67 [PMID:23771822]

Tamai I. (2013) Pharmacological and pathophysiological roles of carnitine/organic cation transporters (OCTNs: SLC22A4, SLC22A5 and Slc22a21). Biopharm Drug Dispos 34: 29‐44 [PMID:22952014]

## Organic anion transporters (OATs)

### Overview

Organic anion transporters (OATs) are non‐selective transporters prominent in the kidney and intestine

**Table:** 

Nomenclature	Organic anion transporter 1	Organic anion transporter 2	Organic anion transporter 3
Systematic nomenclature	SLC22A6	SLC22A7	SLC22A8
Common abreviation	OAT1	OAT2	OAT3
HGNC, UniProt	SLC22A6, Q4U2R8	SLC22A7, Q9Y694	SLC22A8, Q8TCC7
Substrates	aminohippuric acid, non‐steroidal anti‐inflammatory drugs	aminohippuric acid, PGE_2_, non‐steroidal anti‐inflammatory drugs	estrone‐3‐sulphate [294], aminohippuric acid [294], cimetidine [294], ochratoxin A [294]
Stoichiometry	Unknown	Unknown	Unknown
Inhibitors	probenecid (Inhibition of urate transport by human SCL22A6.) (pIC_50_ 4.9) [239]	–	–

**Table:** 

Nomenclature	Organic anion transporter 7	Organic anion transporter 5	Organic anion transporter 4
Systematic nomenclature	SLC22A9	SLC22A10	SLC22A11
Common abreviation	OAT4	OAT5	–
HGNC, UniProt	SLC22A9, Q8IVM8	SLC22A10, Q63ZE4	SLC22A11, Q9NSA0
Substrates	–	ochratoxin A [534]	dehydroepiandrosterone sulphate [77], estrone‐3‐sulphate [77], ochratoxin A [77]
Stoichiometry	Unknown	Unknown	Unknown

## Urate transporter

**Table:** 

Nomenclature	Urate anion exchanger 1
Systematic nomenclature	SLC22A12
Common abreviation	URAT1
HGNC, UniProt	SLC22A12, Q96S37
Endogenous substrates	uric acid [145], orotic acid [145]
Stoichiometry	Unknown
Selective inhibitors	sufinpyrazone (pIC_50_ 4) [536]

## SLC23 family of ascorbic acid transporters

### Overview

Predicted to be 12 TM segment proteins, members of this family transport the reduced form of ascorbic acid (while the oxidized form may be handled by members of the SLC2 family (GLUT1/SLC2A1, GLUT3/SLC2A3 and GLUT4/SLC2A4). Phloretin is considered a non‐selective inhibitor of these transporters, with an affinity in the micromolar range.

**Table:** 

Nomenclature	Sodium‐dependent vitamin C	Sodium‐dependent vitamin C	Sodium‐dependent vitamin C	Sodium‐dependent nucleobase
	transporter 1	transporter 2	transporter 3	transporter
Systematic nomenclature	SLC23A1	SLC23A2	SLC23A3	SLC23A4
Common abreviation	SVCT1	SVCT2	SVCT3	SNBT1
HGNC, UniProt	SLC23A1, Q9UHI7	SLC23A2, Q9UGH3	SLC23A3, Q6PIS1	SLC23A4P, –
Endogenous substrates	L‐ascorbic acid>D‐ascorbic acid>dehydroascorbic acid [483]	L‐ascorbic acid>D‐ascorbic acid>dehydroascorbic acid [483]	–	uracil>thymine>guanine, hypoxanthine>xanthine, uridine [526]
Substrates	–	–	–	5‐fluorouracil [526]
Stoichiometry	2 Na^+^: 1 ascorbic acid (in) [483]	2 Na^+^: 1 ascorbic acid (in) [483]	–	1 Na^+^ : 1 uracil (in) [526]
Inhibitors	phloretin (p*K*_i_ 4.2) [483]	–	–	–
Labelled ligands	[^14^C]ascorbic acid (Binding) [326]	[^14^C]ascorbic acid	–	–
Comments	–	–	SLC23A3 does not transport ascorbic acid and remains an orphan transporter.	SLC23A4/SNBT1 is found in rodents and non‐human primates, but the sequence is truncated in the human genome and named as a pseudogene, SLC23A4P

### Further Reading

Bürzle M et al. (2013) The sodium‐dependent ascorbic acid transporter family SLC23. Mol. Aspects Med. 34: 436‐54 [PMID:23506882]

May JM. (2011) The SLC23 family of ascorbate transporters: ensuring that you get and keep your daily dose of vitamin C. Br. J. Pharmacol. 164: 1793‐801 [PMID:21418192]

Savini I et al. (2008) SVCT1 and SVCT2: key proteins for vitamin C uptake. Amino Acids 34: 347‐55 [PMID:17541511]

## SLC24 family of sodium/potassium/calcium exchangers

### Overview

The sodium/potassium/calcium exchange family of transporters utilize the extracellular sodium gradient to drive calcium and potassium co‐transport out of the cell. As is the case for NCX transporters (SLC8A family), NKCX transporters are thought to be bidirectional, with the possibility of calcium influx following depolarization of the plasma membrane. Topological modeling suggests the presence of 10 TM domains, with a large intracellular loop between the fifth and sixth TM regions.

**Table:** 

Nomenclature	Sodium/potassium/calcium exchanger 1	Sodium/potassium/calcium exchanger 6
Systematic nomenclature	SLC24A1	SLC24A6
Common abreviation	NKCX1	NKCX6
HGNC, UniProt	SLC24A1, O60721	SLC8B1, Q6J4K2
Stoichiometry	4Na^+^:(1Ca^2+^ + 1K^+^)	–

### Comments

NKCX6 has been proposed to be the sole member of a CAX Na^+^/Ca^2+^ exchanger family, which may be the mitochondrial transporter responsible for calcium accumulation from the cytosol [441].

### Further Reading

Altimimi HF et al. (2007) Na+/Ca2+‐K+ exchangers (NCKX): functional properties and physiological roles. Channels (Austin) 1: 62‐9 [PMID:18690016]

Schnetkamp PP. (2013) The SLC24 gene family of Na^+^/Ca^2^^+^‐K^+^ exchangers: from sight and smell to memory consolidation and skin pigmentation. Mol. Aspects Med. 34: 455‐64 [PMID:23506883]

Schnetkamp PP et al. (2014) The SLC24 family of K^+^‐dependent Na^+^‐Ca^2^^+^ exchangers: structure‐function relationships. Curr Top Membr 73: 263‐87 [PMID:24745986]

Sekler I. (2015) Standing of giants shoulders the story of the mitochondrial Na(+)Ca(2+) exchanger. Biochem. Biophys. Res. Commun. 460: 50‐2 [PMID:25998733]

## SLC25 family of mitochondrial transporters

### Overview

Mitochondrial transporters are nuclear‐encoded proteins, which convey solutes across the inner mitochondrial membrane. Topological modelling suggests homodimeric transporters, each with six TM segments and termini in the cytosol.

## Mitochondrial di‐ and tri‐carboxylic acid transporter subfamily

### Overview

Mitochondrial di‐ and tri‐carboxylic acid transporters are grouped on the basis of commonality of substrates and include the citrate transporter which facilitates citric acid export from the mitochondria to allow the generation of oxalacetic acid and acetyl CoA through the action of ATP:citrate lyase.

**Table:** 

Nomenclature	Mitochondrial citrate	Mitochondrial dicarboxylate	Mitochondrial oxoglutarate	Mitochondrial oxodicarboxylate
	transporter	transporter	carrier	carrier
Systematic nomenclature	SLC25A1	SLC25A10	SLC25A11	SLC25A21
Common abreviation	CIC	DIC	OGC	ODC
HGNC, UniProt	SLC25A1, P53007	SLC25A10, Q9UBX3	SLC25A11, Q02978	SLC25A21, Q9BQT8
Substrates	phosphoenolpyruvic acid, malic acid, citric acid	SO_4_^2‐^, phosphate, S_2_O_3_^2‐^, succinic acid, malic acid	*α*‐ketoglutaric acid, malic acid	*α*‐ketoglutaric acid, *α*‐oxoadipic acid
Stoichiometry	Malate^2‐^ (in) : H‐citrate^2‐^ (out)	PO_3_^4‐^ (in) : malate^2‐^ (out)	Malate^2‐^ (in) : oxoglutarate^2‐^ (out)	Oxoadipate (in) : oxoglutarate (out)
Inhibitors	1,2,3‐benzenetricarboxylic acid	–	–	–

## Mitochondrial amino acid transporter subfamily

### Overview

Mitochondrial amino acid transporters can be subdivided on the basis of their substrates. Mitochondrial ornithine transporters play a role in the urea cycle by exchanging cytosolic ornithine (L‐ornithine and D‐ornithine) for mitochondrial citrulline (L‐citrulline and D‐citrulline) in equimolar amounts. Further members of the family include transporters of S‐adenosylmethionine and carnitine.

**Table:** 

Nomenclature	AGC1	AGC2	Mitochondrial glutamate	Mitochondrial glutamate
			carrier 2	carrier 1
Systematic nomenclature	SLC25A12	SLC25A13	SLC25A18	SLC25A22
Common abreviation	–	–	GC2	GC1
HGNC, UniProt	SLC25A12, O75746	SLC25A13, Q9UJS0	SLC25A18, Q9H1K4	SLC25A22, Q9H936
Substrates	L‐glutamic acid, 2‐amino‐3‐sulfinopropanoic acid, L‐aspartic acid	2‐amino‐3‐sulfinopropanoic acid, L‐glutamic acid, L‐aspartic acid	L‐glutamic acid	L‐glutamic acid
Stoichiometry	Aspartate : glutamate H^+^ (bidirectional)	Aspartate : glutamate H^+^ (bidirectional)	Glutamate : H^+^ (bidirectional)	Glutamate : H^+^ (bidirectional)

**Table:** 

Nomenclature	Mitochondrial ornithine transporter 2	Mitochondrial ornithine transporter 1	Carnitine/acylcarnitine carrier
Systematic nomenclature	SLC25A2	SLC25A15	SLC25A20
Common abreviation	ORC2	ORC1	CAC
HGNC, UniProt	SLC25A2, Q9BXI2	SLC25A15, Q9Y619	SLC25A20, O43772
Substrates	L‐citrulline [161], L‐arginine [161], L‐lysine [161], D‐lysine [161], D‐arginine [161], D‐citrulline [161], D‐ornithine [161], L‐ornithine [161], D‐histidine [161], L‐histidine [161]	L‐lysine [161], L‐ornithine [161], L‐citrulline [161], L‐arginine [161]	–
Stoichiometry	1 Ornithine (in) :1 citrulline : 1 H^+^ (out)	1 Ornithine (in) :1 citrulline : 1 H^+^ (out)	–
Comments	–	–	Exchanges cytosolic acylcarnitine for mitochondrial carnitine

### Comments

Both ornithine transporters are inhibited by the polyamine spermine[162]. Loss‐of‐function mutations in these genes are associated with hyperornithinemia‐hyperammonemia‐homocitrullinuria.

## Mitochondrial phosphate transporters

### Overview

Mitochondrial phosphate transporters allow the import of inorganic phosphate for ATP production.

**Table:** 

Nomenclature	Mitochondrial phosphate carrier
Systematic nomenclature	SLC25A3
Common abreviation	PHC
HGNC, UniProt	SLC25A3, Q00325
Stoichiometry	PO_3_^4‐^ (in) : OH^‐^ (out) or PO_3_^4‐^ : H^+^ (in)

## Mitochondrial nucleotide transporter subfamily

### Overview

Mitochondrial nucleotide transporters, defined by structural similarlities, include the adenine nucleotide translocator family (SLC25A4, SLC25A5, SLC25A6 and SLC25A31), which under conditions of aerobic metabolism, allow coupling between mitochondrial oxidative phosphorylation and cytosolic energy consumption by exchanging cytosolic adenosine diphosphate for mitochondrial ATP. Further members of the mitochondrial nucleotide transporter subfamily convey diverse substrates including CoA, although not all members have had substrates identified.

**Table:** 

Nomenclature	Mitochondrial adenine nucleotide	Mitochondrial adenine nucleotide	Mitochondrial adenine nucleotide	Mitochondrial adenine nucleotide
	translocator 1	translocator 2	translocator 3	translocator 4
Systematic nomenclature	SLC25A4	SLC25A5	SLC25A6	SLC25A31
Common abreviation	ANT1	ANT2	ANT3	ANT4
HGNC, UniProt	SLC25A4, P12235	SLC25A5, P05141	SLC25A6, P12236	SLC25A31, Q9H0C2
Stoichiometry	ADP^3‐^ (in) : ATP^4‐^ (out)	ADP^3‐^ (in) : ATP^4‐^ (out)	ADP^3‐^ (in) : ATP^4‐^ (out)	ADP^3‐^ (in) : ATP^4‐^ (out)
Inhibitors	bongkrek acid, carboxyatractyloside	–	–	–

**Table:** 

Nomenclature	Graves disease carrier	Peroxisomal membrane protein	Deoxynucleotide carrier 1	S‐Adenosylmethionine carrier
Systematic nomenclature	SLC25A16	SLC25A17	SLC25A19	SLC25A26
Common abreviation	GDC	PMP34	DNC	SAMC1
HGNC, UniProt	SLC25A16, P16260	SLC25A17, O43808	SLC25A19, Q9HC21	SLC25A26, Q70HW3
Substrates	CoA and congeners	adenosine diphosphate, ATP, adenosine 5'‐monophosphate	Nucleotide Diphosphates (NDPs), Deoxynucleotide Diphosphates (dNDPs), Dideoxynucleotide Triphosphates (ddNTPs), Deoxynucleotide Triphosphates (dNTPs)	S‐adenosyl methionine
Stoichiometry	CoA (in)	ATP (in)	dNDP (in) : ATP (out)	–

**Table:** 

Nomenclature	Mitochondrial phosphate carrier 1	Mitochondrial phosphate carrier 2	Mitochondrial phosphate carrier 3
Systematic nomenclature	SLC25A24	SLC25A23	SLC25A25
Common abreviation	APC1	APC2	APC3
HGNC, UniProt	SLC25A24, Q6NUK1	SLC25A23, Q9BV35	SLC25A25, Q6KCM7

## Mitochondrial uncoupling proteins

### Overview

Mitochondrial uncoupling proteins allow dissipation of the mitochondrial proton gradient associated with thermogenesis and regulation of radical formation.

**Table:** 

Nomenclature	Uncoupling protein 1	Uncoupling protein 2	Uncoupling protein 3
Systematic nomenclature	SLC25A7	SLC25A8	SLC25A9
Common abreviation	UCP1	UCP2	UCP3
HGNC, UniProt	UCP1, P25874	UCP2, P55851	UCP3, P55916
Stoichiometry	H^+^ (in)	H^+^ (in)	H^+^ (in)

**Table:** 

Nomenclature	Uncoupling protein 4	Uncoupling protein 5	KMCP1
Systematic nomenclature	SLC25A27	SLC25A14	SLC25A30
Common abreviation	UCP4	UCP5	–
HGNC, UniProt	SLC25A27, O95847	SLC25A14, O95258	SLC25A30, Q5SVS4
Stoichiometry	H^+^ (in)	H^+^ (in)	–

## Miscellaneous SLC25 mitochondrial transporters

### Overview

Many of the transporters identified below have yet to be assigned functions and are currently regarded as orphans.

### Further Reading

Cioffi F et al. (2009) Uncoupling proteins: a complex journey to function discovery. Biofactors 35: 417‐28 [PMID:19626697]

Clémençon B et al. (2013) The mitochondrial ADP/ATP carrier (SLC25 family): pathological implications of its dysfunction. Mol. Aspects Med. 34: 485‐93 [PMID:23506884]

Gnoni GV et al. (2009) The mitochondrial citrate carrier: metabolic role and regulation of its activity and expression. IUBMB Life 61: 987‐94 [PMID:19787704]

Gutiérrez‐Aguilar M et al. (2013) Physiological and pathological roles of mitochondrial SLC25 carriers. Biochem. J. 454: 371‐86 [PMID:23988125]

Monné M et al. (2014) Antiporters of the mitochondrial carrier family. Curr Top Membr 73: 289‐320 [PMID:24745987]

Palmieri F. (2013) The mitochondrial transporter family SLC25: identification, properties and physiopathology. Mol. Aspects Med. 34: 465‐84 [PMID:23266187]

Palmieri F. (2014) Mitochondrial transporters of the SLC25 family and associated diseases: a review. J. Inherit. Metab. Dis. 37: 565‐75 [PMID:24797559]

Palmieri F. (2004) The mitochondrial transporter family (SLC25): physiological and pathological implications. Pflugers Arch. 447: 689‐709 [PMID:14598172]

Seifert EL et al. (2015) The mitochondrial phosphate carrier: Role in oxidative metabolism, calcium handling and mitochondrial disease. Biochem. Biophys. Res. Commun. 464: 369‐75 [PMID:26091567]

## SLC26 family of anion exchangers

### Overview

Along with the SLC4 family, the SLC26 family acts to allow movement of monovalent and divalent anions across cell membranes. The predicted topology is of 10‐14 TM domains with intracellular C‐ and N‐termini, probably existing as dimers. Within the family, subgroups may be identified on the basis of functional differences, which appear to function as anion exchangers and anion channels (SLC26A7 and SLC26A9).

## Selective sulphate transporters

**Table:** 

Nomenclature	Sat‐1	DTDST
Systematic nomenclature	SLC26A1	SLC26A2
HGNC, UniProt	SLC26A1, Q9H2B4	SLC26A2, P50443
Substrates	SO_4_^2‐^, oxalate	SO_4_^2‐^
Stoichiometry	SO_4_^2‐^ (in) : anion (out)	1 SO_4_^2‐^ (in) : 2 Cl^‐^ (out)

## Chloride/bicarbonate exchangers

**Table:** 

Nomenclature	DRA	Pendrin	PAT‐1
Systematic nomenclature	SLC26A3	SLC26A4	SLC26A6
HGNC, UniProt	SLC26A3, P40879	SLC26A4, O43511	SLC26A6, Q9BXS9
Substrates	Cl^‐^	formate, HCO_3_^‐^, OH^‐^, I^‐^, Cl^‐^	formate, oxalate, SO_4_^2‐^, OH^‐^, Cl^‐^, HCO_3_^‐^, I^‐^
Stoichiometry	2 Cl^‐^ (in) : 1 HCO_3_^‐^ (out) or 2 Cl^‐^ (in) : 1 OH^‐^ (out)	Unknown	1 SO_4_^2‐^ (in) : 2 HCO_3_^‐^ (out) or 1 Cl^‐^ (in) : 2 HCO_3_^‐^ (out)

## Anion channels

**Table:** 

Nomenclature	SLC26A7	SLC26A9
HGNC, UniProt	SLC26A7, Q8TE54	SLC26A9, Q7LBE3
Substrates	NO_3_^‐^≫Cl^‐^ = Br^‐^ = I^‐^> SO_4_^2‐^ = L‐glutamic acid	I^‐^> Br^‐^> NO_3_^‐^>Cl^‐^>L‐glutamic acid
Functional Characteristics	Voltage‐ and time‐independent current, linear I‐V relationship [278]	Voltage‐ and time‐independent current, linear I‐V relationship [127]
Comments	–	SLC26A9 has been suggested to operate in two additional modes as a Cl^‐^‐HCO_3_^‐^ exchanger and as a Na^+^‐anion cotransporter [79].

## Other SLC26 anion exchangers

**Table:** 

Nomenclature	Prestin
Systematic nomenclature	SLC26A5
HGNC, UniProt	SLC26A5, P58743
Substrates	HCO_3_^‐^ [347], Cl^‐^ [347]
Stoichiometry	Unknown
Comments	Prestin has been suggested to function as a molecular motor, rather than a transporter

### Further Reading

Alper SL et al. (2013) The SLC26 gene family of anion transporters and channels. Mol. Aspects Med. 34: 494‐515 [PMID:23506885]

Dorwart MR et al. (2008) The solute carrier 26 family of proteins in epithelial ion transport. Physiology (Bethesda) 23: 104‐14 [PMID:18400693]

Kato A et al. (2011) Regulation of electroneutral NaCl absorption by the small intestine. Annu. Rev. Physiol. 73: 261‐81 [PMID:21054167]

Mount DB et al. (2004) The SLC26 gene family of multifunctional anion exchangers. Pflugers Arch. 447: 710‐21 [PMID:12759755]

Nofziger C et al. (2011) Pendrin function in airway epithelia. Cell. Physiol. Biochem. 28: 571‐8 [PMID:22116372]

Ohana E et al. (2009) Diverse transport modes by the solute carrier 26 family of anion transporters. J. Physiol. (Lond.) 587: 2179‐85 [PMID:19015189]

Soleimani M. (2013) SLC26 Cl(‐)/HCO3(‐) exchangers in the kidney: roles in health and disease. Kidney Int. [PMID:23636174]

## SLC27 family of fatty acid transporters

### Overview

Fatty acid transporter proteins (FATPs) are a family (SLC27) of six transporters (FATP1‐6). They have at least one, and possibly six [312, 432], transmembrane segments, and are predicted on the basis of structural similarities to form dimers. SLC27 members have several structural domains: integral membrane associated domain, peripheral membrane associated domain, FATP signature, intracellular AMP binding motif, dimerization domain, lipocalin motif, and an ER localization domain (identified in FATP4 only) [153, 346, 373]. These transporters are unusual in that they appear to express intrinsic very long‐chain acyl‐CoA synthetase (EC 6.2.1.‐ , EC 6.2.1.7) enzyme activity. Within the cell, these transporters may associate with plasma and peroxisomal membranes. FATP1‐4 and ‐6 transport long‐ and very long‐chain fatty acids, while FATP5 transports long‐chain fatty acids as well as bile acids [344, 432].

**Table:** 

Nomenclature	Fatty acid transport protein 1	Fatty acid transport protein 2	Fatty acid transport protein 3
Systematic nomenclature	SLC27A1	SLC27A2	SLC27A3
Common abreviation	FATP1	FATP2	FATP3
HGNC, UniProt	SLC27A1, Q6PCB7	SLC27A2, O14975	SLC27A3, Q5K4L6
Endogenous substrates	palmitic acid>oleic acid>*γ*‐linolenic acid>octanoic acid [190]; arachidonic acid>palmitic acid>oleic acid>butyric acid [432]	–	–

**Table:** 

Nomenclature	Fatty acid transport protein 4	Fatty acid transport protein 5	Fatty acid transport protein 6
Systematic nomenclature	SLC27A4	SLC27A5	SLC27A6
Common abreviation	FATP4	FATP5	FATP6
HGNC, UniProt	SLC27A4, Q6P1M0	SLC27A5, Q9Y2P5	SLC27A6, Q9Y2P4
Endogenous substrates	palmitic acid , oleic acid>*γ*‐linolenic acid>octanoic acid [190]; palmitic acid>oleic acid>butyric acid, *γ*‐linolenic acid>arachidonic acid [454]	–	palmitic acid>oleic acid>*γ*‐linolenic acid>octanoic acid [190]
Comments	FATP4 is genetically linked to restrictive dermopathy.	–	–

### Comments

Although the stoichiometry of fatty acid transport is unclear, it has been proposed to be facilitated by the coupling of fatty acid transport to conjugation with coenzyme A to form fatty acyl CoA esters. Small molecule inhibitors of FATP2 [314, 429] and FATP4 [43, 549], as well as bile acid inhibitors of FATP5 [549], have been described; analysis of the mechanism of action of some of these inhibitors suggests that transport may be selectively inhibited without altering enzymatic activity of the FATP.

C1‐BODIPY‐C12 accumulation has been used as a non‐selective index of fatty acid transporter activity.

FATP2 has two variants: Variant 1 encodes the full‐length protein, while Variant 2 encodes a shorter isoform missing an internal protein segment. FATP6 also has two variants: Variant 2 encodes the same protein as Variant 1 but has an additional segment in the 5' UTR.

### Further Reading

Anderson CM et al. (2013) SLC27 fatty acid transport proteins. Mol. Aspects Med. 34: 516‐28 [PMID:23506886]

Schwenk RW et al. (2010) Fatty acid transport across the cell membrane: regulation by fatty acid transporters. Prostaglandins Leukot. Essent. Fatty Acids 82: 149‐54 [PMID:20206486]

## SLC28 and SLC29 families of nucleoside transporters

### Overview

Nucleoside transporters are divided into two families, the sodium‐dependent, concentrative solute carrier family 28 (SLC28) and the equilibrative, solute carrier family 29 (SLC29). The endogenous substrates are typically nucleosides, although some family members can also transport nucleobases and organic cations.

## SLC28 family

### Overview

SLC28 family membersappear to have 13 TM segments with cytoplasmic N‐termini and extracellular C‐termini, and function as concentrative nucleoside transporters.

**Table:** 

Nomenclature	Sodium/nucleoside cotransporter 1	Sodium/nucleoside cotransporter 2	Solute carrier family 28 member 3
Systematic nomenclature	SLC28A1	SLC28A2	SLC28A3
Common abreviation	CNT1	CNT2	CNT3
HGNC, UniProt	SLC28A1, O00337	SLC28A2, O43868	SLC28A3, Q9HAS3
Substrates	gemcitabine [90], zalcitabine, zidovudine	cladribine [376], didanosine, vidarabine, fludarabine [298], formycin B [298]	zalcitabine, formycin B, cladribine, 5‐fluorouridine, floxuridine, didanosine, zidovudine, zebularine, gemcitabine
Endogenous substrates	adenosine, uridine, cytidine, thymidine	adenosine, guanosine, inosine, thymidine	adenosine, uridine, guanosine, thymidine, inosine, cytidine
Stoichiometry	1 Na^+^ : 1 nucleoside (in)	1 Na^+^ : 1 nucleoside (in)	2 Na^+^ : 1 nucleoside (in)

### Comments

A further two Na^+^‐dependent (stoichiometry 1 Na^+^: 1 nucleoside (in)) nucleoside transporters have been defined on the basis of substrate and inhibitor selectivity: CNT4 (N4/cit, which transports uridine, thymidine and guanosine) and CNT5 (N5/csg, which transports guanosine and adenosine, and may be inhibited by nitrobenzylmercaptopurine ribonucleoside).

## SLC29 family

### Overview

SLC29 family members appear to be composed of 11 TM segments with cytoplasmic N‐termini and extracellular C‐termini. ENT1, ENT2 and ENT4 are cell‐surface transporters, while ENT3 is intracellular, possibly lysosomal [27]. ENT1‐3 are described as broad‐spectrum equilibrative nucleoside transporters, while ENT4 is primarily a polyspecific organic cation transporter at neutral pH [231]. ENT4 transports adenosine only under acidotic conditions [31].

**Table:** 

Nomenclature	Equilibrative nucleoside	Equilibrative nucleoside	Equilibrative nucleoside	Plasma membrane monoamine
	transporter 1	transporter 2	transporter 3	transporter
Systematic nomenclature	SLC29A1	SLC29A2	SLC29A3	SLC29A4
Common abreviation	ENT1	ENT2	ENT3	PMAT
HGNC, UniProt	SLC29A1, Q99808	SLC29A2, Q14542	SLC29A3, Q9BZD2	SLC29A4, Q7RTT9
Endogenous substrates in order of increasing Km:	adenosine<inosine<uridine<guanosine<cytidine<hypoxanthine<adenine<thymine	–	–	–
Substrates	tubercidin, cytarabine, ribavirin, formycin B, cladribine, 2‐chloroadenosine, gemcitabine, didanosine, zalcitabine, pentostatin, vidarabine, floxuridine	formycin B, 2‐chloroadenosine, cytarabine, tubercidin, cladribine, gemcitabine, vidarabine, zidovudine	zidovudine [27], zalcitabine [27], didanosine [27], fludarabine [27], cordycepin [27], floxuridine [27], cladribine [27], tubercidin [27], zebularine [27]	tetraethylammonium [144], MPP^+^ [144]
Endogenous substrates	adenine [529], cytidine [529], thymidine [529], guanosine [529], thymine [529], hypoxanthine [529], uridine [529], adenosine [529], inosine [529]	adenosine, guanine, thymine, uridine, guanosine, hypoxanthine, inosine, thymidine, cytosine	adenosine [27], inosine [27], uridine [27], thymidine [27], guanosine [27], adenine [27]	histamine [144], tyramine [144], adenosine, 5‐hydroxytryptamine [144], dopamine [144]
Stoichiometry	Equilibrative	Equilibrative	Equilibrative	Equilibrative
Inhibitors	nitrobenzylmercaptopurine ribonucleoside (p*K*_i_ 9.7), draflazine (p*K*_i_ 9.6) [216], KF24345 (p*K*_i_ 9.4) [217], NBTGR (p*K*_i_ 9.3), dilazep (p*K*_i_ 9), dipyridamole (p*K*_i_ 8.8) [217]	–	–	decynium 22 (p*K*_i_ 7) [144], rhodamine123 (p*K*_i_ 6) [144], dipyridamole (p*K*_i_ 5.9) [503], verapamil (p*K*_i_ 4.7) [144], fluoxetine (p*K*_i_ 4.6) [144], quinidine (p*K*_i_ 4.6) [144], quinine (p*K*_i_ 4.6) [144], desipramine (p*K*_i_ 4.5) [144], cimetidine (p*K*_i_<3.3) [144]
Labelled ligands	[^3^H]nitrobenzylmercaptopurine ribonucleoside (p*K*_d_ 9.3)	–	–	–
Comments	ENT1 has 100‐1000‐fold lower affinity for nucleobases as compared with nucleosides [529]. The affinities of draflazine, dilazep, KF24345 and dipyridamole at ENT1 transporters are species dependent, exhibiting lower affinity at rat transporters than at human transporters [217, 458]. The loss of ENT1 activity in ENT1‐null mice has been associated with a hypermineralization disorder similar to human diffuse idiopathic skeletal hyperostosis [507]. Lack of ENT1 also results in the Augustine‐null blood type [106].	–	Defects in SLC29A3 have been implicated in Histiocytosis‐lymphadenopathy plus syndrome (OMIM:602782) and lysosomal storage diseases [233, 268].	–

### Further Reading

Cano‐Soldado P et al. (2012) Transporters that translocate nucleosides and structural similar drugs: structural requirements for substrate recognition. Med Res Rev 32: 428‐57 [PMID:21287570]

Dos Santos‐Rodrigues A et al. (2014) Nucleoside transporters in the purinome. Neurochem. Int. 73: 229‐37 [PMID:24704797]

King AE et al. (2006) Nucleoside transporters: from scavengers to novel therapeutic targets. Trends Pharmacol. Sci. 27: 416‐25 [PMID:16820221]

Pastor‐Anglada M et al. (2008) SLC28 genes and concentrative nucleoside transporter (CNT) proteins. Xenobiotica 38: 972‐94 [PMID:18668436]

Young JD et al. (2013) The human concentrative and equilibrative nucleoside transporter families, SLC28 and SLC29. Mol. Aspects Med. 34: 529‐47 [PMID:23506887]

## SLC30 zinc transporter family

### Overview

Along with the SLC39 family, SLC30 transporters regulate the movement of zinc ions around the cell. In particular, these transporters remove zinc ions from the cytosol, allowing accumulation into intracellular compartments or efflux through the plasma membrane. ZnT1 is thought to be placed on the plasma membrane extruding zinc, while ZnT3 is associated with synaptic vesicles and ZnT4 and ZnT5 are linked with secretory granules. Membrane topology predictions suggest a multimeric assembly, potentially heteromultimeric [461], with subunits having six TM domains, and both termini being cytoplasmic. Dityrosine covalent linking has been suggested as a mechanism for dimerisation, particularly for ZnT3 [427]. The mechanism for zinc transport is unknown.

### Comments

ZnT8/SLC30A8 is described as a type 1 diabetes susceptibility gene.

Zinc fluxes may be monitored through the use of radioisotopic Zn‐65 or the fluorescent dye FluoZin 3.

### Further Reading

Bouron A et al. (2013) Contribution of calcium‐conducting channels to the transport of zinc ions. Pflugers Arch. [PMID:23719866]

Huang L et al. (2013) The SLC30 family of zinc transporters ‐ a review of current understanding of their biological and pathophysiological roles. Mol. Aspects Med. 34: 548‐60 [PMID:23506888]

Kambe T et al. (2014) Current understanding of ZIP and ZnT zinc transporters in human health and diseases. Cell. Mol. Life Sci. 71: 3281‐95 [PMID:24710731]

Kambe T et al. (2015) The Physiological, Biochemical, and Molecular Roles of Zinc Transporters in Zinc Homeostasis and Metabolism. Physiol. Rev. 95: 749‐784 [PMID:26084690]

Kawasaki E. (2012) ZnT8 and type 1 diabetes. Endocr. J. 59: 531‐7 [PMID:22447136]

Marger L et al. (2014) Zinc: an underappreciated modulatory factor of brain function. Biochem. Pharmacol. 91: 426‐35 [PMID:25130547]

Palmiter RD et al. (2004) Efflux and compartmentalization of zinc by members of the SLC30 family of solute carriers. Pflugers Arch. 447: 744‐51 [PMID:12748859]

Rungby J. (2010) Zinc, zinc transporters and diabetes. Diabetologia 53: 1549‐51 [PMID:20490449]

Wang X et al. (2010) Dietary zinc absorption: A play of Zips and ZnTs in the gut. IUBMB Life 62: 176‐82 [PMID:20120011]

## SLC31 family of copper transporters

### Overview

SLC31 family members, alongside the Cu‐ATPases are involved in the regulation of cellular copper levels. The CTR1 transporter is a cell‐surface transporter to allow monovalent copper accumulation into cells, while CTR2 appears to be a vacuolar/vesicular transporter [401]. Functional copper transporters appear to be trimeric with each subunit having three TM regions and an extracellular N‐terminus. CTR1 is considered to be a higher affinity copper transporter compared to CTR2. The stoichiometry of copper accumulation is unclear, but appears to be energy‐independent [304].

**Table:** 

Nomenclature	Copper transporter 1	Copper transporter 2
Systematic nomenclature	SLC31A1	SLC31A2
Common abreviation	CTR1	CTR2
HGNC, UniProt	SLC31A1, O15431	SLC31A2, O15432
Substrates	cisplatin [247]	cisplatin [44]
Endogenous substrates	copper [304]	copper
Stoichiometry	Unknown	Unknown

### Comments

Copper accumulation through CTR1 is sensitive to silver ions, but not divalent cations [304].

### Further Reading

Howell SB et al. (2010) Copper transporters and the cellular pharmacology of the platinum‐containing cancer drugs. Mol. Pharmacol. 77: 887‐94 [PMID:20159940]

Kim H et al. (2013) SLC31 (CTR) family of copper transporters in health and disease. Mol. Aspects Med. 34: 561‐70 [PMID:23506889]

Monné M et al. (2014) Antiporters of the mitochondrial carrier family. Curr Top Membr 73: 289‐320 [PMID:24745987]

Nose Y et al. (2006) Structure of the Ctr1 copper trans'PORE'ter reveals novel architecture. Trends Biochem. Sci. 31: 604‐7 [PMID:16982196]

Petris MJ. (2004) The SLC31 (Ctr) copper transporter family. Pflugers Arch. 447: 752‐5 [PMID:12827356]

Zheng W et al. (2012) Regulation of brain iron and copper homeostasis by brain barrier systems: implication in neurodegenerative diseases. Pharmacol. Ther. 133: 177‐88 [PMID:22115751]

## SLC32 vesicular inhibitory amino acid transporter

### Overview

The vesicular inhibitory amino acid transporter, VIAAT (also termed the vesicular GABA transporter VGAT), which is the sole representative of the SLC32 family, transports GABA, or glycine, into synaptic vesicles [182, 183], and is a member of the structurally‐defined amino acid‐polyamine‐organocation/APC clan composed of SLC32, SLC36 and SLC38 transporter families (see [435]). VIAAT was originally suggested to be composed of 10 TM segments with cytoplasmic N‐ and C‐termini [335]. However, an alternative 9TM structure with the N terminus facing the cytoplasm and the C terminus residing in the synaptic vesicle lumen has subsequently been reported [333]. VIAAT acts as an antiporter for inhibitory amino acids and protons. The accumulation of GABA and glycine within vesicles is driven by both the chemical (*Δ*pH) and electrical (*Δ* *ψ*) components of the proton electrochemical gradient (*Δ* *μ*_H_+) established by a vacuolar H^+^‐ATPase [335]. However, one study, [259], presented evidence that VIAAT is instead a Cl^‐^/GABA co‐transporter. VIAAT co‐exists with VGLUT1(SLC17A7), or VGLUT2(SLC17A6), in the synaptic vesicles of selected nerve terminals [155, 539]. VIAAT knock out mice die between embryonic day 18.5 and birth [515]. In cultures of spinal cord neurones established from earlier embryos, the co‐release of of GABA and glycine from synaptic vesicles is drastically reduced, providing direct evidence for the role of VIAAT in the sequestration of both transmitters [425, 515].

**Table:** 

Nomenclature	Vesicular inhibitory amino acid transporter
Systematic nomenclature	SLC32A1
Common abreviation	VIAAT
HGNC, UniProt	SLC32A1, Q9H598
Endogenous substrates	*β*‐alanine, *γ*‐hydroxybutyric acid, GABA (*K*_m_5 × 10^−3^M) [335], glycine
Stoichiometry	1 amino acid (in): 1 H^+^ (out) [182] or 1 amino acid: 2Cl^‐^ (in) [259]
Inhibitors	vigabatrin (pIC_50_ 2.1) [335]

### Further Reading

Erickson JD et al. (2006) Activity‐dependent regulation of vesicular glutamate and GABA transporters: a means to scale quantal size. Neurochem. Int. 48: 643‐9 [PMID:16546297]

Gasnier B. (2000) The loading of neurotransmitters into synaptic vesicles. Biochimie 82: 327‐37 [PMID:10865121]

Gasnier B. (2004) The SLC32 transporter, a key protein for the synaptic release of inhibitory amino acids. Pflugers Arch. 447: 756‐9 [PMID:12750892]

Schiöth HB et al. (2013) Evolutionary origin of amino acid transporter families SLC32, SLC36 and SLC38 and physiological, pathological and therapeutic aspects. Mol. Aspects Med. 34: 571‐85 [PMID:23506890]

## SLC33 acetylCoA transporter

### Overview

Acetylation of proteins is a post‐translational modification mediated by specific acetyltransferases, using the donor acetyl CoA. SLC33A1/AT1 is a putative 11 TM transporter present on the endoplasmic reticulum, expressed in all tissues, but particularly abundant in the pancreas [267], which imports cytosolic acetyl CoA into these intracellular organelles.

**Table:** 

Nomenclature	AcetylCoA transporter
Systematic nomenclature	SLC33A1
Common abreviation	ACATN1
HGNC, UniProt	SLC33A1, O00400
Endogenous substrates	acetyl CoA
Stoichiometry	Unknown
Labelled ligands	[^14^C]acetylCoA (Binding)

### Comments

In heterologous expression studies, acetyl CoA transport through AT1 was inhibited by coenzyme A, but not acetic acid, ATP or UDP‐galactose[255]. A loss‐of‐function mutation in ACATN1/SLC33A1 has been associated with spastic paraplegia (SPG42, [317]), although this observation could not be replicated in a subsequent study [436].

### Further Reading

Hirabayashi Y et al. (2004) The acetyl‐CoA transporter family SLC33. Pflugers Arch. 447: 760‐2 [PMID:12739170]

Hirabayashi Y et al. (2013) The acetyl‐CoA transporter family SLC33. Mol. Aspects Med. 34: 586‐9 [PMID:23506891]

## SLC34 family of sodium phosphate co‐transporters

### Overview

The SLC34 family are sometimes referred to as Type II sodium‐phosphate co‐transporters, alongside Type I (SLC17 family) and Type III (SLC20 family) transporters. Topological modelling suggests eight TM domains with C‐ and N‐ termini in the cytoplasm, and a re‐entrant loop at TM7/8. SLC34 family members are expressed on the apical surfaces of epithelia in the intestine and kidneys to regulate body phosphate levels, principally NaPi‐IIa and NaPi‐IIb, respectively. NaPi‐IIa and NaPi‐IIb are electrogenic, while NaPiIIc is electroneutral [10].

**Table:** 

Nomenclature	Sodium phosphate 1	Sodium phosphate 2	Sodium phosphate 3
Systematic nomenclature	SLC34A1	SLC34A2	SLC34A3
Common abreviation	NaPi‐IIa	NaPi‐IIb	NaPi‐IIc
HGNC, UniProt	SLC34A1, Q06495	SLC34A2, O95436	SLC34A3, Q8N130
Stoichiometry	3 Na^+^ : 1 HPO_4_^2‐^ (in) [168]	3 Na^+^ : 1 HPO_4_^2‐^(in) [10]	2 Na^+^ : 1 HPO_4_^2‐^ (in) [10]
Antibodies	–	lifastuzumab vedotin (Binding) [115]	–

### Comments

These transporters can be inhibited by foscarnet, in contrast to type III sodium‐phosphate cotransporters, the SLC20 family.

### Further Reading

Biber J et al. (2013) Phosphate transporters and their function. Annu. Rev. Physiol. 75: 535‐50 [PMID:23398154]

Forster IC et al. (2013) Phosphate transporters of the SLC20 and SLC34 families. Mol. Aspects Med. 34: 386‐95 [PMID:23506879]

Marks J et al. (2010) Phosphate homeostasis and the renal‐gastrointestinal axis. Am. J. Physiol. Renal Physiol. 299: F285‐96 [PMID:20534868]

Miyamoto K et al. (2011) Sodium‐dependent phosphate cotransporters: lessons from gene knockout and mutation studies. J Pharm Sci 100: 3719‐30 [PMID:21567407]

Murer H et al. (2004) The sodium phosphate cotransporter family SLC34. Pflugers Arch. 447: 763‐7 [PMID:12750889]

Shobeiri N et al. (2013) Phosphate: an old bone molecule but new cardiovascular risk factor. Br J Clin Pharmacol [PMID:23506202]

Wagner CA et al. (2014) The SLC34 family of sodium‐dependent phosphate transporters. Pflugers Arch. 466: 139‐53 [PMID:24352629]

## SLC35 family of nucleotide sugar transporters

### Overview

Glycoprotein formation in the Golgi and endoplasmic reticulum relies on the accumulation of nucleotide‐conjugated sugars via the SLC35 family of transporters. These transporters have a predicted topology of 10 TM domains, with cytoplasmic termini, and function as exchangers, swopping nucleoside monophosphates for the corresponding nucleoside diphosphate conjugated sugar. Five subfamilies of transporters have been identified on the basis of sequence similarity, namely SLC35A1, SLC35A2, SLC35A3, SLC35A4 and SLC35A5; SLC35B1, SLC35B2, SLC35B3 and SLC35B4; SLC35C1 and SLC35C2; SLC35D1, SL35D1, SLC35D2 and SLC35D3, and the subfamily of orphan SLC35 transporters, SLC35E1‐4 and SLC35F1‐5.

**Table:** 

Nomenclature	CMP‐sialic acid transporter	UDP‐galactose transporter	UDP‐N‐acetylglucosamine	PAPS transporter 1	PAPS transporter 2
			transporter		
Systematic nomenclature	SLC35A1	SLC35A2	SLC35A3	SLC35B2	SLC35B3
HGNC, UniProt	SLC35A1, P78382	SLC35A2, P78381	SLC35A3, Q9Y2D2	SLC35B2, Q8TB61	SLC35B3, Q9H1N7
Substrates	CMP‐sialic acid [243]	UDP‐galactose [245, 348], UDP N‐acetyl‐glucosamine [245, 348]	UDP N‐acetyl‐glucosamine [246]	A3P5PS [262]	A3P5PS [261]

**Table:** 

Nomenclature	YEA	GDP‐Fucose transporter	UDP‐glucuronic	HFRC1
			acid/UDP-N-acetylgalactosamine	
			dual transporter	
Systematic nomenclature	SLC35B4	SLC35C1	SLC35D1	SLC35D2
HGNC, UniProt	SLC35B4, Q969S0	SLC35C1, Q96A29	SLC35D1, Q9NTN3	SLC35D2, Q76EJ3
Substrates	UDP‐xylose [18], UDP N‐acetyl‐glucosamine [18]	GDP‐fucose [325]	UDP‐N‐acetylgalactosamine [354], UDP‐glucuronic acid [354]	UDP‐N‐acetylgalactosamine [244]

### Further Reading

Ishida N et al. (2004) Molecular physiology and pathology of the nucleotide sugar transporter family (SLC35). Pflugers Arch. 447: 768‐75 [PMID:12759756]

Song Z. (2013) Roles of the nucleotide sugar transporters (SLC35 family) in health and disease. Mol. Aspects Med. 34: 590‐600 [PMID:23506892]

## SLC36 family of proton‐coupled amino acid transporters

### Overview

The SLC36 family of proton‐coupled amino acid transporters (or PAT) is highly expressed in the intestine and kidney, having roles in the disposition of amino acids [474]. PAT1 is found on the gut epithelia luminal surface accumulating dietary amino acids, and additionally in lysosomal membranes where it likely functions as an efflux mechanism for amino acids produced during intralysosomal proteolysis [4, 420]. PAT2 is found at the apical membrane of the kidney proximal tubule [66]. PAT1 and PAT2 are predicted to have 11 TM domains with intracellular N‐termini [48, 420].

**Table:** 

Nomenclature	Proton‐coupled Amino acid	Proton‐coupled Amino acid	Proton‐coupled Amino acid	Proton‐coupled Amino acid
	Transporter 1	Transporter 2	Transporter 3	Transporter 4
Systematic nomenclature	SLC36A1	SLC36A2	SLC36A3	SLC36A4
Common abreviation	PAT1	PAT2	PAT3	PAT4
HGNC, UniProt	SLC36A1, Q7Z2H8	SLC36A2, Q495M3	SLC36A3, Q495N2	SLC36A4, Q6YBV0
Substrates	MeAIB [86], betaine, vigabatrin [1], 5‐aminolevulinic acid, *β*‐guanidinopropionic acid, gaboxadol [299], L‐azetidine‐2‐carboxylate [274], THPO [300]	MeAIB [87], L‐azetidine‐2‐carboxylate [274]	–	–
Endogenous substrates	GABA, L‐alanine, *β*‐alanine, taurine, D‐cysteine, D‐serine, L‐proline, D‐proline, trans‐4‐hydroxy‐proline [338], glycine [338], D‐alanine, sarcosine	L‐alanine, *β*‐alanine, glycine, sarcosine, L‐proline, trans‐4‐hydroxy‐proline	–	L‐tryptophan [384], L‐proline [384]
Stoichiometry	1 H^+^ : 1 amino acid (in)	1 H^+^ : 1 amino acid (in)	Unknown	Unknown
Inhibitors	5‐hydroxy‐L‐tryptophan (p*K*_i_ 3) [339], L‐tryptophan (p*K*_i_ 2.3) [339], indole‐3‐propionic acid (p*K*_i_ 2.3) [339], 5‐hydroxytryptamine (p*K*_i_ 2.2) [339]	5‐hydroxy‐L‐tryptophan (pIC_50_ 2.8) [137], *α*‐methyl‐D,L‐tryptophan (pIC_50_ 2.5) [137]	–	–
Comments	[^3^H] or [^14^C] labelled substrates as listed above are used as probes	[^3^H] or [^14^C] labelled substrates as listed above are used as probes	–	–

### Comments

Both PAT1 and PAT2 can also function as an electroneutral transport system for H^+^ and fatty acids including acetic acid, propanoic acid and butyric acid[164].

Loss‐of‐function mutations in PAT2 lead to iminoglycinuria and hyperglycinuria in man [65].

### Further Reading

Boll M et al. (2004) The SLC36 family: proton‐coupled transporters for the absorption of selected amino acids from extracellular and intracellular proteolysis. Pflugers Arch. 447: 776‐9 [PMID:12748860]

Schiöth HB et al. (2013) Evolutionary origin of amino acid transporter families SLC32, SLC36 and SLC38 and physiological, pathological and therapeutic aspects. Mol. Aspects Med. 34: 571‐85 [PMID:23506890]

Thwaites DT et al. (2011) The SLC36 family of proton‐coupled amino acid transporters and their potential role in drug transport. Br. J. Pharmacol. 164: 1802‐16 [PMID:21501141]

Thwaites DT et al. (2007) Deciphering the mechanisms of intestinal imino (and amino) acid transport: the redemption of SLC36A1. Biochim. Biophys. Acta 1768: 179‐97 [PMID:17123464]

## SLC37 family of phosphosugar/phosphate exchangers

### Overview

The family of sugar‐phosphate exchangers pass particular phosphorylated sugars across intracellular membranes, exchanging for inorganic phosphate. Of the family of sugar phosphate transporters, most information is available on SPX4, the glucose‐6‐phosphate transporter. This is a 10 TM domain protein with cytoplasmic termini and is associated with the endoplasmic reticulum, with tissue‐specific splice variation.

**Table:** 

Nomenclature	Glycerol‐3‐phosphate transporter	Sugar phosphate exchanger 2	Glucose‐6‐phosphate transporter
Systematic nomenclature	SLC37A1	SLC37A2	SLC37A4
Common abreviation	SPX1	SPX2	SPX4
HGNC, UniProt	SLC37A1, P57057	SLC37A2, Q8TED4	SLC37A4, O43826
Endogenous substrates	glycerol 3‐phosphate, glucose 6‐phosphate	glucose 6‐phosphate	glucose 6‐phosphate
Stoichiometry	Glucose 6‐phosphate (in): phosphate (out) [379].	Glucose 6‐phosphate (in): phosphate (out) [379].	Glucose 6‐phosphate (in): phosphate (out) [84].
Comments	–	–	Multiple polymorphisms have been described for the SLC37A4 gene, some of which associate with a glycogen storage disease [6].

### Further Reading

Bartoloni L et al. (2004) The human sugar‐phosphate/phosphate exchanger family SLC37. Pflugers Arch. 447: 780‐3 [PMID:12811562]

Chou JY et al. (2014) The SLC37 family of sugar‐phosphate/phosphate exchangers. Curr Top Membr 73: 357‐82 [PMID:24745989]

Chou JY et al. (2013) The SLC37 family of phosphate‐linked sugar phosphate antiporters. Mol. Aspects Med. 34: 601‐11 [PMID:23506893]

## SLC38 family of sodium‐dependent neutral amino acid transporters

### Overview

The SLC38 family of transporters appears to be responsible for the functionally‐defined system A and system N mechanisms of amino acid transport and are mostly expressed in the CNS. Two distinct subfamilies are identifiable within the SLC38 transporters. SNAT1, SNAT2 and SNAT4 appear to resemble system A transporters in accumulating neutral amino acids under the influence of the sodium gradient. SNAT3 and SNAT5 appear to resemble system N transporters in utilizing proton co‐transport to accumulate amino acids. The predicted membrane topology is of 11 TM domains with an extracellular C‐terminus and intracellular N‐terminus [435].

## System A‐like transporters

**Table:** 

Nomenclature	sodium‐coupled neutral amino	sodium‐coupled neutral amino	sodium‐coupled neutral amino
	acid transporter 1	acid transporter 2	acid transporter 4
Systematic nomenclature	SLC38A1	SLC38A2	SLC38A4
Common abreviation	SNAT1	SNAT2	SNAT4
HGNC, UniProt	SLC38A1, Q9H2H9	SLC38A2, Q96QD8	SLC38A4, Q969I6
Substrates	L‐alanine>L‐serine, L‐glutamine, L‐asparagine, L‐histidine, L‐cysteine, L‐methionine>glycine, L‐threonine, L‐proline, L‐tyrosine, L‐valine [5]	L‐alanine, L‐methionine>L‐asparagine, L‐glutamine, L‐serine, L‐proline, glycine>L‐threonine, L‐leucine, L‐phenylalanine [223]	L‐histidine>L‐arginine, L‐alanine, L‐asparagine, L‐lysine>glycine, L‐glutamine, L‐serine, L‐proline, L‐leucine, L‐phenylalanine [222]
Substrates	MeAIB	MeAIB	MeAIB
Stoichiometry	1 Na^+^ : 1 amino acid (in) [5]	1 Na^+^ : 1 amino acid (in) [223]	1 Na^+^ : 1 neutral amino acid (in) [222]
Labelled ligands	[^14^C]alanine, [^3^H]alanine	[^14^C]alanine, [^3^H]alanine	[^14^C]alanine, [^14^C]glycine, [^3^H]alanine, [^3^H]glycine
Comments	–	–	Transport of cationic amino acids by SNAT4 was sodium‐independent [222].

## System N‐like transporters

**Table:** 

Nomenclature	Sodium‐coupled neutral amino acid transporter 3	Sodium‐coupled neutral amino acid transporter 5
Systematic nomenclature	SLC38A3	SLC38A5
Common abreviation	SNAT3	SNAT5
HGNC, UniProt	SLC38A3, Q99624	SLC38A5, Q8WUX1
Substrates	L‐histidine , L‐glutamine>L‐asparagine, L‐alanine>L‐glutamic acid [157]	L‐asparagine, L‐serine, L‐histidine, L‐glutamine>glycine, L‐alanine [359]
Substrates	MeAIB	MeAIB
Stoichiometry	1 Na^+^ : 1 amino acid (in) : 1 H^+^ (out) [59]	1 Na^+^ : 1 amino acid (in) : 1 H^+^ (out) [359]
Labelled ligands	[^14^C]glutamine, [^3^H]glutamine	[^14^C]histidine, [^3^H]histidine

## Orphan SLC38 transporters

**Table:** 

Nomenclature	Putative sodium‐coupled neutral amino acid transporter 7
Systematic nomenclature	SLC38A7
Common abreviation	SNAT7
HGNC, UniProt	SLC38A7, Q9NVC3
Comments	SNAT7/SLC38A7 has been described to be a system N‐like transporter allowing preferential accumulation of glutamine (e.g. L‐glutamine), histidine (e.g. L‐histidine) and asparagine (e.g. L‐asparagine) [237].

### Further Reading

Bröer S. (2014) The SLC38 family of sodium‐amino acid co‐transporters. Pflugers Arch. 466: 155‐72 [PMID:24193407]

Bröer S et al. (2011) The role of amino acid transporters in inherited and acquired diseases. Biochem. J. 436: 193‐211 [PMID:21568940]

Hägglund MG et al. (2011) Identification of SLC38A7 (SNAT7) protein as a glutamine transporter expressed in neurons. J. Biol. Chem. 286: 20500‐11 [PMID:21511949]

Mackenzie B et al. (2004) Sodium‐coupled neutral amino acid (System N/A) transporters of the SLC38 gene family. Pflugers Arch. 447: 784‐95 [PMID:12845534]

Schiöth HB et al. (2013) Evolutionary origin of amino acid transporter families SLC32, SLC36 and SLC38 and physiological, pathological and therapeutic aspects. Mol. Aspects Med. 34: 571‐85 [PMID:23506890]

Sundberg BE et al. (2008) The evolutionary history and tissue mapping of amino acid transporters belonging to solute carrier families SLC32, SLC36, and SLC38. J. Mol. Neurosci. 35: 179‐93 [PMID:18418736]

## SLC39 family of metal ion transporters

### Overview

Along with the SLC30 family, SLC39 family members regulate zinc movement in cells. SLC39 metal ion transporters accumulate zinc into the cytosol. Membrane topology modelling suggests the presence of eight TM regions with both termini extracellular or in the lumen of intracellular organelles. The mechanism for zinc transport for many members is unknown but appears to involve co‐transport of bicarbonate ions [191, 321].

**Table:** 

Nomenclature	Zinc transporter 8	Zinc transporter 14
Systematic nomenclature	SLC39A8	SLC39A14
Common abreviation	ZIP8	ZIP14
HGNC, UniProt	SLC39A8, Q9C0K1	SLC39A14, Q15043
Substrates	Cd^2+^ [104, 321]	Cd^2+^ [191], Mn^2+^ [191], Fe^2+^ [322]
Stoichiometry	1 Zn^2+^ (in) : 2 HCO_3_^‐^ (in) [321]	–

### Comments

Zinc fluxes may be monitored through the use of radioisotopic Zn‐65 or the fluorescent dye FluoZin 3.

The bicarbonate transport inhibitor DIDS has been reported to inhibit cation accumulation through ZIP14 [191].

### Further Reading

Eide DJ. (2004) The SLC39 family of metal ion transporters. Pflugers Arch. 447: 796‐800 [PMID:12748861]

Franz MC et al. (2013) Zinc transporters in prostate cancer. Mol. Aspects Med. 34: 735‐41 [PMID:23506906]

Himeno S et al. (2009) The role of zinc transporters in cadmium and manganese transport in mammalian cells. Biochimie 91: 1218‐22 [PMID:19375483]

Jeong J et al. (2013) The SLC39 family of zinc transporters. Mol. Aspects Med. 34: 612‐9 [PMID:23506894]

Kambe T et al. (2014) Current understanding of ZIP and ZnT zinc transporters in human health and diseases. Cell. Mol. Life Sci. 71: 3281‐95 [PMID:24710731]

Kambe T et al. (2015) The Physiological, Biochemical, and Molecular Roles of Zinc Transporters in Zinc Homeostasis and Metabolism. Physiol. Rev. 95: 749‐784 [PMID:26084690]

Marger L et al. (2014) Zinc: an underappreciated modulatory factor of brain function. Biochem. Pharmacol. 91: 426‐35 [PMID:25130547]

Rungby J. (2010) Zinc, zinc transporters and diabetes. Diabetologia 53: 1549‐51 [PMID:20490449]

Thévenod F. (2010) Catch me if you can! Novel aspects of cadmium transport in mammalian cells. Biometals 23: 857‐75 [PMID:20204475]

## SLC40 iron transporter

### Overview

Alongside the SLC11 family of proton‐coupled metal transporters, ferroportin allows the accumulation of iron from the diet. Whilst SLC11A2 functions on the apical membrane, ferroportin acts on the basolateral side of the enterocyte, as well as regulating macrophage and placental iron levels. The predicted topology is of 12 TM domains, with intracellular termini [407], with the functional transporter potentially a dimeric arrangement [3, 111]. Ferroportin is essential for iron homeostasis [126]. Ferroportin is expressed on the surface of cells that store and transport iron, such as duodenal enterocytes, hepatocytes, adipocytes and reticuloendothelial macrophages. Levels of ferroportin are regulated by its association with (binding to) hepcidin, a 25 amino acid hormone responsive to circulating iron levels (amongst other signals). Hepcidin binding targets ferroportin for internalisation and degradation, lowering the levels of iron export to the blood. Novel therapeutic agents which stabilise ferroportin or protect it from hepcidin‐induced degradation are being developed as anti‐anemia agents. Anti‐ferroportin monoclonal antibodies are such an agent.

**Table:** 

Nomenclature	Ferroportin
Systematic nomenclature	SLC40A1
Common abreviation	IREG1
HGNC, UniProt	SLC40A1, Q9NP59
Endogenous substrates	Fe^2+^
Stoichiometry	Unknown
Antibodies	LY2928057 (Binding) [311]

### Comments

Hepcidin (HAMP, P81172), cleaved into hepcidin‐25(HAMP, P81172) and hepcidin‐20(HAMP, P81173), is a small protein that increases upon inflammation, binds to ferroportin to regulate its cellular distribution and degradation. Gene disruption in mice results in embryonic lethality [126], while loss‐of‐function mutations in man are associated with haemochromatosis [112].

### Further Reading

McKie AT et al. (2004) The SLC40 basolateral iron transporter family (IREG1/ferroportin/MTP1). Pflugers Arch. 447: 801‐6 [PMID:12836025]

Montalbetti N et al. (2013) Mammalian iron transporters: families SLC11 and SLC40. Mol. Aspects Med. 34: 270‐87 [PMID:23506870]

## SLC41 family of divalent cation transporters

### Overview

By analogy with bacterial orthologues, this family is probably magnesium transporters. The prokaryote orthologue, MgtE, is responsible for uptake of divalent cations, while the heterologous expression studies of mammalian proteins suggest Mg^2+^ efflux [287], possibly as a result of co‐expression of particular protein partners (see [421]). Topological modelling suggests 10 TM domains with cytoplasmic C‐ and N‐ termini.

**Table:** 

Nomenclature	Solute carrier family 41 member 1	Solute carrier family 41 member 2
Systematic nomenclature	SLC41A1	SLC41A2
Common abreviation	MgtE	–
HGNC, UniProt	SLC41A1, Q8IVJ1	SLC41A2, Q96JW4
Substrates	Co^2+^ [201], Cu^2+^ [201], Ba^2+^ [201], Cd^2+^ [201], Zn^2+^ [201], Mg^2+^ [201], Sr^2+^ [201], Fe^2+^ [201]	Ba^2+^ [200], Mg^2+^ [200], Co^2+^ [200], Ni^2+^ [200], Mn^2+^ [200], Fe^2+^ [200]
Stoichiometry	Unknown	Unknown

### Further Reading

Moomaw AS et al. (2008) The unique nature of mg2+ channels. Physiology (Bethesda) 23: 275‐85 [PMID:18927203]

Payandeh J et al. (2013) The structure and regulation of magnesium selective ion channels. Biochim. Biophys. Acta [PMID:23954807]

Quamme GA. (2010) Molecular identification of ancient and modern mammalian magnesium transporters. Am. J. Physiol., Cell Physiol. 298: C407‐29 [PMID:19940067]

Sahni J et al. (2013) The SLC41 family of MgtE‐like magnesium transporters. Mol. Aspects Med. 34: 620‐8 [PMID:23506895]

Schweigel‐Röntgen M et al. (2014) SLC41 transporters–molecular identification and functional role. Curr Top Membr 73: 383‐410 [PMID:24745990]

## SLC42 family of Rhesus glycoprotein ammonium transporters

### Overview

Rhesus is commonly defined as a ‘factor’ that determines, in part, blood type, and whether neonates suffer from haemolytic disease of the newborn. These glycoprotein antigens derive from two genes, RHCE (P18577) and RHD(Q02161), expressed on the surface of erythrocytes. On erythrocytes, RhAG associates with these antigens and functions as an ammonium transporter. RhBG and RhBG are non‐erythroid related sequences associated with epithelia. Topological modelling suggests the presence of 12TM with cytoplasmic N‐ and C‐ termini. The majority of information on these transporters derives from orthologues in yeast, plants and bacteria. More recent evidence points to family members being permeable to carbon dioxide, leading to the term gas channels.

**Table:** 

Nomenclature	Ammonium transporter Rh type A	Ammonium transporter Rh type B	Ammonium transporter Rh type C
Systematic nomenclature	SLC42A1	SLC42A2	SLC42A3
Common abreviation	RhAG	RhBG	RhCG
HGNC, UniProt	RHAG, Q02094	RHBG, Q9H310	RHCG, Q9UBD6
Substrates	NH_4_^+^ [510], NH_3_ [408], CO_2_ [143]	–	NH_3_ [552]
Stoichiometry	Unknown	Unknown	Unknown
Labelled ligands	[^14^C]methylamine (Binding) [228]	–	[^14^C]methylamine (Binding) [330] – Mouse

### Further Reading

Huang CH et al. (2010) The Rh protein family: gene evolution, membrane biology, and disease association. Cell. Mol. Life Sci. 67: 1203‐18 [PMID:19953292]

Nakhoul NL et al. (2004) Non‐erythroid Rh glycoproteins: a putative new family of mammalian ammonium transporters. Pflugers Arch. 447: 807‐12 [PMID:12920597]

Nakhoul NL et al. (2013) Characteristics of mammalian Rh glycoproteins (SLC42 transporters) and their role in acid‐base transport. Mol. Aspects Med. 34: 629‐37 [PMID:23506896]

Weiner ID et al. (2011) Role of NH3 and NH4+ transporters in renal acid‐base transport. Am. J. Physiol. Renal Physiol. 300: F11‐23 [PMID:21048022]

Weiner ID et al. (2014) Ammonia transport in the kidney by Rhesus glycoproteins. Am. J. Physiol. Renal Physiol. 306: F1107‐20 [PMID:24647713]

## SLC43 family of large neutral amino acid transporters

### Overview

LAT3 (SLC43A1) and LAT4 (SLC43A2) are transporters with system L amino acid transporter activity, along with the structurally and functionally distinct transporters LAT1 and LAT2 that are members of the SLC7 family. LAT3 and LAT4 contain 12 putative TM domains with both N and C termini located intracellularly. They transport neutral amino acids in a manner independent of Na^+^ and Cl^‐^ and with two kinetic components [22, 47]. LAT3/SLC43A1 is expressed in human tissues at high levels in the pancreas, liver, skeletal muscle and fetal liver [22] whereas LAT4/SLC43A2 is primarily expressed in the placenta, kidney and peripheral blood leukocytes [47]. SLC43A3 is expressed in vascular endothelial cells [502] but remains to be characterised.

**Table:** 

Nomenclature	L‐type amino acid transporter 3	L‐type amino acid transporter 4
Systematic nomenclature	SLC43A1	SLC43A2
Common abreviation	LAT3	LAT4
HGNC, UniProt	SLC43A1, O75387	SLC43A2, Q8N370
Substrates	L‐isoleucine [22], L‐valinol [22], L‐leucinol [22], L‐phenylalaninol [22], L‐leucine [22], L‐phenylalanine [22], L‐valine [22], L‐methionine [22]	L‐isoleucine, L‐valinol, L‐leucinol, L‐leucine, L‐phenylalanine, L‐valine, L‐methionine
Stoichiometry	Operates by facilitative diffusion	Operates by facilitative diffusion

### Comments

Covalent modification of LAT3 by N‐ethylmaleimide inhibits its function [22] and at LAT4 inhibits the low‐, but not high‐affinity component of transport [47].

### Further Reading

Bodoy S et al. (2013) The small SLC43 family: facilitator system l amino acid transporters and the orphan EEG1. Mol. Aspects Med. 34: 638‐45 [PMID:23268354]

## SLC44 choline transporter‐like family

### Overview

Members of the choline transporter‐like family are encoded by five genes (CTL1‐CTL5) with further diversity occurring through alternative splicing of CTL1, 4 and 5 [477]. CTL family members are putative 10TM domain proteins with extracellular termini that mediate Na^+^‐independent transport of choline with an affinity that is intermediate to that of the high affinity choline transporter CHT1 (SLC5A7) and the low affinity organic‐cation transporters [OCT1 (SLC22A1) and OCT2 (SLC22A2)] [343]. CLT1 is expressed almost ubiquitously in human tissues [514] and mediates choline transport across the plasma and mitochondrial membranes [342]. Transport of choline by CTL2, which in rodents is expressed as two isoforms (CTL2P1 and CLTP2; [288]) in lung, colon, inner ear and spleen and to a lesser extent in brain, tongue, liver, and kidney, has only recently been demonstrated [288, 358]. CTL3‐5 remain to be characterized functionally.

**Table:** 

Nomenclature	Choline transporter‐like 1
Systematic nomenclature	SLC44A1
Common abreviation	CTL1
HGNC, UniProt	SLC44A1, Q8WWI5
Substrates	choline
Stoichiometry	Unknown: uptake enhanced in the absence of extracellular Na^+^, reduced by membrane depolarization, extracellular acidification and collapse of plasma membrane H^+^ electrochemical gradient
Inhibitors	hemicholinium‐3 (p*K*_i_ 3.5–4.5)

### Comments

Data tabulated are features observed for CLT1 endogenous to: rat astrocytes [242]; rat renal tubule epithelial cells [524]; human colon carcinoma cells [289]; human keratinocytes [486] and human neuroblastoma cells [525]. Choline uptake by CLT1 is inhibited by numerous organic cations (e.g. [242, 524, 525]). In the guinea‐pig, CTL2 is a target for antibody‐induced hearing loss [355] and in man, a polymorphism in CTL2 constitutes the human neutrophil alloantigen‐3a (HNA‐3a; [203]).

### Further Reading

Inazu M. (2014) Choline transporter‐like proteins CTLs/SLC44 family as a novel molecular target for cancer therapy. Biopharm Drug Dispos 35: 431‐49 [PMID:24532461]

Lockman PR et al. (2002) The transport of choline. Drug Dev Ind Pharm 28: 749‐71 [PMID:12236062]

Michel V et al. (2006) Choline transport for phospholipid synthesis. Exp. Biol. Med. (Maywood) 231: 490‐504 [PMID:16636297]

Traiffort E et al. (2013) The choline transporter‐like family SLC44: properties and roles in human diseases. Mol. Aspects Med. 34: 646‐54 [PMID:23506897]

## SLC45 family of putative sugar transporters

### Overview

Members of the SLC45 family remain to be fully characterised. SLC45A1 was initially identified in the rat brain, particularly predominant in the hindbrain, as a proton‐associated sugar transport, induced by hypercapnia [447]. The protein is predicted to have 12TM domains, with intracellular termini. The SLC45A2 gene is thought to encode a transporter protein that mediates melanin synthesis. Mutations in SLC45A2 are a cause of oculocutaneous albinism type 4 (e.g. [361]), and polymorphisms in this gene are associated with variations in skin and hair color (e.g. [202]).

**Table:** 

Nomenclature	Proton‐associated sugar transporter A
Systematic nomenclature	SLC45A1
HGNC, UniProt	SLC45A1, Q9Y2W3
Substrates	L‐glucose [447], Galactose [447]
Stoichiometry	Unknown; increased at acid pH [447].

### Further Reading

Bartölke R et al. (2014) Proton‐associated sucrose transport of mammalian solute carrier family 45: an analysis in Saccharomyces cerevisiae. Biochem. J. 464: 193‐201 [PMID:25164149]

Vitavska O et al. (2013) The SLC45 gene family of putative sugar transporters. Mol. Aspects Med. 34: 655‐60 [PMID:23506898]

## SLC46 family of folate transporters

### Overview

Based on the proptypical member of this family, PCFT, this family includes proton‐driven transporters with 11 TM segments. SLC46A1 has been described to act as an intestinal proton‐coupled high‐affinity folic acid transporter [393], with lower affinity for heme. Folic acid accumulation is independent of Na^+^ or K^+^ ion concentrations, but driven by extracellular protons with an as yet undefined stoichiometry.

**Table:** 

Nomenclature	Proton‐coupled folate transporter
Systematic nomenclature	SLC46A1
Common abreviation	PCFT
HGNC, UniProt	SLC46A1, Q96NT5
Substrates	folic acid (1.3*μ*M) >heme (>100 *μ*M) [356]
Substrates	pemetrexed, N‐formyltetrahydrofolate, methotrexate [393]
Endogenous substrates	N^5^‐methyltetrafolate [393]
Labelled ligands	[^3^H]N^5^‐methylfolate (Binding), [^3^H]folic acid, [^3^H]folinic acid (Binding), [^3^H]methotrexate, [^3^H]pemetrexed (Binding)
Comments	Loss‐of‐function mutations in PCFT (SLC46A1) are the molecular basis for hereditary folate maladsorption [428].

### Further Reading

Anderson CM et al. (2010) Hijacking solute carriers for proton‐coupled drug transport. Physiology (Bethesda) 25: 364‐77 [PMID:21186281]

Desmoulin SK et al. (2012) The human proton‐coupled folate transporter: Biology and therapeutic applications to cancer. Cancer Biol. Ther. 13: 1355‐73 [PMID:22954694]

Hou Z et al. (2014) Biology of the major facilitative folate transporters SLC19A1 and SLC46A1. Curr Top Membr 73: 175‐204 [PMID:24745983]

Matherly LH et al. (2014) The major facilitative folate transporters solute carrier 19A1 and solute carrier 46A1: biology and role in antifolate chemotherapy of cancer. Drug Metab. Dispos. 42: 632‐49 [PMID:24396145]

Thwaites DT et al. (2007) H+‐coupled nutrient, micronutrient and drug transporters in the mammalian small intestine. Exp. Physiol. 92: 603‐19 [PMID:17468205]

Wilson MR et al. (2015) Structural determinants of human proton‐coupled folate transporter oligomerization: role of GXXXG motifs and identification of oligomeric interfaces at transmembrane domains 3 and 6. Biochem. J. [PMID:25877470]

Zhao R et al. (2011) Mechanisms of membrane transport of folates into cells and across epithelia. Annu. Rev. Nutr. 31: 177‐201 [PMID:21568705]

Zhao R et al. (2013) Folate and thiamine transporters mediated by facilitative carriers (SLC19A1‐3 and SLC46A1) and folate receptors. Mol. Aspects Med. 34: 373‐85 [PMID:23506878]

## SLC47 family of multidrug and toxin extrusion transporters

### Overview

These proton:organic cation exchangers are predicted to have 13 TM segments [545] and are suggested to be responsible for excretion of many drugs in the liver and kidneys.

**Table:** 

Nomenclature	Multidrug and toxin extrusion	MATE2
Systematic nomenclature	SLC47A1	SLC47A2
Common abreviation	MATE1	MATE2‐K
HGNC, UniProt	SLC47A1, Q96FL8	SLC47A2, Q86VL8
Substrates	quinidine [467], cephradine [467], metformin (*K*_m_7.8 × 10^−4^M) [467], cephalexin [467], cimetidine (*K*_m_1.7 × 10^−4^M) [369, 467], paraquat [85]	guanidine [467], procainamide [334], metformin (*K*_m_1.9 × 10^−3^M) [334, 467], aciclovir [467], MPP^+^ [334], cimetidine (*K*_m_1.2 × 10^−4^M) [334, 467], N^1^‐methylnicotinamide [334]
Endogenous substrates	thiamine [467], creatine [467]	creatine [467], thiamine [467]
(Sub)family‐selective inhibitors	pyrimethamine (p*K*_i_ 7.1) [249], cimetidine (p*K*_i_ 6) [482]	pyrimethamine (p*K*_i_ 6.3) [249] – Mouse, cimetidine (p*K*_i_ 5.1) [482]
Labelled ligands	[^14^C]TEA [374, 469], [^14^C]metformin [467, 469]	[^14^C]TEA [467]

### Comments

DAPI has been used to allow quantification of MATE1 and MATE2‐mediated transport activity [531]. MATE2 and MATE2‐B are inactive splice variants of MATE2‐K [334].

### Further Reading

Damme K et al. (2011) Mammalian MATE (SLC47A) transport proteins: impact on efflux of endogenous substrates and xenobiotics. Drug Metab. Rev. 43: 499‐523 [PMID:21923552]

Motohashi H et al. (2013) Multidrug and toxin extrusion family SLC47: physiological, pharmacokinetic and toxicokinetic importance of MATE1 and MATE2‐K. Mol. Aspects Med. 34: 661‐8 [PMID:23506899]

Terada T et al. (2008) Physiological and pharmacokinetic roles of H+/organic cation antiporters (MATE/SLC47A). Biochem. Pharmacol. 75: 1689‐96 [PMID:18262170]

Yonezawa A et al. (2011) Importance of the multidrug and toxin extrusion MATE/SLC47A family to pharmacokinetics, pharmacodynamics/toxicodynamics and pharmacogenomics. Br. J. Pharmacol. 164: 1817‐25 [PMID:21457222]

## SLC48 heme transporter

### Overview

HRG1 has been identified as a cell surface and lysosomal heme transporter [398]. In addition, evidence suggests this 4TM‐containing protein associates with the V‐ATPase in lysosomes [367]. Recent studies confirm its lysosomal location and demonstrate that it has an important physiological function in macrophages ingesting senescent red blood cells (erythrophagocytosis), recycling heme (released from the red cell hemoglobin) from the phagolysosome into the cytosol, where the heme is subsequently catabolized to recycle the iron [511].

**Table:** 

Nomenclature	Heme transporter
Systematic nomenclature	SLC48A1
Common abreviation	HRG1
HGNC, UniProt	SLC48A1, Q6P1K1

### Further Reading

Khan AA et al. (2013) Heme and FLVCR‐related transporter families SLC48 and SLC49. Mol. Aspects Med. 34: 669‐82 [PMID:23506900]

## SLC49 family of FLVCR‐related heme transporters

### Overview

FLVCR1 was initially identified as a cell‐surface attachment site for feline leukemia virus subgroup C [464], and later identified as a cell surface accumulation which exports heme from the cytosol [395]. A recent study indicates that an isoform of FLVCR1 is located in the mitochondria, the site of the final steps of heme synthesis, and appears to transport heme into the cytosol [89]. FLVCR‐mediated heme transport is essential for erythropoiesis. Flvcr1 gene mutations have been identified as the cause of PCARP (posterior column ataxia with retinitis pigmentosa (PCARP) [397].There are three paralogs of FLVCR1 in the human genome.

FLVCR2, most similar to FLVCR1 [319], has been reported to function as a heme importer [129]. In addition, a congenital syndrome of proliferative vasculopathy and hydranencephaly, also known as Fowler's syndrome, is associated with a loss‐of‐function mutation in FLVCR2 [340].

The functions of the other two members of the SLC49 family, MFSD7 and DIRC2, are unknown, although DIRC2 has been implicated in hereditary renal carcinomas [46].

**Table:** 

Nomenclature	Feline leukemia virus subgroup C cellular receptor family, member 1	Feline leukemia virus subgroup C cellular receptor family, member 2
Systematic nomenclature	SLC49A1	SLC49A2
Common abreviation	FLVCR1	FLVCR2
HGNC, UniProt	FLVCR1, Q9Y5Y0	FLVCR2, Q9UPI3
Substrates	heme [395]	heme [129]
Stoichiometry	Unknown	Unknown

### Comments

Non‐functional splice alternatives of FLVCR1 have been implicated as a cause of a congenital red cell aplasia, Diamond Blackfan anemia[405].

### Further Reading

Khan AA et al. (2013) Heme and FLVCR‐related transporter families SLC48 and SLC49. Mol. Aspects Med. 34: 669‐82 [PMID:23506900]

Khan AA et al. (2011) Control of intracellular heme levels: heme transporters and heme oxygenases. Biochim. Biophys. Acta 1813: 668‐82 [PMID:21238504]

Krishnamurthy P et al. (2007) The role of transporters in cellular heme and porphyrin homeostasis. Pharmacol. Ther. 114: 345‐58 [PMID:17368550]

Latunde‐Dada GO et al. (2006) Recent advances in mammalian haem transport. Trends Biochem. Sci. 31: 182‐8 [PMID:16487711]

## SLC50 sugar transporter

### Overview

A mouse stromal cell cDNA library was used to clone C2.3 [463], later termed Rag1‐activating protein 1, with a sequence homology predictive of a 4TM topology. The plant orthologues, termed SWEETs, appear to be 7 TM proteins, with extracellular N‐termini, and the capacity for bidirectional flux of D‐glucose[82]. Expression of mouse SWEET in the mammary gland was suggestive of a role in Golgi lactose synthesis [82].

**Table:** 

Nomenclature	SLC50 sugar exporter
Systematic nomenclature	SLC50A1
Common abreviation	RAG1AP1
HGNC, UniProt	SLC50A1, Q9BRV3

### Further Reading

Wright EM. (2013) Glucose transport families SLC5 and SLC50. Mol. Aspects Med. 34: 183‐96 [PMID:23506865]

Wright EM et al. (2011) Biology of human sodium glucose transporters. Physiol. Rev. 91: 733‐94 [PMID:21527736]

## SLC51 family of steroid‐derived molecule transporters

### Overview

The SLC51 organic solute transporter family of transporters is a pair of heterodimeric proteins which regulate bile salt movements in the bile duct, small intestine and kidney, and elsewhere, as part of the enterohepatic circulation [28, 109]. OST*α*/OST*β* is also expressed in steroidogenic cells of the brain and adrenal gland, where it may contribute to steroid movement [154]. Bile acid transport is suggested to be facilitative and independent of sodium, potassium, chloride ions or protons [28, 109]. OST*α*/OST*β* heterodimers have been shown to transport [^3^H]taurocholic acid, [^3^H]dehydroepiandrosterone sulphate, [^3^H]estrone‐3‐sulphate, [^3^H]pregnenolone sulphate and [^3^H]dehydroepiandrosterone sulphate [28, 109, 154]. OST*α* is suggested to be a seven TM protein, while OST*β* is a single TM ‘ancillary’ protein, both of which are thought to have intracellular C‐termini [315]. Bimolecular fluorescence complementation studies suggest the possibility of OST*α* homo‐oligomers, as well as OST*α*/OST*β* hetero‐oligomers [92, 315].

**Table:** 

Nomenclature	Organic solute transporter subunit *α*	Organic solute transporter subunit *β*
Systematic nomenclature	SLC51A1	SLC51A1BP
Common abreviation	OST*α*	OST*β*
HGNC, UniProt	SLC51A, Q86UW1	SLC51B, Q86UW2

### Further Reading

Ballatori N. (2011) Pleiotropic functions of the organic solute transporter Ost*α*‐Ost*β*. Dig Dis 29: 13‐7 [PMID:21691099]

Ballatori N et al. (2013) The heteromeric organic solute transporter, OST*α*‐OST*β*/SLC51: a transporter for steroid‐derived molecules. Mol. Aspects Med. 34: 683‐92 [PMID:23506901]

Dawson PA. (2011) Role of the intestinal bile acid transporters in bile acid and drug disposition. Handb Exp Pharmacol 169‐203 [PMID:21103970]

## SLC52 family of riboflavin transporters

### Overview

riboflavin, also known as vitamin B2, is a precursor of the enzyme cofactors flavin mononucleotide (FMN) and flavin adenine dinucleotide (FAD). Riboflavin transporters are predicted to possess 10 or 11 TM segments.

**Table:** 

Nomenclature	solute carrier family 52	solute carrier family 52	solute carrier family 52
	(riboflavin transporter), member 1	(riboflavin transporter), member 2	(riboflavin transporter), member 3
Systematic nomenclature	SLC52A1	SLC52A2	SLC52A3
Common abreviation	RFVT1	RFVT2	RFVT3
HGNC, UniProt	SLC52A1, Q9NWF4	SLC52A2, Q9HAB3	SLC52A3, Q9NQ40
Endogenous substrates	riboflavin (*K*_m_1.3 × 10^−3^M) [530]	riboflavin (*K*_m_9.8 × 10^−4^M) [530]	riboflavin (*K*_m_3.3 × 10^−4^M) [530]
Stoichiometry	Unknown	Unknown	H^+^‐dependent

### Comments

Although expressed elsewhere, RFVT3 is found on the luminal surface of intestinal epithelium and is thought to mediate uptake of dietary riboflavin, while RFVT1 and RFVT2 are thought to allow movement from the epithelium into the blood.

### Further Reading

Yamamoto S et al. (2009) Identification and functional characterization of rat riboflavin transporter 2. J. Biochem. 145: 437‐43 [PMID:19122205]

Yonezawa A et al. (2013) Novel riboflavin transporter family RFVT/SLC52: identification, nomenclature, functional characterization and genetic diseases of RFVT/SLC52. Mol. Aspects Med. 34: 693‐701 [PMID:23506902]

## Patched family

### Overview

NPC1L1 acts in the gut epithelium to allow the accumulation of dietary cholesterol through a clathrin‐dependent mechanism. Ezetimibe is used to reduce cholesterol absorption through inhibition of NPC1L1.

**Table:** 

Nomenclature	NPC1‐like 1
HGNC, UniProt	NPC1L1, Q9UHC9
Selective antagonists	ezetimibe (Inhibition) (p*K*_d_ 6.7) [21]

## SLCO family of organic anion transporting polypeptides

### Overview

The SLCO superfamily is comprised of the organic anion transporting polypeptides (OATPs). The 11 human OATPs are divided into 6 families and ten subfamilies based on amino acid identity. These proteins are located on the plasma membrane of cells throughout the body. They have 12 TM domains and intracellular termini, with multiple putative glycosylation sites. OATPs mediate the sodium‐independent uptake of a wide range of amphiphilic substrates, including many drugs and toxins. Due to the multispecificity of these proteins, this guide lists classes of substrates and inhibitors for each family member. More comprehensive lists of substrates, inhibitors, and their relative affinities may be found in the review articles listed below.

**Table:** 

Nomenclature	OATP1A2	OATP1B1	OATP1B3	OATP1C1
Systematic nomenclature	SLCO1A2	SLCO1B1	SLCO1B3	SLCO1C1
HGNC, UniProt	SLCO1A2, P46721	SLCO1B1, Q9Y6L6	SLCO1B3, Q9NPD5	SLCO1C1, Q9NYB5
Substrates	fluoroquinolones, beta blockers, deltorphin II, rosuvastatin, fexofenadine, bromsulphthalein, anticancer drugs, antibiotics, HIV protease inhibitors, talinolol, ouabain, microcystin	statins, opioids, *β*‐lactam antibiotics, bile acid derivatives and conjugates, bromsulphthalein, anticancer drugs, HIV protease inhibitors, fexofenadine, antifungals, ACE inhibitors, rifampicin, endothelin receptor antagonists, sartans	rifampicin, opioids, sartans, statins, digoxin, anticancer drugs, bromsulphthalein, bile acid derivatives and conjugates, *β*‐lactam antibiotics, ouabain, amanitin, saquinavir, fexofenadine, erythromycin‐A, phalloidin	statins, bromsulphthalein
Endogenous substrates	bile acids, thyroid hormones, steroid conjugates, bilirubin, PGE_2_	leukotrienes, steroid conjugates, thyroid hormones, bile acids, bilirubin	steroid conjugates, thyroid hormones, bile acids, CCK‐8 (CCK, P06307), bilirubin, LTC_4_	thyroid hormones, steroid conjugates
Ligands	–	pravastatin (Binding)	–	–
Inhibitors	naringin [25], rifampicin, rifamycin SV	cyclosporin A, gemfibrozil [364], glycyrrhizin, indocyanine green, rifampicin, rifamycin SV, sildenafil	cyclosporin A (pIC_50_ 6.1) [478], sildenafil (pIC_50_ 6.1) [478], rifampicin (pIC_50_ 5.8) [478], gemfibrozil, glycyrrhizin, rifamycin SV	DPDPE, probenecid, taurocholic acid
Labelled ligands	[^3^H]BSP, [^3^H]DPDPE, [^3^H]estrone‐3‐sulphate	[^3^H]estradiol‐17*β*‐glucuronide, [^3^H]estrone‐3‐sulphate	[^3^H]BSP, [^3^H]CCK‐8 (human, mouse, rat), [^3^H]estradiol‐17*β*‐glucuronide	[^125^I]thyroxine, [^3^H]BSP, [^3^H]estrone‐3‐sulphate
Comments	Although rat and mouse OATP1A4 are considered the orthologs of human OATP1A2 we do not cross‐link to gene or protein databases for these since in reality there are five genes in rodents that arose through gene duplication in this family and it is not clear which one of these is the "true" ortholog.	Other inhibitors include, fibrates, flavonoids, glitazones and macrolide antibiotics. pravastatin is used as a probe	Other inhibitors include, HIV protease inhibitors, glitazones and macrolide antibiotics	–

**Table:** 

Nomenclature	OATP2A1	OATP2B1	OATP3A1	OATP4A1	OATP4C1
Systematic nomenclature	SLCO2A1	SLCO2B1	SLCO3A1	SLCO4A1	SLCO4C1
HGNC, UniProt	SLCO2A1, Q92959	SLCO2B1, O94956	SLCO3A1, Q9UIG8	SLCO4A1, Q96BD0	SLCO4C1, Q6ZQN7
Substrates	synthetic prostaglandin derivatives	amiodarone, bromsulphthalein, statins, glibenclamide, aliskiren, fexofenadine, talinolol, bosentan, telmisartan	–	penicillin G	dipeptidyl peptidase‐4 inhibitors, anticancer drugs, cardiac glycosides
Endogenous substrates	prostaglandins, eicosanoids	estrone‐3‐sulphate, dehydroepiandrosterone sulphate, T_4_	BQ123, vasopressin (AVP, P01185), thyroid hormones, prostaglandins	thyroid hormones, prostaglandins, bile acids, steroid conjugates	thyroid hormones, cyclic AMP, steroid conjugates
Inhibitors	bromocresol green (Inhibition of PGF_2*α*_ uptake in PGT‐expressing HeLa cells) (p*K*_i_ 5.4) [263] – Rat, bromsulphthalein (Inhibition of PGF_2*α*_ uptake in PGT‐expressing HeLa cells) (p*K*_i_ 5.2) [263] – Rat	gemfibrozil, glibenclamide, rifamycin SV	–	–	–
Labelled ligands	[^3^H]PGE_2_ (Binding) [78]	[^3^H]BSP, [^3^H]estrone‐3‐sulphate	[^3^H]PGE_2_, [^3^H]estrone‐3‐sulphate	[^3^H]estrone‐3‐sulphate	[^3^H]digoxin
Comments	Other inhibitors include NSAIDs	Other inhibitors include glitazones and citrus juices	–	–	–

### Further Reading

Gong IY et al. (2013) Impact of genetic variation in OATP transporters to drug disposition and response. Drug Metab. Pharmacokinet. 28: 4‐18 [PMID:23047721]

Hagenbuch B et al. (2013) The SLCO (former SLC21) superfamily of transporters. Mol. Aspects Med. 34: 396‐412 [PMID:23506880]

König J et al. (2013) Transporters and drug‐drug interactions: important determinants of drug disposition and effects. Pharmacol. Rev. 65: 944‐66 [PMID:23686349]

Obaidat A et al. (2012) The expression and function of organic anion transporting polypeptides in normal tissues and in cancer. Annu. Rev. Pharmacol. Toxicol. 52: 135‐51 [PMID:21854228]

Roth M et al. (2012) OATPs, OATs and OCTs: the organic anion and cation transporters of the SLCO and SLC22A gene superfamilies. Br. J. Pharmacol. 165: 1260‐87 [PMID:22013971]

Shitara Y et al. (2013) Clinical significance of organic anion transporting polypeptides (OATPs) in drug disposition: their roles in hepatic clearance and intestinal absorption. Biopharm Drug Dispos 34: 45‐78 [PMID:23115084]

Stieger B et al. (2014) Organic anion‐transporting polypeptides. Curr Top Membr 73: 205‐32 [PMID:24745984]
